# Diffusion imaging genomics provides novel insight into early mechanisms of cerebral small vessel disease

**DOI:** 10.1038/s41380-024-02604-7

**Published:** 2024-05-29

**Authors:** Quentin Le Grand, Ami Tsuchida, Alexandra Koch, Mohammed-Aslam Imtiaz, N. Ahmad Aziz, Chloé Vigneron, Laure Zago, Mark Lathrop, Alexandre Dubrac, Thierry Couffinhal, Fabrice Crivello, Paul M. Matthews, Aniket Mishra, Monique M. B. Breteler, Christophe Tzourio, Stéphanie Debette

**Affiliations:** 1 University of Bordeaux, INSERM, Bordeaux Population Health research center, UMR1219, F-33000 Bordeaux, France; 2 Population Health Sciences, German Center for Neurodegenerative Diseases (DZNE), Bonn, Germany; 3 University of Bordeaux, Institute of Neurodegenerative Diseases, UMR5293, Neurofunctional Imaging Group, F-33000 Bordeaux, France; 4 CNRS, Institute of Neurodegenerative Diseases, UMR5293, Neurofunctional Imaging Group, F-33000 Bordeaux, France; 5 CEA, Institute of Neurodegenerative Diseases, UMR5293, Neurofunctional Imaging Group, F-33000 Bordeaux, France; 6 Department of Neurology, Faculty of Medicine, University of Bonn, Bonn, Germany; 7 Department of Human Genetics, McGill University, Montreal, Quebec, Canada; Victor Phillip Dahdaleh Institute of Genomic Medicine at McGill University, Montreal, QC H3A 0G1 Canada; 8 Centre de Recherche, CHU Sainte-Justine, Montréal, QC Canada; 9 Département de Pathologie et Biologie Cellulaire, Université de Montréal, Montréal, QC Canada; 10 Département d’Ophtalmologie, Université de Montréal, Montréal, QC Canada; 11 University of Bordeaux, INSERM, Biologie des maladies cardiovasculaires, U1034, F-33600 Pessac, France; 12 UK Dementia Research Institute and Department of Brain Sciences, Imperial College, London, UK; 13 Institute for Medical Biometry, Informatics and Epidemiology (IMBIE), Faculty of Medicine, University of Bonn, Bonn, Germany; 14 Bordeaux University Hospital, Department of Medical Informatics, F-33000 Bordeaux, France; 15 Bordeaux University Hospital, Department of Neurology, Institute for Neurodegenerative Diseases, F-33000 Bordeaux, France

**Keywords:** Genetics, Biomarkers

## Abstract

Cerebral small vessel disease (cSVD) is a leading cause of stroke and dementia. Genetic risk loci for white matter hyperintensities (WMH), the most common MRI-marker of cSVD in older age, were recently shown to be significantly associated with white matter (WM) microstructure on diffusion tensor imaging (signal-based) in young adults. To provide new insights into these early changes in WM microstructure and their relation with cSVD, we sought to explore the genetic underpinnings of cutting-edge tissue-based diffusion imaging markers across the adult lifespan. We conducted a genome-wide association study of neurite orientation dispersion and density imaging (NODDI) markers in young adults (i-Share study: *N* = 1 758, (mean[range]) 22.1[18–35] years), with follow-up in young middle-aged (Rhineland Study: *N* = 714, 35.2[30–40] years) and late middle-aged to older individuals (UK Biobank: *N* = 33 224, 64.3[45–82] years). We identified 21 loci associated with NODDI markers across brain regions in young adults. The most robust association, replicated in both follow-up cohorts, was with Neurite Density Index (NDI) at chr5q14.3, a known WMH locus in *VCAN*. Two additional loci were replicated in UK Biobank, at chr17q21.2 with NDI, and chr19q13.12 with Orientation Dispersion Index (ODI). Transcriptome-wide association studies showed associations of *STAT3* expression in arterial and adipose tissue (chr17q21.2) with NDI, and of several genes at chr19q13.12 with ODI. Genetic susceptibility to larger WMH volume, but not to vascular risk factors, was significantly associated with decreased NDI in young adults, especially in regions known to harbor WMH in older age. Individually, seven of 25 known WMH risk loci were associated with NDI in young adults. In conclusion, we identified multiple novel genetic risk loci associated with NODDI markers, particularly NDI, in early adulthood. These point to possible early-life mechanisms underlying cSVD and to processes involving remyelination, neurodevelopment and neurodegeneration, with a potential for novel approaches to prevention.

## Introduction

Cerebral small vessel disease (cSVD) is a leading cause of stroke and cognitive decline, and likely the main pathological substrate underlying the vascular contribution to dementia [1]. It is also a common cause of gait, balance, and mood disorders in older persons [2]. This condition is most often covert, i.e. detectable on brain imaging in individuals with no apparent neurological history. MRI-markers of cSVD are extremely common in the general population with increasing age, with a prevalence and burden rising more drastically after age 65 [3]. Extensive covert cSVD was shown in numerous longitudinal studies to portend a two- to three-fold increased risk of stroke and dementia [4, 5]. White matter hyperintensities (WMH), the most common neuroimaging feature of cSVD, was shown to be associated with an increased risk of Alzheimer disease, with evidence for a causal relation using Mendelian randomization [4, 6, 7]. Thus covert cSVD should be a major target to prevent stroke and dementia in the population, an opportunity that has been largely neglected to date. While optimal management of vascular risk factors, especially hypertension, was shown to slow down the progression of cSVD [8], an important limitation is the lack of mechanism-based drugs, targeting the disease process underlying cSVD. Identifying novel therapeutic targets requires better understanding of molecular pathways involved.

Genome-wide association studies (GWAS) are a powerful tool to unravel molecular mechanisms underlying complex diseases. In recent years they identified numerous common genetic variants associated with MRI-markers of cSVD, such as WMH volume [6, 9, 10]. Intriguingly, we recently showed that genetic risk variants for WMH volume identified in older persons are associated with changes in white matter microstructure on diffusion tensor imaging (DTI) already at age 20, in a direction compatible with changes shown to precede the occurrence of WMH in older age (reduced fractional anisotropy and increased mean diffusivity and peak width of skeletonized mean diffusivity) [6, 11, 12]. This suggests that processes contributing to cSVD may find their root much earlier in life than previously thought [6, 13, 14], also dovetailing with emerging evidence that late-life neurodegenerative disorders have early-life and neurodevelopmental determinants amenable to treatment [15, 16].

In contrast with “traditional” MRI-markers of cSVD, such as WMH, lacunes, or cerebral microbleeds, reflecting advanced tissue damage and detectable mostly in late middle to older age, markers of white matter microstructure based on diffusion MRI (dMRI) may capture changes predisposing to cSVD much earlier in life. This could be useful for unraveling molecular mechanisms leading to cSVD throughout the life course, with possible implications for prevention and treatment much earlier in the disease process. DTI, the most commonly used type of dMRI, measures variations in magnitude or directionality of diffusivity but cannot distinguish underlying biological processes (signal-based) [17–19]. More recently, novel biophysical, tissue-based diffusion models derived from multi-shell acquisitions have been developed, such as neurite orientation dispersion and density imaging (NODDI) to provide better descriptions of the underlying tissue properties [17, 20]. NODDI metrics include proxies for the density of neurites (Neurite Density Index, NDI) relative to extra-neurite volume (such as extracellular matrices, microglia and astrocytes), the dispersion of neurite orientation (Orientation Dispersion Index, ODI), and proportion of free water (Isotropic Volume Fraction, ISOVF), i.e. CSF [20, 21].

A recent GWAS in older UK Biobank participants (mean age 64.3 years) [22] identified numerous loci associated with regional NODDI markers. However, to our knowledge, genetic determinants of NODDI markers in younger age groups are unknown. Here we sought to identify loci associated with NODDI markers of white matter microstructure specifically in young adults, when brain white matter maturation peaks. We were then interested in exploring the relation of identified NODDI loci with cSVD, as well as other later onset neurological diseases, given the aforementioned evidence on the role of early life factors. Moreover, we explored how vascular risk factors, which are crucial determinants of cSVD and brain health at large, may already impact white matter microstructure in early adulthood.

## Materials and methods

### Study population

To explore the genetic determinants of NODDI markers in young adults we used the Internet-based Students HeAlth Research Enterprise (i-Share) study, a prospective population-based cohort study of French-speaking students [23]. Here we used the sub-sample of 1758 participants aged 18–35 years for whom both brain MRI and genome-wide genotype data were available, through the MRi-Share and bio-Share ancillary studies (mean age ± standard deviation (SD): 22.1 ± 2.3 years; 72.2% women) [19, 24, 25].

For follow-up in young middle-aged adults of genetic associations observed in i-Share, we used the Rhineland Study, an ongoing community-based prospective cohort study that invites inhabitants aged 30 years and above living in the city of Bonn, Germany, to participate [26]. We used baseline data of a sub-sample of 714 Rhineland Study participants aged 30–40 years with both genotype data and MRI scans available, and no neurological disorder (mean age ± SD: 35.2 ± 3.1 years; 54% women).

Detailed information on both cohorts is presented in the Supplementary methods.

For follow-up in late middle-aged to older adults of genetic associations observed in i-Share, we used summary statistics from the latest published GWAS on NODDI markers in the UK Biobank (*N* = 33,224, 52.4% women, mean age: 64.3 [range, 45.1–81.8] years) [22].

All human research presented in this manuscript was approved by relevant ethics committees and/or institutions and was conducted according to the Declaration of Helsinki. All participants provided written informed consent.

### MRI acquisition and phenotyping

Similar scanners (3-Tesla Siemens Prisma for the i-Share and Rhineland studies and 3-Tesla Siemens Skyra for UK Biobank) and similar diffusion MRI protocols were used for the three cohorts, described in the Supplementary methods and in detail elsewhere [19, 22, 24, 26–29]. To generate regional NODDI phenotypes, all cohorts used the standard tract-based spatial statistics (TBSS) framework and the JHU ICBM DTI-81 atlas [20, 22, 26, 27, 29–37]. This atlas is a stereotaxic probabilistic white matter atlas that fuses DTI-based white matter information with an anatomical template (ICBM-152). The derived white matter parcellation map enables to define 27 white matter anatomic structures segmented based on fiber orientation information (Table S1). In the i-Share and Rhineland studies, we also generated one global measure across the full white matter, in addition to the 27 regional markers (Table S1) for each of the three NODDI metrics (NDI, ODI, ISOVF), leading to 84 NODDI markers in total (Supplementary methods). To normalize distributions we applied a rank-based inverse normal transformation to the 84 variables in both cohorts. For UK Biobank, we derived data for the three NODDI metrics in the same 27 regions, for 21 of which GWAS summary statistics of lateralized values only (left and right) were provided, leading to 144 NODDI markers (no global measure was available) [22, 27, 28].

### Genotyping, quality control, and imputation

Genome-wide genotyping was performed using the Affymetrix Precision Medicine Axiom Array for i-Share, Affymetrix UK BiLEVE Axiom Array and UK Biobank Axiom Array for UK Biobank, and the Infinium Omni2.5Exome-8 BeadChip for Rhineland. Genotypes were imputed to the Haplotype Reference Consortium (HRC, i-Share, UK Biobank) and 1000 Genomes p3v5 (Rhineland Study) reference panels. Quality control procedures were described in the Supplementary methods and in detail elsewhere [25, 38, 39].

### Statistical analyses

**Fig. 1:**
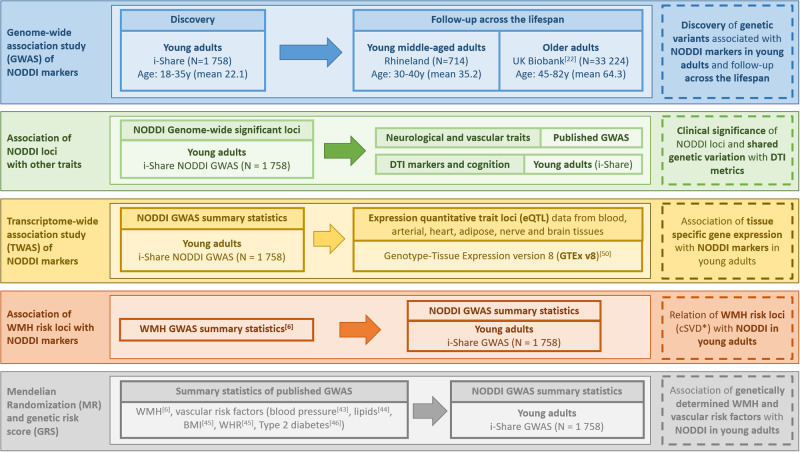
Study workflow. * WMH risk loci reflect genetic susceptibility to cerebral small vessel disease (cSVD). BMI Body mass index, DTI Diffusion tensor imaging, NODDI Neurite orientation dispersion and density imaging, WHR Waist-to-hip ratio adjusted for BMI; WMH White matter hyperintensities.

Analytical steps are summarized in Fig. 1.

### GWAS

We performed 84 GWAS using genome-wide linear mixed models implemented in REGENIE v2.2 [40]. Analyses were restricted to Single Nucleotide Polymorphisms (SNPs) with an imputation score >0.5 and a minor allele frequency (MAF) > 0.01 and adjusted for age at MRI, sex (reported and concordant with genetically determined sex), total intracranial volume and the first four principal components of population stratification (details in Supplementary methods) [25].

Using matSPDlite in R to account for correlation between the 84 NODDI markers, we identified 39.45 independent markers, leading to a significance threshold of *p* < 1.27 × 10^−9^ [41]. We also considered as “suggestive” SNPs reaching genome-wide significance at *p* < 5 × 10^−8^.

In middle-aged adults of the Rhineland Study we followed up NODDI-associated loci identified in i-Share using the same linear mixed model, adjusting for age at MRI, sex, intracranial volume and the first ten principal components of population stratification [42]. When the lead SNP from the i-Share GWAS was not available, we used the best LD-proxy (LD-r² > 0.50). Next, to expand our results to older adults, we followed up NODDI-associated loci from i-Share in the latest NODDI GWAS from UK Biobank (*N* = 33,224) [22]. SNPs with p < 2.38 × 10^−3^ (correcting for 21 independent loci) were considered replicated. We also performed sex-specific association analyses for genome-wide significant NODDI-associated SNPs in the three samples.

#### Shared genetic variation of NODDI markers with neurological and vascular traits

We explored the association of genome-wide significant NODDI loci identified in i-Share (lead SNPs and their LD-proxies in a 1 Mb window and with r² > 0.9) with several neurological and vascular traits, using the largest available European ancestry GWAS summary statistics thereof: blood pressure (systolic [SBP], diastolic blood pressure [DBP] and pulse pressure [PP], *N* = 757,601) [43], lipids (LDL-, HDL-cholesterol, triglycerides [TG], *N* = 1,320,016) [44], body mass index (BMI) (*N* = 806,834) [45], waist-to-hip ratio adjusted for BMI (WHR) (*N* = 694,649) [45], type 2 diabetes (*N* = 74,124/824,006) [46], WMH (*N* = 50,970) [6], Alzheimer disease (*N* = 111,326/677,663) [47], and multiple sclerosis (MS) (*N* = 14,802/26,703) [48]. Genetic associations with these traits at *p* < 1.98 × 10^−4^ (Bonferroni-corrected threshold for 21 loci and 12 traits) were considered statistically significant.

#### Association of NODDI loci with cognitive performance and with diffusion tensor imaging markers in young adults

To explore their clinical relevance in young adults, we examined the association of genome-wide significant NODDI loci identified in i-Share (lead SNPs) with scores of 8 cognitive tests in the same cohort (Supplementary methods). Associations were tested using similar regression models as for the NODDI GWAS, adjusting for age, sex, and the first four principal components of population stratification. Genetic associations at *p* < 2.98×10^-4^ (correcting for 21 loci and 8 traits) were considered significant. We further tested the association of NODDI loci with the two most studied DTI markers in i-Share, fractional anisotropy (FA) and mean diffusivity (MD), in the same regions of interest. Genetic associations at *p* < 1.19 × 10^−3^ (correcting for 21 loci and 2 traits) were considered significant.

#### Transcriptome-wide association study on the genome-wide significant NODDI loci in young adults

We performed transcriptome-wide association studies (TWAS) using TWAS-Fusion [49] to identify genes whose expression is significantly associated with NODDI markers. We focused on genome-wide significant, replicated NODDI GWAS loci. We used precomputed functional weights from publicly available gene expression reference panels (expression quantitative trait loci [eQTL]) from tissues considered relevant for cerebrovascular disease (blood, arterial, heart, adipose, nerve and brain tissues) and cross-tissue weights generated using sparse canonical correlation analysis (sCCA), from the Genotype-Tissue Expression version 8 (GTEx v8) [50, 51]. Transcriptome-wide significance at *p* < 7.8 × 10^−6^ was based on the average number of features (6400 genes) tested across tissues, and we also considered suggestive associations with *p* < 1 × 10^−4^. These genes were then tested in conditional analyses in TWAS-Fusion [49]. Next, we performed a colocalization analysis (COLOC) on the conditionally significant genes (*p* < 0.05) to estimate the posterior probability of a shared causal variant between the gene expression and trait association (PP4) [52], considering genes with PP4 ≥ 0.75 as colocalized. Colocalized genes with eQTLs reaching genome-wide significance in association with the corresponding NODDI marker (or in moderate-high LD, r^2^ > 0.5, with the lead SNP) were considered as being in a GWAS locus, others were considered as pointing to “novel” loci (Supplementary methods).

#### Lifetime brain gene expression profile

We examined the spatio-temporal expression pattern of genes in genome-wide significant NODDI loci identified in i-Share that also colocalized in the TWAS analyses. We used a public database (https://hbatlas.org/) comprising genome-wide exon-level transcriptome data from 1340 tissue samples from 16 brain regions of 57 postmortem human brains, from embryonic development to late adulthood [53].

#### Association of WMH risk loci with NODDI markers in young adults

We explored associations with NODDI markers in young adults of 25 known WMH risk loci previously identified in older European-ancestry populations [6]. We extracted association results of these 25 loci from the GWAS of global NDI, ODI and ISOVF in i-Share, using *p* < 2 × 10^−3^ as the significance threshold.

#### Association of genetically determined WMH and vascular risk factors with NODDI markers in young adults

To examine the association of genetically determined WMH and vascular risk factors with the NODDI markers in i-Share, we first generated weighted genetic risk scores (GRS) using independent (r² > 0.01) genome-wide significant variants (*p* < 5 × 10^-8^) from the largest European-ancestry GWAS summary statistics for blood pressure [43], lipids [44], BMI [45], WHR [45], type 2 diabetes [46], and WMH [6]. Associations were tested using linear mixed models adjusted for age, sex, total intracranial volume, and the first four principal components of population stratification (Supplementary methods). Results with *p* < 1.27 × 10^−3^ (accounting for 39.45 independent NODDI markers) were considered significant [41]. Next, for significant results in GRS analyses, we performed two-sample Mendelian randomization (MR) analyses to seek evidence for a causal relation of WMH and vascular risk factors with NODDI markers (Supplementary methods). To build genetic instruments we used independent (r^2^ > 0.01) genome-wide significant (*p* < 5 × 10^−8^) risk variants as in GRS analyses. We used three distinct two-sample MR approaches to strengthen the validity of our findings: RadialMR [54], Generalised Summary-data-based Mendelian Randomisation (GSMR) [55] and TwoSampleMR [56]. We considered RadialMR after outlier removal and GSMR as primary analyses. In addition to classical inverse-variance weighted (IVW) analyses, we applied MR methods that are more robust to the use of pleiotropic instruments (weighted median, MR-Egger, analyses excluding instruments showing heterogeneity). Given the relatively small sample for MR analyses, we used a significance threshold of *p* < 0.05, in an exploratory setting.

Finally, we examined whether brain regions where genetically determined WMH was associated with NODDI markers in young adults overlap with regions most frequently harboring WMH in older adults. We projected (on axial multi-slices) WMH GRS Z-scores for significant associations with NDI in i-Share onto the corresponding white matter regions, overlaying this projection with the frequency of WMH occurrence across white matter regions in older adults participating in the population-based 3C-Dijon study (*N* = 1781, mean age: 72.4[65–85] years) [57, 58]. We computed the mean WMH frequency for 3C-Dijon participants in each of the 27 JHU regions to test their correlation with Z-scores of association between the WMH GRS and NDI within each JHU region in the i-Share cohort.

## Results

### Genetic susceptibility to tissue-based variations of white matter microstructure in young adults

**Fig. 2:**
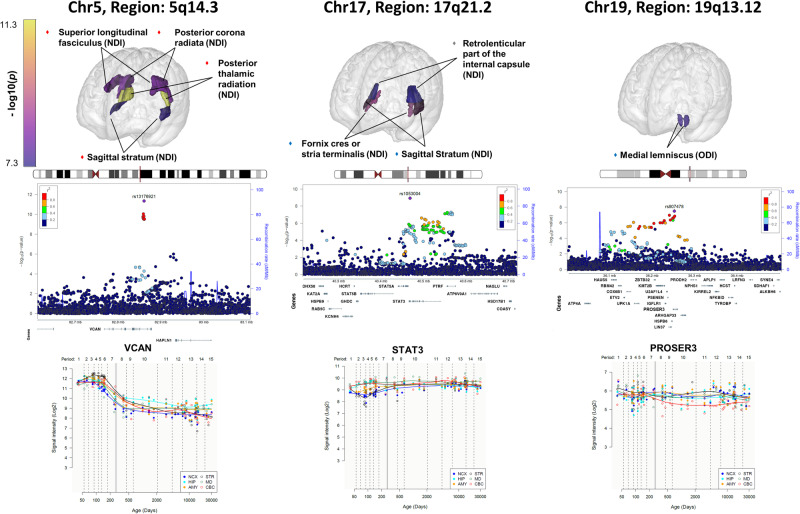
Representation of brain regions showing associations with the replicated loci in NODDI marker GWAS with regional plots and lifetime brain gene expression profile of the nearest genes. The first line shows the localization of the 3 regions with significant and replicated signals in GWAS: chr5q14.3, chr17q21.2 and chr19q13.12. Colors of bullet points on brain projection represent the replication of the loci: red if replicated in all studies, blue for i-Share and UK Biobank, and gray for i-Share only. The second line represents the localization of each locus on the chromosome, combined with a regional plot of the locus. The third line represents the spatio-temporal gene expression level for the nearest gene in each locus (*VCAN*, *STAT3* and *PROSER3* respectively). It is plotted as log2-transformed exon array signal intensity (y-axis) against the post conception days (x-axis) as provided by the Human Brain Transcriptome project database. Periods of human development and adulthood are indicated by vertical dashed lines: 4–8 post conception weeks [PCW] (period 1), 8–10 PCW (period 2), 10–13 PCW (period 3), 13–16 PCW (period 4), 16–19 PCW (period 5), 19-24 PCW (period 6), 24-38 PCW (period 7), birth- 6 postnatal months (period 8), 6–12 postnatal months (period 9), 1–6 years (period 10), 6–12 years (period 11), 12–20 years (period 12), 20–40 years (period 13), 40–60 years (period 14), and 60 years+ (period 15). The boundary between pre- and postnatal periods is indicated by the solid vertical line. Each colored point represents the expression level of each gene across 16 anatomical brain regions and ages. Brain structure includes 11 neocortical areas (NCX, blue), and 5 subcortical regions: hippocampus (HIP, cyan), amygdala (AMY, orange), striatum (STR, black), mediodorsal nucleus of thalamus (MD, dark green), and cerebellar cortex (CBC, red). NDI Neurite Density Index, ODI Orientation Dispersion Index, ISOVF Isotropic Volume Fraction.

Using GWAS for 84 NODDI markers in 1 758 young adults from the i-Share study (mean age 22.1), we identified 27 genome-wide significant associations (*p* < 5 × 10^−8^) in 21 genomic loci (Table 1, Fig. 2, Figs. S1–2). Two of these remained significant after additionally correcting for the number of independent NODDI markers tested (*p* < 1.27 × 10^−9^), at chr5q14.3 in *VCAN*, associated with NDI in the posterior thalamic radiation (PTR, *p* = 4.50 × 10^−12^), and at chr17q21.2 in *STAT3*, associated with NDI in the fornix cres or stria terminalis (FX.ST, *p* = 1.16 × 10^−9^). The chr5q14.3 locus was associated with NDI at *p* < 5 ×10^-8^ in several other regions besides PTR, i.e. the posterior corona radiata (PCR), superior longitudinal fasciculus (SLF) and sagittal stratum (SS), and with global NDI. Associations with NDI markers at chr5q14.3 replicated (*p* < 2.38 × 10^−3^) both in the Rhineland Study and UK Biobank (mean age 35.2 and 64.3 years), even reaching genome-wide significance in the latter. Two additional genome-wide significant loci identified in i-Share replicated in the UK Biobank, at chr17q21.2 (*STAT3*) for NDI-FX.ST and NDI-SS, and at chr19q13.12 (in *PROSER3*) with ODI in the medial lemniscus (ML). In sensitivity analyses adding a head motion parameter as a covariate, associations were substantially unchanged (Fig. S3).

**Table 1:** Genome-wide significant SNPs associated with NODDI markers in the i-Share cohort and replication in the Rhineland study and UK Biobank.

								i-Share [18–35 y] (*n* = 1 758)				RLS [30–40 y] (*n* = 714)			UKB [45–82 y] (*n* = 33 224)		
Region of interest	SNP	Locus	Position	Nearest genes	Function	A0	A1	Freq	Beta	SE	p	Beta	SE	p	Beta	SE	p
**Global metrics**																	
ISOVF	rs4889450	16q23.3	82041436	*SDR42E1*	Intronic	G	T	0.09	0.32	0.06	2.80E-08						
**NDI**	rs10052710	5q14.3	82860025	*VCAN*	Intronic	G	T	0.80	0.23	0.04	2.32E-09	**0.21**	**0.05**	**6.85E-05**			
ODI	rs9819179	3q13.31	115173615	*ZBTB20;GAP43*	Intergenic	A	G	0.95	0.37	0.07	2.01E-08	-0.08	0.09	3.53E-01			
**Specific regions of interest**																	
**ISOVF**																	
Middle cerebellar peduncle	rs77827241	3q28	191973700	*FGF12-AS1*	ncRNA intronic	T	C	0.04	0.47	0.08	2.09E-08				−0.02	0.02	4.16E-01
Posterior corona radiata	rs2710548	4q28.1	126273530	*FAT4*	Intronic	C	T	0.58	0.17	0.03	2.49E-08	-0.08	0.04	5.62E-02	−0.01	0.01	3.96E-01
External capsule	rs3775205	4q34.3	177590724	*SPCS3;VEGFC*	Intergenic	C	T	0.20	0.21	0.04	4.33E-08	0.02	0.04	6.65E-01	0.00	0.01	7.32E-01
Middle cerebellar peduncle	rs7971607	12q24.31	122330480	*PSMD9*	Intronic	A	G	0.43	0.17	0.03	6.18E-09	0.04	0.04	3.36E-01	0.00	0.01	6.20E-01
Cingulum cingulate gyrus	rs9904001	17q24.1	63309198	*RGS9;LINC02563*	Intergenic	G	A	0.80	0.22	0.04	4.05E-09	0.04	0.05	4.81E-01	0.00	0.01	9.57E-01
Uncinate fasciculus	rs8101200	19q13.31	44051558	*XRCC1*	Intronic	G	A	0.12	0.28	0.05	1.04E-08	0.08	0.07	2.77E-01	0.03	0.01	7.30E-02
**NDI**																	
Retrolenticular part of the internal capsule	rs72642850	1p36.23	7526759	*CAMTA1*	Intronic	A	G	0.16	0.21	0.04	3.27E-08	0.00	0.05	9.74E-01	0.01	0.01	4.27E-01
**Posterior corona radiata**	rs13176921	5q14.3	82860348	*VCAN*	Intronic	G	A	0.79	0.22	0.04	4.67E-09	**0.19**	**0.05**	**4.62E-04**	**0.16**	**0.01**	**2.94E-57**
**Posterior thalamic radiation**	rs13176921	5q14.3	82860348	*VCAN*	Intronic	G	A	**0.79**	**0.27**	**0.04**	**4.50E-12**	**0.16**	**0.05**	**1.70E-03**	**0.18**	**0.01**	**6.68E-75**
**Superior longitudinal fasciculus**	rs13176921	5q14.3	82860348	*VCAN*	Intronic	G	A	0.79	0.22	0.04	4.59E-09	**0.18**	**0.05**	**3.55E-04**	**0.16**	**0.01**	**1.66E-60**
**Sagittal stratum**	rs13176921	5q14.3	82860348	*VCAN*	Intronic	G	A	0.79	0.20	0.04	3.73E-08	**0.16**	**0.05**	**1.84E-03**	**0.19**	**0.01**	**3.63E-84**
Posterior corona radiata	rs13257545	8q12.1	56531057	*XKR4;TMEM68*	Intergenic	A	G	0.07	0.34	0.06	3.35E-08	-0.04	0.09	6.17E-01	−0.01	0.02	6.90E-01
**Fornix cres or stria terminalis**	rs1053004	17q21.2	40466092	*STAT3*	UTR3	A	G	**0.40**	**0.18**	**0.03**	**1.16E-09**	0.02	0.04	6.00E-01	**0.03**	**0.01**	**2.01E-03**^**b**^
Retrolenticular part of the internal capsule	rs1053004	17q21.2	40466092	*STAT3*	UTR3	A	G	0.40	0.17	0.03	4.71E-08	0.00	0.04	9.07E-01	0.02	0.01	5.05E-02
**Sagittal stratum**	rs1053004	17q21.2	40466092	*STAT3*	UTR3	A	G	0.40	0.18	0.03	2.39E-08	-0.02	0.04	6.32E-01	**0.03**	**0.01**	**1.06E-03**^**b**^
Posterior corona radiata	rs17833531^a^	18q22.2	68348916	*GTSCR1;LINC01541*	Intergenic	A	G	0.92	0.33	0.06	2.80E-08	0.06	0.06	3.20E-01	0.01	0.01	5.15E-01
**ODI**																	
Anterior corona radiata	rs12126432	1p13.1	116595405	*SLC22A15*	Intronic	G	C	0.97	0.60	0.11	3.12E-08	0.09	0.10	3.49E-01	0.02	0.02	3.00E-01
Cingulum cingulate gyrus	rs4920174	1q42.2	234054627	*SLC35F3*	Intronic	T	C	0.98	0.86	0.15	1.20E-08						
Superior fronto-occipital fasciculus	rs10516765	4q21.23	86847135	*ARHGAP24*	Intronic	A	G	0.99	0.91	0.15	3.70E-09						
Sagittal stratum	rs11185683^a^	9q34.2	137131455	*RNU6ATAC;LINC02247*	Intergenic	A	G	0.96	0.54	0.09	1.43E-08	-0.04	0.13	7.72E-01	0.01	0.02	5.39E-01
Anterior corona radiata	rs4968557	17q23.2	59462087	*BCAS3*	Intronic	A	G	0.87	0.29	0.05	2.96E-08	0.02	0.08	7.73E-01	−0.01	0.02	7.22E-01
**Medial lemniscus**	rs807478^a^	19q13.12	36252494	*PROSER3*	Intronic	G	A	0.49	0.15	0.03	3.12E-08	0.05	0.04	2.41E-01	**0.03**	**0.01**	**3.05E-05**
Cingulum cingulate gyrus	rs56083857	22q11.23	25386309	*TMEM211;KIAA1671*	Intergenic	T	C	0.89	0.31	0.05	3.76E-09	-0.09	0.07	1.84E-01	0.01	0.01	5.68E-01
Body corpus callosum	rs77240077	22q12.1	26944635	*TPST2*	Intronic	G	A	0.05	0.38	0.07	3.94E-08	0.15	0.10	1.46E-01	−0.03	0.02	1.41E-01

*P* < 1.27 × 10^−9^ for the i-Share study and *p* < 2.38 × 10^−3^ for UK Biobank and Rhineland Study are in bold.

*RLS* Rhineland Study, *UKB* UK Biobank [22], *A1* effect allele, *A0* non-effect allele, *Freq* frequency of A1 in the i-Share study, *Beta* effect for A1, *SE* Standard error, *NDI* Neurite Density Index, *ODI* Orientation Dispersion Index, *ISOVF* Isotropic Volume Fraction.

^a^rs17833531, rs807478 and rs11185683 were not available in the Rhineland study, results are presented for their proxies rs80237634 (LD-r^2^ = 0.58), rs173003 (LD-r² = 0.98) and rs11185685 (LD-r² = 0.99) in this study.

^b^Significant only for left region of interest. For UK Biobank results, the results are presented for the most significant laterality (Left/Right) for lateralized metrics.

Sex-stratified results were comparable in men and women for the 27 genome-wide significant NODDI associations, with the same directions of effect and overlapping confidence intervals, except for a weaker and non-significant association of the chr5q14.3 locus with global NDI in men than women, with a significant sex-interaction in i-Share (Fig. S4). Most genome-wide significant NODDI loci showed significant associations (*p* < 1.19 × 10^-3^) with at least one DTI marker (FA or MD). However, only two NODDI-associated variants at chr5q14.3 also showed genome-wide significant association with FA (global measure) and MD (global measure, and in PCR, PTR, SLF, and SS, Figure S5).

### Clinical and molecular correlates of NODDI-associated variants in young adults

We explored associations of genome-wide significant and replicated NODDI susceptibility loci identified in young adults with neurological and vascular traits, using the largest published GWAS for the latter (Table S2). Alleles associated with lower NDI were associated with larger WMH volume at chr5q14.3 (*VCAN*) and with higher pulse pressure and lower risk of multiple sclerosis (MS) at chr17q21.2 (*STAT3*), all at genome-wide significance.

We also explored associations of the 21 genome-wide significant NODDI loci with cognitive tests in the i-Share study (Table S3). At chr9q34.2 (near *RNU6ATAC*), the allele associated with larger ODI (reflecting that the orientations of the neurites spread out more widely in space) was associated with better performance on the matrices test, capturing reasoning and fluid intelligence. In addition, 6 loci were associated with at least one cognitive test at nominal significance (*p* < 0.05), including the 3 replicated loci: at *VCAN* (borderline at *p* = 0.05) and *STAT3* NDI-lowering alleles were associated with worse performance on processing speed and verbal memory tests; at *PROSER3* ODI-increasing alleles were associated with better performance on a vocabulary test.

**Fig. 3:**
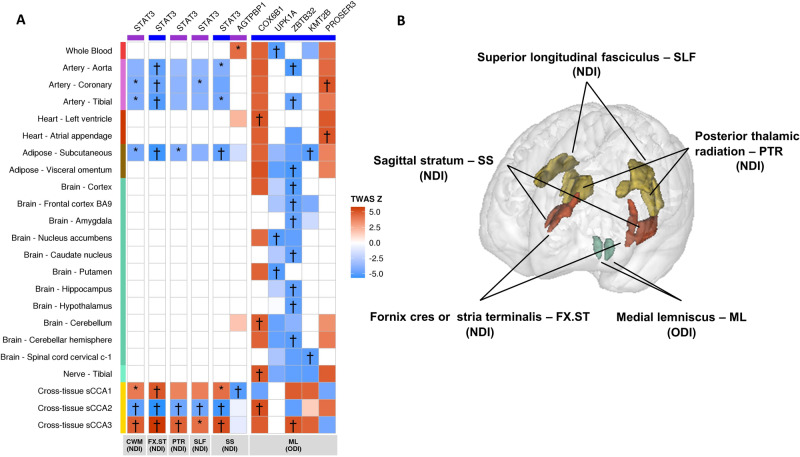
Transcriptome-wide association study (TWAS) of NODDI phenotypes in multiple tissues. **A** Heatmap of the transcriptome-wide association studies of NODDI markers with genome-wide significant loci in the i-Share study and replicated in the Rhineland study or UK Biobank. Colors in squares represent the association Z-statistic of gene expression with NODDI markers. *: TWAS *p* < 1 × 10^−4^, *p* < 0.05 in conditional analyses and COLOC-PP4 > 0.75. † TWAS *p* < 7.8 × 10^−6^, *p* < 0.05 in conditional analyses and COLOC-PP4 > 0.75. Only genes with † in at least one tissue for the corresponding phenotype are shown. Genes are presented on the x-axis, those underlined in blue are in a GWAS locus, those underlined in purple are not; Tissue types are on the y-axis (orange: blood; pink: arterial; dark orange: heart; brown: adipose, green: brain; turquoise blue: nerve; gold: cross-tissue weights). sCCA Sparse canonical correlation analysis. **B** Brain representation of regions presenting associations with † in (**A**), with colors representing the types of tissues as on (**A**). NDI Neurite Density Index, ODI Orientation Dispersion Index, ISOVF Isotropic Volume Fraction.

Next, to explore putative causal genes and directions of effect, we conducted TWAS on the 7 NODDI markers with replicated genome-wide significant loci using TWAS-Fusion and eQTLs based on RNA sequencing in relevant tissues (“Methods”). We identified 32 genes whose genetically regulated expression was associated with NODDI markers (NDI for 19 genes) at *p* < 1 × 10^−4^ with colocalization of GWAS lead variants and eQTL (COLOC-PP4 > 0.75) in at least one tissue (Fig. 3, Fig. S6, Table S4). Higher expression of *STAT3* (at chr17q21.2 NDI locus) in arteries and adipose tissue was significantly associated with lower NDI. Expression levels of five genes at the chr19q13.12 ODI-ML locus (*PROSER3, COX6B1*, *UPK1A*, *ZBTB32*, and *KMT2B*) were significantly associated with ODI-ML in vascular and brain tissues (Fig. 3, Fig. S6, Table S4). Finally, when interrogating the human brain transcriptome atlas from embryonic development to late adulthood [53], expression of transcriptome-wide significant genes was either stable across the full lifespan or increased post-natally, while expression of *VCAN* (chr5q14.3 locus) was considerably higher during the prenatal period (Fig. 2 and Fig. S7).

### Association of cerebral small vessel disease (WMH) risk loci with NODDI markers in young adults

First, we explored individual associations of known genetic risk loci for WMH volume identified in older adults (mean age 65 years) [6] with NODDI markers in young i-Share participants in their twenties. Out of 25 loci, 7 showed nominally significant associations with at least one of the three global NODDI markers (NDI, ODI, ISOVF). Two of these remained significant after multiple testing correction (*p* < 2 × 10^-3^), at chr5q14.3 (*VCAN*) and chr17q21.31 (*NMT1*), the WMH risk allele being associated with lower NDI (Table 2 and Table S5).

**Table 2:** Association of known WMH risk variants and global NODDI markers in young adults (i-Share study, n = 1 758).

							NDI		ODI		ISOVF	
SNP	Locus	Pos	Nearest gene	A1	A0	FreqA1	Z	p	Z	p	Z	p
Genetic risk score (23 SNPs)							**−3.83**	**1.28E-04**^**a**^	1.09	2.76E-01	0.99	3.22E-01
rs73923006	2p21	43132224	*HAAO*	G	C	0.82	−0.01	9.95E-01	**2.10**	**3.59E-02**	1.61	1.08E-01
rs17205972	5q14.3	82859065	*VCAN*	T	G	0.19	**−5.68**	**1.32E-08**^**a**^	0.02	9.81E-01	**2.46**	**1.38E-02**
rs71471298	10q24.33	105507145	*SH3PXD2A-AS1*	T	C	0.11	**−2.61**	**9.18E-03**	0.11	9.16E-01	0.43	6.70E-01
rs10786772	10q24.33	105610326	*SH3PXD2A*	G	A	0.69	−1.68	9.25E-02	0.44	6.61E-01	**2.40**	**1.64E-02**
rs55940034	13q34	111043309	*COL4A2*	G	A	0.31	**2.14**	**3.25E-02**	0.79	4.30E-01	−0.66	5.07E-01
rs1948948	16q12.1	51442679	*SALL1*	C	T	0.58	0.12	9.07E-01	0.51	6.12E-01	**−2.67**	**7.59E-03**
rs6503417	17q21.31	43144218	*NMT1*	C	T	0.63	**−3.41**	**6.46E-04**^**a**^	−0.41	6.81E-01	**2.00**	**4.56E-02**

A1: effect allele (aligned with allele increasing WMH risk); A0: non-effect allele; Freq: frequency of A1; Z: Z-score defined as Beta of A1 / Standard error.

Only results at least nominally significant with one of the global NODDI markers are presented.

Nominally significant results are in bold.

*NDI* Neurite Density Index, *ODI* Orientation Dispersion Index, *ISOVF* Isotropic Volume Fraction.

^a^Significant results (*p* < 2 × 10^-3^ for SNPs; *p* < 1.27 × 10^-3^ for genetic risk score).

**Fig. 4:**
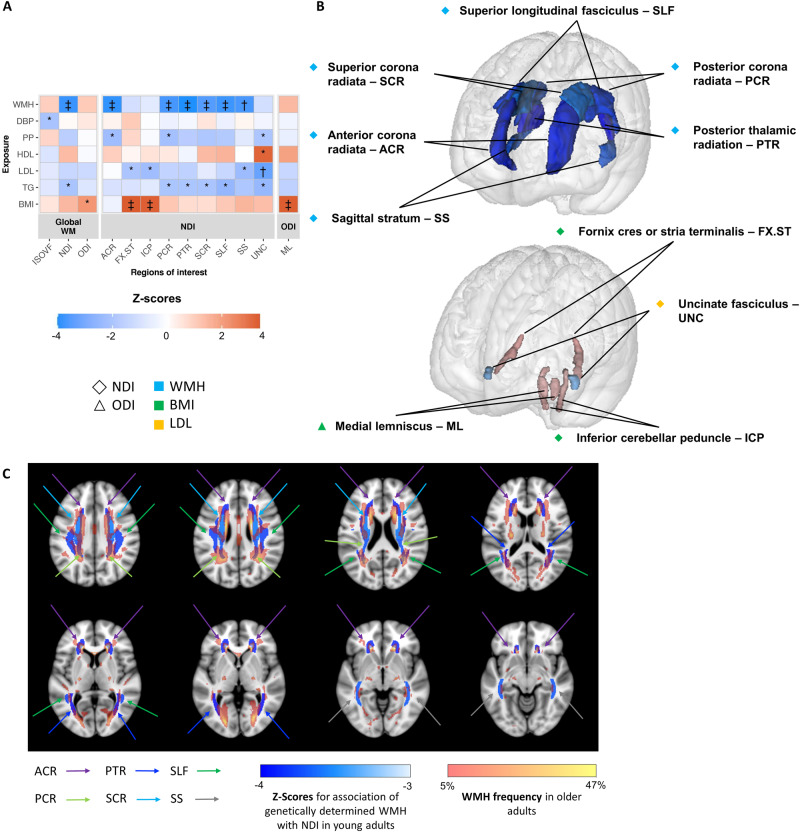
Association of genetically-predicted neurovascular traits and vascular risk factors with NODDI metrics in young adults. **A** Heatmap of the association of neurovascular traits and vascular risk factors with NODDI metrics in young adults using genetic risk score and Mendelian randomization approaches. Only regions of interest with *p* < 1.27 × 10^-3^ in GRS analyses and *p* < 0.05 with at least one method between RadialMR IVW (after removing outliers) and GSMR are shown. Only exposures with *p* < 0.05 in GRS analyses in at least one region of interest are shown. Z-scores correspond to the effect of the GRS of the exposures on the NODDI phenotypes. *p < 0.05 with GRS. ^†^*p* < 1.27 × 10^−3^ with GRS and *p* < 0.05 with at least one method between RadialMR IVW (after removing outliers) and GSMR. ^‡^*p* < 1.27 × 10^-3^ with GRS and *p* < 0.05 with both RadialMR IVW and GSMR. **B** Projection of significant results on the brain map. Only significant results († or ‡ on **A**) are projected on the brain map. For both **A** and **B**, colors depend on the Z-score values. **C** Overlap with brain regions mostly affected by WMH in older age. The same significant regions for NDI results shown on (**B**), on axial multi-slices to show the overlap with regions affected by WMH in older age; blue scale: Z-score values corresponding to the effect of the GRS for WMH on NDI metrics in young adults (i-Share study); pink to yellow scale: frequency of WMH occurrence in older adults in their seventies (3C). ISOVF Isotropic Volume Fraction, NDI Neurite Density Index, ODI Orientation Dispersion Index, WM White matter, WMH White matter hyperintensities, DBP diastolic blood pressure, PP pulse pressure, HDL HDL-cholesterol, LDL LDL-cholesterol, TG triglycerides, BMI body mass index.

Second, we aggregated WMH risk variants in a weighted genetic score to explore their combined association with NODDI markers in young adults. Using both GRS and MR analyses, larger genetically predicted WMH volume was significantly associated with lower NDI, globally and in several regions (Fig. 4, Figs. S8 and S9, Table S6). These associations were found in projection fibers (PCR, PTR, anterior corona radiata (ACR), and superior corona radiata (SCR)) and association fibers (SS and superior longitudinal fasciculus (SLF)). Notably, regions showing associations between genetically predicted WMH and NDI in the young overlapped with regions harboring the highest frequency of WMH in older persons in their seventies (3C-Dijon Study, Fig. 4). Across JHU regions, mean WMH frequency in 3C-Dijon participants correlated significantly with Z-scores of association between the WMH GRS and NDI in i-Share participants (Pearson’s r = −0.54, *p* = 0.0037, Fig. S10).

Finally, in secondary analyses, as WMH share many genetic risk variants with vascular risk factors, especially blood pressure [6], we explored whether observed associations of NDI with genetically predicted WMH in i-Share merely reflect associations with genetically predicted vascular risk factors (Fig. 4, Figs. S8 and S9, Table S6). Genetically determined high blood pressure and genetic liability to any other vascular risk factor were not associated with NDI in i-Share. The only significant associations observed were between smaller genetically determined BMI and lower regional NDI, in different regions than those associated with genetically predicted WMH volume (FX.ST and inferior cerebellar peduncle (ICP), Fig. 4).

## Discussion

In this first genomic study of NODDI diffusion markers in young adults, we identified genetic determinants of brain white matter microstructure in early life and shed new light on their relation with cSVD in older age, a leading cause of stroke and dementia worldwide. In total, 21 independent genetic loci were associated with NODDI markers at genome-wide significance. The most prominent and robust signal was at chr5q14.3 (in *VCAN*), a known risk locus for WMH, associated with lower NDI in the whole brain and in 4 regions involving projection and association fibers. These associations were replicated across the adult lifespan, in young middle-aged adults from the Rhineland Study and older adults from UK Biobank. Additional genome-wide significant NODDI loci were replicated in UK Biobank, for NDI markers at chr17q21.2 (*STAT3*), a known pulse pressure and MS locus, and for ODI markers at chr19q13.12 (*PROSER3*). Interestingly, these three main loci were nominally associated with cognitive performance in young adults. Using TWAS, we identified 32 genes of which the genetically determined expression in vascular or brain tissues was associated with NODDI metrics, with evidence for colocalization: 19 with NDI (including *STAT3* at chr17q21.2) and 13 with ODI (including *PROSER3, COX6B1*, *UPK1A*, *ZBTB32*, and *KMT2B* at chr19q13.12). Among known WMH risk loci previously identified in older adults, besides chr5q14.3 (*VCAN*), the chr17q21.31 (*NMT1*) locus was associated with lower NDI. Genetically predicted larger WMH volume was significantly associated with lower NDI at age 20, specifically in regions harboring the highest frequency of WMH in older age. This was not driven by genetically determined vascular risk factors.

To our knowledge, this is one of the first studies exploring genetic associations with cutting-edge NODDI markers in young adults and across the adult lifespan, in three complementary cohorts using similar MRI scanners, state-of-the-art protocols and phenotype definitions.

Individually, the most robust genetic association with NODDI markers (NDI) across the adult lifespan was with common intronic variants at chr5q14.3, also a risk locus for WMH in older age [6]. Of note, these variants showed weaker associations in men than women in i-Share only, possibly reflecting previously reported sex differences in brain white matter microstructure [59], including during development [60]. Lead variants were located in introns of *VCAN*, encoding versican, a chondroitin sulfate proteoglycan with multiple isoforms. In the brain *VCAN* is expressed primarily in oligodendrocytes and oligodendrocyte precursor cells, fibroblasts (perivascular and meningeal), and to a lesser extent arterial endothelial cells and arteriolar smooth muscle cells [61–63]. Versican is a major component of the extracellular matrix (ECM), playing a key role in tissue morphogenesis and in regulating immunity and inflammation [64]. Along with *COL4A1*, *COL4A2*, *FGA*, and *MMP12, VCAN* is a main hub in the ECM network across the cerebrovascular matrisome [65], which was recently proposed as a converging pathway in monogenic and multifactorial cSVD [9, 65–68]. Recently, versican was also found to be involved in remyelination in MS, with versican isoform V1 inhibiting remyelination by promoting local T helper 17 cytotoxic neuroinflammation, and versican inhibitors were suggested as a potential dual repair and immunomodulatory therapy for MS [69]. *VCAN* expression in adult vascular and brain bulk tissues was not significantly associated with NODDI markers using TWAS. This could be explained by developmental mechanisms underlying observed genetic associations, *VCAN* expression in the brain being highest in early developmental (prenatal) stages (Fig. 2, Fig. S7), or by cell-type specific, or isoform-specific effects [70]. Interestingly however, in UK Biobank, risk alleles for lower NDI were significantly associated with lower plasma versican protein levels (*p* = 8.5 × 10^−25^) measured on the Olink platform [71]. Further explorations, such as single-nuclei isoform RNA sequencing [72], will be required to decipher VCAN isoforms and cell types involved in modulating cSVD risk, to guide experimental follow-up towards potential therapeutic development.

At the second most significant NODDI locus (chr17q21.2) identified in young adults and replicated in older adults, common variants in *STAT3* were associated with NDI in several regions. Higher genetically determined *STAT3* expression in adipose and arterial tissues was associated with lower NDI. Signal transducer and activator of transcription 3 (*STAT3*), encodes a transcription factor expressed in neurons, endothelial cells, astrocytes and microglia [73], activated in response to cytokines and growth factors. *STAT3* plays a key role in neuron and glial cell development, maintenance and survival [74]. The allele associated with lower NDI is known to be associated with higher pulse pressure and lower risk of MS [43, 48]. *STAT3* signaling in myeloid cells promotes pathogenic myelin-specific T-cell differentiation and autoimmune demyelination and was suggested as a therapeutic target for MS [75, 76]. *STAT3* is also involved in the pathogenesis of amyloid deposits in cerebral amyloid angiopathy (a type of cSVD) and Alzheimer’s disease [77]. *STAT3*-specific inhibition in a mouse model of amyloidosis improved cognitive function, functional connectivity and increased cerebral blood flow [77]. Inhibition of *STAT3* was also shown to reduce neonatal hypoxic-ischemic brain damage [73].

Intriguingly, both *VCAN* and *STAT3* encode proteins involved in the demyelination/remyelination process in MS. Several epidemiological observations suggest that vascular risk factors and cSVD may contribute to MS severity [78]. Moreover, cSVD and MS share some pathological features, such as white matter demyelination and brain atrophy [78, 79]. Genetic studies have failed to identify shared genetic variation between MS and cSVD, however they were conducted in older populations, with smaller cSVD GWAS datasets than currently available [80]. Our findings suggest that early variations in brain white matter microstructure known to precede cSVD occurrence could perhaps share some biological pathways with susceptibility to MS, possibly by modulating resilience to brain white matter damage, or via maturational differences in axonal density or degree of myelination [81].

The third genome-wide significant locus for NODDI (ODI in medial lemniscus) in young adults and replicated in older persons, at chr19q13.12, was previously found to be associated with DTI markers of white matter microstructure in UK Biobank [22, 82]. At this locus, TWAS identified associations of NODDI markers with genetically determined expression levels of several genes (*PROSER3*, *COX6B1*, *UPK1A*, *ZBTB32* and *KMT2B*), all with significant colocalization and in different brain and vascular tissues. Two of these genes, *COX6B1* and *KMT2B*, are involved in monogenic childhood-onset neurological disorders with cognitive decline and delayed motor or cognitive development [83–86]. Interestingly, several transcriptome-wide significant and colocalized genes outside genome-wide significant GWAS loci point to biologically relevant pathways related to neurodevelopment and neurodegeneration (*PICALM*, a known Alzheimer disease gene [47, 87–89], *CAMSAP2*, *AGTPBP1*, *SETD1A*, *EPRS*, *PADI2* and *PSAP*).

Our results support a robust relation between genetic determinants of WMH, the most common imaging feature of cSVD in older adults, and NODDI markers, especially NDI, in young adults. Interestingly, associations of genetically predicted WMH with NDI markers at age 20 were observed primarily in white matter regions most commonly affected by WMH in older age [90, 91], such as the anterior, posterior and superior corona radiata, posterior thalamic radiation, and superior longitudinal fasciculus. This corroborates recent observations that areas where WMH are most likely to appear in older adults are also those with the lowest white matter microstructure integrity on DTI in young adults [91]. In contrast, we found no association of genetically determined blood pressure, the main known risk factor for WMH, with NDI, suggesting that the association between genetically predicted WMH and NDI was likely not mediated by blood pressure. Our results could suggest that NDI, although not specific may be particularly sensitive to variations in the white matter microstructure reflecting a higher propensity to develop WMH, already detectable early in life [17]. Of note, we recently showed significant decrease in NDI values in WMH lesions compared to normal appearing white matter in i-Share participants [88], supporting that NDI is sensitive to microstructural alterations related to WMH. NDI represents the density of neurites relative to extra-neurite volume. Thus, besides neurite density itself, it could also be influenced by changes in components of the extra-neurite volume, e.g. extracellular matrix, known to play a central role in cSVD [9].

Individually, two known WMH risk loci were associated with global NODDI markers after multiple testing correction, in *VCAN* and *NMT1*. While these loci were previously associated with DTI markers in young adults [6], our results provide novel insights into tissue-based mechanisms, showing associations specifically with lower NDI. Additional WMH risk loci were associated with NODDI markers at *P* < 0.05, with NDI and ISOVF at the *SH3PXD2A* locus, previously associated with DTI markers [6], with ISOVF at the *SALL1* locus (a microglial signature gene) [92], and with NDI at the *COL4A1* locus (a gene harboring rare mutations causing monogenic cSVD) [93].

We acknowledge limitations. We did not apply any data harmonization approach to the imaging datasets [94], as we did not combine them; this will be important to consider for future meta-analyses of NODDI GWAS to enhance power for detecting novel associations. NODDI makes certain assumptions that can bias the estimates when they are not met [95, 96]. It assumes a fixed diffusivity for both intra- and extracellular spaces that can cause non-negligible biases in ODI and ISOVF [95]. However, it is one of the few tissue-based models that have been extensively validated histologically [97–99]. Moreover, in secondary analyses we showed that most of the genome-wide significant associations identified with NODDI markers would not have been identified using the standard DTI metrics. Thus, by providing more biologically specific estimates that disambiguate contributions of fiber packing, orientations, and CSF contamination on the diffusion signal, NODDI may offer more sensitive measures of microstructural properties relevant for susceptibility to cSVD than DTI. The cohort of young adults, although unique, was of limited sample size. Further studies in other young cohorts, including in even younger individuals, will be crucial to strengthen our findings and expand them further across the lifespan. The fact that associations of genetic variants for WMH with NODDI metrics in young adults clustered in regions that also harbor the highest frequency of WMH in older age only indirectly supports that NODDI changes may precede WMH in these regions. Ideally, this should be confirmed in the future through a longitudinal design across the lifespan (to our knowledge repeated MRIs in the same individuals from young to older adulthood are currently not available). To explore the relation of NODDI markers with genetically predicted cSVD we used genetic instruments for total WMH volume. In the future, when well powered GWAS of WMH spatial patterns become available, they may allow to better account for the heterogeneity of pathological mechanisms underlying cSVD and provide more granular insights into its lifespan determinants. We cannot exclude bias from postmortem changes in TWAS and analyses of lifetime brain gene expression, as available tissues were mostly from deceased persons [100]. Finally, we used cohorts of predominantly European ancestry and enriched in participants from privileged regions of the world, thus limiting the generalizability of our results. Over 95% of participants in genetic studies on brain MRI traits are of European ancestry and efforts to enhance diversity in this context are of paramount importance [13].

In summary, our study identified novel genetic determinants of NODDI markers of white matter microstructure in young adults. Leveraging this and other resources it provides important novel insights into early-life determinants of cSVD, a leading cause of stroke and dementia. Genetically predicted cSVD burden appears associated with lower neurite density index already at age 20, specifically in regions most likely to harbor cSVD lesions in later life. Genome-wide significant associations with NODDI markers in early adulthood point to genes related to neurodevelopmental, neurodegenerative, and neuroinflammatory processes. Further research is warranted to decipher the molecular pathways and mechanisms involved, as this could open avenues for entirely novel approaches to early prevention.

## Supplementary information

SUPPLEMENTAL MATERIAL

## Data Availability

All data generated during this study are included in this published article and its supplementary information files. The raw datasets for the Rhineland and i-Share studies are not publicly available because of data protection regulations. Specific datasets used for this study can be made available upon reasonable request following the data access rules for the corresponding studies. We used publicly available resources in this manuscript, including data from GTEx (https://gtexportal.org/home/), the Gusev laboratory (http://gusevlab.org/projects/fusion/), the Human Brain Transcriptome project (https://hbatlas.org/), the Betsholtzlab website (https://betsholtzlab.org/VascularSingleCells/database.html), OMIM (https://www.omim.org/); and publicly available GWAS summary statistics for UK Biobank (https://open.win.ox.ac.uk/ukbiobank/big40/), WMH (https://www.ncbi.nlm.nih.gov/gap/, phs002227.v1.p1), blood pressure (https://www.ebi.ac.uk/gwas/, GCST006624,GCST006630,GCST006629), lipid traits (https://csg.sph.umich.edu/willer/public/glgc-lipids2021/), BMI and WHR (https://portals.broadinstitute.org/collaboration/giant/index.php/GIANT_consortium_data_files), type 2 diabetes (https://diagram-consortium.org/index.html) and Alzheimer disease (https://www.ebi.ac.uk/gwas/, GCST90027158).

## Code availability

We used state-of-the-art methods and standard scripts that are publicly available: REGENIE (https://rgcgithub.github.io/regenie/), Plink (https://www.cog-genomics.org/plink/), TWAS-Fusion (http://gusevlab.org/projects/fusion/), GSMR (https://yanglab.westlake.edu.cn/software/gcta/#GSMR), R packages GENESIS (https://bioconductor.org/packages/release/bioc/html/GENESIS.html), RadialMR (https://github.com/WSpiller/RadialMR), TwoSampleMR (https://mrcieu.github.io/TwoSampleMR/).
