# Supplementary material for: Diffusion imaging genomics provides novel insight into early mechanisms of cerebral small vessel disease
Source: Mol Psychiatry. 2024 May 29;29(11):3567–79. doi: 10.1038/s41380-024-02604-7 (PMC11541005; doi:10.1038/s41380-024-02604-7)
Supplement: Supplementary file 1 — SUPPLEMENTAL MATERIAL [file 41380_2024_2604_MOESM1_ESM.pdf]

Diffusion imaging genomics provides novel insight into early mechanisms of cerebral small vessel disease

Supplement

Supplementary methods 2

Study population 2

MRI Acquisition and Phenotyping 2

Cognitive tests in the i-Share study 3

Genotyping, quality control, and imputation 4

Statistical analyses 5

Supplementary references 8

Supplementary Figures 10

Figure S1: Manhattan Plots of GWAS in the i-Share study 11

Figure S2: Regional Plots of (replicated) GWAS signals in the i-Share study 14

Figure S3: Analyses of NODDI genome-wide significant SNPs adjusted for head motion in the i-Share study 15

Figure S4: Sex-stratified analyses of NODDI genome-wide significant SNPs 16

Figure S5: Association of NODDI genome-wide significant SNPs with DTI metrics in the i-Share study 19

Figure S6: Transcriptome-wide association study (TWAS) of NODDI phenotypes in multiple tissues20

Figure S7: Lifetime brain gene expression profile of genes in loci associated with NODDI phenotypes in the i-Share study with a correspondence in the Human Brain Transcriptome database 21

Figure S8: Association of neurovascular traits and vascular risk factors with NODDI metrics in young adults using genetic risk score and Mendelian randomization approaches 24

Figure S9: Association of WMH and vascular risk factors with NODDI metrics in young adults (Mendelian randomization), i-Share study (n=1 758) 25

Figure S10: Correlation of mean WMH frequency for 3C-Dijon participants in each of the 27 JHU regions with Z-scores of association between the WMH GRS and NDI within each JHU region in the i-Share cohort 27

Supplementary tables 28

Table S1: Description of the 28 NODDI regions of interest (ROI) 29

Table S2: Association of lead variants (and proxies) from NODDI markers GWAS in young adults in GWAS of neurovascular traits and vascular risk factors 30

Table S3: Association of lead variants from NODDI markers GWAS in cognitive tests in young adults 31

Table S4: Significant results of transcriptome-wide association study of NODDI for phenotypes presenting replicated genome-wide significant loci in GWAS 32

Table S5: Association of known WMH risk variants and global NODDI markers in young adults (i-Share study, n=1 758) 35

Table S6: Association of WMH and vascular risk factors with NODDI metrics in young adults, i-Share study (n=1 758) 36

Supplementary methods

Study population

The Internet-based Students HeAlth Research Enterprise (i-Share) study is an ongoing prospective population-based cohort of French-speaking students in higher education institutions (HEI) in France (www.i-share.fr), aiming at evaluating students' health and at exploring early mechanisms contributing to the occurrence of common diseases later in life [1]. The bio-Share ancillary study is a biological platform comprising a collection of blood samples from a subset of the i-Share cohort. MRi-Share is a brain imaging ancillary study of i-Share, consisting of a brain MRI and a battery of cognitive tests. Participants were recruited simultaneously for bio-Share and MRi-Share between October 2015 and December 2017, among i-Share participants studying at the University of Bordeaux or other HEIs in Bordeaux and surroundings. Briefly, i-Share participants were eligible for these ancillary studies if they had completed the baseline self-administered online questionnaire, were registered with the national health insurance system, and had signed a written informed consent. To participate in MRi-Share, i-Share students had to be aged between 18 and 35 years and have no contraindication to brain MRI or pregnancy. [2]. The bio-Share and MRi-Share studies were approved by the regional Ethics Committee (Comité de Protection des Personnes Sud-Ouest et Outre-Mer). For the present study, the population comprised i-Share participants taking part in both MRi-Share and bio-Share and for whom brain MRI and genome-wide genotype data passed quality control filters. Here we used the sub-sample of 1 758 MRi-Share and bio-Share participants for whom both brain MRI and genome-wide genotype data were available (mean age: 22.1±2.3 years; 72.2% women).[3]

The Rhineland Study (www.rheinland-studie.de) is an ongoing community-based prospective cohort study that invites inhabitants aged 30 years and above at baseline living in two geographically defined areas in the city of Bonn, Germany. Persons living in those areas were predominantly German with Caucasian ethnicity. The sole exclusion criterion is insufficient German language skills to provide informed consent. Approval to undertake the study was obtained from the ethics committee of the Medical Faculty of the University of Bonn. The study is carried out in accordance with the recommendations of the International Council for Harmonization (ICH) Good Clinical Practice (GCP) standards (ICH-GCP). Written informed consent was obtained from all participants in accordance with the Declaration of Helsinki. We used baseline data of a sub-sample of 724 participants of the Rhineland Study aged 30-40 years with both genotype and MRI scans available that successfully passed dedicated genetics and MRI quality assurance workflows (n=3 917). We excluded participants with neurological disorders including stroke (n=2), multiple sclerosis (n=3) and intracranial hemorrhage (n=5) leading to a sample of 714 participants (mean age: 35.2±3.1 years; 54% women).

MRI Acquisition and Phenotyping

For the i-Share study, MRI acquisitions were performed on a 3 Tesla Siemens Prisma scanner (Siemens Healthcare, Erlangen, Germany) with a 64-channels head coil (gradients: 80 mT/m – 200 T/m/s) at the Bordeaux bio-imaging platform. The MRI protocol lasted about 45 minutes and included a 3D T1-weighted magnetization-prepared rapid gradient-echo (MPRAGE) acquisition at 1 mm isotropic resolution (acquisition time (TA) = 5 min, repetition time (TR)/ echo time (TE)/ inversion time (TI) = 2000/2.0/880 ms, flip angle = 8°, field of view (FOV) = 256 × 256 mm, 192 slices) and multi-band, multi-shell diffusion-weighted MRI (dMRI) scans at 1.75 mm isotropic resolution (TA = 10 min, TR/TE = 3540/75 ms, FOV = 206.5 x 206.5 mm, 84 slices, multiband factor (MB) = 3), with 8 pairs of anterior-posterior (AP) and posterior-anterior (PA) phase-encoded b = 0 s/mm^2, and 8, 32, and 64 directions of b = 300, 1000, and 2000 s/mm^2, respectively, in AP direction.

The Rhineland Study MRI data was acquired at two examination sites in Bonn on 3 Tesla Siemens Prisma MRI scanners (Siemens Healthcare, Erlangen, Germany) equipped with a 64-channel head-neck coils (gradient: 80mT/m – 200 T/m/s). The one-hour Rhineland Study MRI protocol included a 3D T1-weighted multi-echo magnetization-prepared rapid gradient-echo (ME-MPRAGE) sequence at 0.8 mm isotropic resolution (TA = 6.5 min, 4 TE between 1.7 and 6.5 ms, TR/TI = 2560/1100 ms, flip angle = 7°, FOV = 256 × 256 mm, 224 slices). A compressed sensing diffusion spectrum imaging (CS-DSI) protocol was used to collect dMRI scans at 1.5 mm isotropic resolution (TA = 12 min, TR/TE = 5500/105 ms, FOV = 210 x 210 mm, 96 slices, b_{max} = 6800 s/mm^2, Δ = 49.5 ms, δ = 19.7 ms) with 4 pairs of AP and PA phase-encoded b = 0 s/mm^2.[4–6]

For UK Biobank, we used GWAS summary statistics based on MRI phenotypes for which MRI protocols were described in detail elsewhere.[7,8] Briefly, MRI acquisitions were performed on a 3 Tesla Siemens Skyra scanner with a 32-channel head coil (gradients: 80 mT/m – 200 T/m/s). The MRI protocol lasted about 31 minutes and included a 3D T1-weighted MPRAGE, sagittal, R=2, TI/TR=880/2000 ms and multi-band, multi-shell diffusion-weighted MRI (dMRI) scans at 2.0 mm isotropic resolution(TA = 7 min, TR/TE = 3600/92 ms, FOV = 208 x 208 mm, 72 slices, MB = 3), with 3 pairs of AP and PA phase-encoded b = 0 s/mm^2, and 5, 50, 50 directions of b = 0, 1000, and 2000 s/mm^2, respectively, in AP direction.

All cohorts used a similar protocol to generate the NODDI phenotypes. All T1-weighted images were processed using FreeSurfer version 6.0 (http://surfer.nmr.mgh.harvard.edu/) to derive volumetric segmentation and quantitative volumetric measures.[9,10] The estimated total intracranial volume (eTIV) generated by FreeSurfer was used as a proxy for head size. Preprocessing steps for dMRI included the correction of susceptibility-induced and eddy current-induced distortions and head motion using FSL Eddy tool (www.fmrib.ox.ac.uk/fsl) for all cohorts, and for the Rhineland Study, CS reconstruction.[4,11,12] Subsequent estimation of fractional anisotropy (FA) from the diffusion tensor model [13] was performed with Dipy package (0.12.0, http://dipy.org) for iShare and FSL DTIFIT tool for UK Biobank, while neurite density index (NDI), orientation dispersion index (ODI) and isotropic volume fraction (ISOVF) from the NODDI model [14] was performed through voxel-wise model fitting using the AMICO (accelerated microstructure via convex optimization) tool for both i-Share and UK Biobank [15]; for the Rhineland data Study, the microstructure diffusion toolbox (MDT: https://github.com/robbert-harms/MDT) was used to estimate both DTI and NODDI metrics [16]. Subsequently, the standard tract-based spatial statistics (TBSS) framework for FSL was used to project all estimated dMRI parameters to a template FA skeleton in all three cohorts.[17] Image-derived phenotypes were generated by means of the WM skeleton and atlas-based regions of interest (ROIs) for both i-Share and the Rhineland Study. In these cohorts, we calculated the mean across voxels within the WM skeleton and ROI-specific mean values were obtained by means of the labels of well-known white matter tracts in the brain provided by the JHU ICBM DTI 81 atlas[18] available in FSL. The 6 bilateral ROIs of this atlas were selected for the generation of regional phenotypes. For the remaining JHU atlas ROIs, the corresponding labels of the left and right hemisphere were merged before computing the mean values across voxels within the merged label ROIs. Thus, in total, one global and 27 regional markers were generated for each of the NODDI metrics, leading to a total number of 84 NODDI markers. The regions of interest (ROIs) analyzed in this study are summarized in **Table S1**.

Cognitive tests in the i-Share study

We leveraged the results from different cognitive tests used in the i-Share study: the number of correct responses to numerical tests for assessing calculation skills (arithmetics and complex calculation), vocabulary test, auditory verbal learning test (immediate and delayed test), matrices test, the mental rotation test, and the mean correct response times to the Stroop test.

- Arithmetics: Thirty-six multiplication tables (i.e. 4 x 3 = 16) were successively presented on the computer screen for 2.5s (ISI= 0.5s) each. The participant had to verify as fast as possible whether the result was correct or incorrect by clicking on left/right arrows of the keyboard (left arrow = correct; right arrow = incorrect). Half of the trials was incorrect. Accuracy and response times were recorded.
- Complex calculation: Six complex calculations (multiplications, additions and subtractions; i.e, 581 – 126 =) were successively presented for 30 s maximum (ISI= 0.5 s). The participant has to mentally compute the calculation and write down the result using their keyboard. Accuracy and response times were recorded.
- Vocabulary: This web-based test is a shortened version of the one found in the BIL&GIN (Mazoyer et al. 2016), derived from the Binois and Pichot (1956) test. The task consisted in finding the synonym of a noun among 6 possibilities. Eight successive trials were proposed and participants had 30 s maximum to answer each trial.
- Auditory verbal learning: This test evaluating verbal memory was performed on a lab computer on-site during the MRI session. It consisted in listening a series of 15 concrete nouns. Immediately after listening, participants had to recall as many words as possible (immediate recall) by typing them on keyboard. Then, after 20 min corresponding to the completion of another test, participants were asked to type them again (delayed recall).
- Matrices: In this web-based test participants were presented with one matrix made of 9 different visual patterns that were structurally related, with one missing. The participants had to click on the proposal that completed the matrix among 8 possible items. Two matrices were used as a training, 5 others as the testing set. Each matrix was presented for 2 min at maximum.
- Rotation: This web-based test is a shortened version of the Vandenberg and Kuse's test (1978). The participant had to identify the two 3D figures among the 4 proposed that corresponded to the target figure seen from a different angle (as if they had rotated). There were always 2 figures that matched and 2 that did not match the target figure. There were two trials for training, and 5 others were presented on one page for testing. For the testing phase, the five lines are presented for 2 min at maximum. The response is considered as correct when the two correct figures are ticked in each line.
- Stroop test: The participant had to indicate the color of the written word as fast as possible (3s max + ISI 0,5s) by clicking on one the four boxes showing different colors. Trials were either congruent (20 items, word naming the color it was printed in), incongruent (20 items, word naming the color different from the one it was printed in, including 6 negative primes) or neutral (20 items with words naming non-color adjectives, such as 'fort', 'plein', 'grave', 'neuf').

Cognitive variables were transformed using an indirect inverse normal transformation (applying inverse normal transformation to residuals from linear regression of cognition adjusted for covariates [age, sex, and the first four principal components of population stratification]).

Genotyping, quality control, and imputation

Genome-wide genotyping of 1 872 i-Share participants was performed using the Affymetrix Precision Medicine Axiom Array at McGill Genome Center (Canada). After quality control, genotype data were available for 1 862 participants (7 participants were removed due to sex discrepancies, 2 participants who appeared to be duplicates but not twins, and one participant with a kinship coefficient >0.0625 (third degree related) with more than 20 other participants, suggesting a possible sample contamination (KING software)).[19] After applying standard quality control procedures (SNP call rate

<98%, Hardy-Weinberg Equilibrium p<0.001), we imputed the genotypes on the Haplotype Reference Consortium (HRC) reference panel.[3]

In the Rhineland study, DNA extracted from buffy coat samples were genotyped using Infinium Omni2.5Exome-8 BeadChip containing 2 612 357 SNPs and processed using GenomeStudio (version 2.0.5). Quality control of genotypes was performed using PLINK (version 1.9). Single-nucleotide polymorphisms (SNPs) exclusion criteria were Hardy-Weinberg disequilibrium ($p<1\times10^{-6}$), minor allele frequency (<0.01) and poor genotyping rate (<99%). Participants with potentially problematic samples were excluded, comprising cases with poor call rate (<95%), abnormal heterozygosity and gender mismatch. Since variation in population structure can cause systematic differences in allele frequencies[20], we used EIGENSTRAT (version 16000), which uses principal components (PCs) to detect and correct for variation in population structure.[20] Based on the EIGENSTRAT estimation, we excluded participants of non-Caucasian descent, retaining only participants from Caucasian descent for further analysis. We used the 1000 Genomes phase 3 reference panel [21] version 5 for the imputation of missing genotypes using impute2 (version 2).[22]

Statistical analyses

Genome-wide association study (GWAS)

We performed 84 GWAS using the genome-wide linear mixed model implemented in REGENIE.[23] REGENIE computation is composed of two steps. The step 1 uses a subset of genetic markers to fit a whole genome regression model that captures a good fraction of the phenotype variance attributable to genetic effects. We used a pruned subset of genotyped SNPs using a LD-r^2 threshold of 0.9 with a window size of 1 000 and a step size of 100 markers. The step 2 uses a larger set of genetic markers and tests them for association with the phenotype conditional upon the prediction from the regression model in Step 1, using a leave one chromosome out (LOCO) scheme.[23] These analyses were restricted to Single Nucleotide Polymorphisms (SNPs) with imputation score >0.5 and MAF>0.01 and adjusted for age at MRI, sex, intracranial volume and the first four principal components of population stratification. We finally annotated the nearest genes and SNPs functions using Annovar.[24]

In the previously published UK Biobank GWAS, NODDI markers were quantile normalized and confounds were removed from the data prior to analyses (including the 40 population genetic principal components and confounds for age, head size, sex, head motion during functional MRI, scanner table position, imaging center and scan date-related slow drifts).[8] Participants without recent UK ancestry and related participants were removed from the analyses. Association analyses were then performed using a linear regression model implemented in bgenie.[8]

Transcriptome-wide association study on the genome-wide significant loci in young adults

We performed transcriptome-wide association studies (TWAS) using TWAS-Fusion[25] to identify genes whose expression is significantly associated with NODDI phenotypes. For this we focused only on NODDI phenotypes with genome-wide significant, replicated GWAS loci. Moreover, we restricted the analysis to tissues considered relevant for cerebrovascular disease, and used precomputed functional weights from publicly available gene expression reference panels (expression quantitative trait loci [eQTL] reference panels) from blood, arterial, heart, adipose, nerve and brain tissues from the Genotype-Tissue Expression version 8 (GTEx v8)[26] database, as well as cross-tissue weights generated in GTEx v8 using sparse canonical correlation analysis (sCCA).[27] TWAS-Fusion was then used to estimate the TWAS association statistics between predicted expression and each NODDI phenotype by integrating information from expression reference panels (SNP-expression weights), GWAS summary statistics (SNP-trait effect estimates), and LD reference panels (SNP correlation matrix).[25] Transcriptome-wide significance at $p<7.8\times10^{-6}$ was based on the average number of

features (6 400 genes) tested across all the reference panels, but we also considered suggestive associations with p<1x10-4. These genes were then tested in conditional analyses in TWAS-Fusion.[25] Next, we performed a colocalization analysis (COLOC) on the conditionally significant genes (p<0.05) to estimate the posterior probability of a shared causal variant between the gene expression and trait association (PP4).[28] We used a prior probability of $p<1x10^{-4}$ for the NODDI association. Genes presenting a PP4≥0.75 were considered as colocalized. Colocalized genes for which eQTLs reached genome-wide significance in association with NODDI phenotypes, or were in moderate-high LD (LD-r^2>0.5) with any of the lead SNPs of genome-wide significant loci for the corresponding trait, were considered as in a GWAS locus.

Association of WMH and vascular risk factors with NODDI phenotypes in young adults

Genetic risk scores

In order to study the association of genetically determined WMH and vascular risk factors with the NODDI markers in i-Share, we generated weighted GRS from the largest latest GWAS summary statistics on individuals of European ancestry for traits used in the lookup of genome-wide significant SNPs: blood pressure,[29] lipids,[30] BMI,[31] WHR,[31] type 2 diabetes,[32] and WMH.[33] We generated GRS for these traits by summing up the number of independent risk alleles identified as genome-wide significant ($p<5.0\times10^{-8}$) in the largest published GWAS of each trait weighting each risk allele by the regression coefficient for the corresponding SNP in the published GWAS ($GRS = \sum_{i=1}^{m} A_i\beta_i$, where *i* is a SNP among m independent SNPs, *A* is the risk allele for SNP *i* and *β* is the regression coefficient in the published GWAS). SNPs were clumped using Plink 1.9 (parameters: LD-r^2>0.01 and distance<1Mb). Only SNPs with an imputation score>0.9 and a MAF>0.01 were included in GRS calculations. To compare the effect size between the different GRS, we standardized the scores by applying the following formula: $Z = \frac{X-\bar{X}}{\sigma}$ where Z is the standardized GRS, X the untransformed GRS, $\bar{X}$ is the mean of X and σ the standard deviation of X. All associations were tested using linear mixed models in R v3.6.1 (package GENESIS)[34] using a genetic relationship matrix and adjusted for age, sex, total intracranial volume, and the first four principal components of population stratification.

Mendelian randomization

In order to confirm the significant associations observed in the GRS analyses and infer the putative effect of age-related neurovascular traits and vascular risk factors on the NODDI phenotypes in young adults, we performed a two-sample MR analysis. We used three distinct two-sample MR approaches to strengthen the validity of our findings: RadialMR[35], TwoSampleMR[36] and Generalised Summary-data-based MR (GSMR)[37].

To build our instruments for MR, we used genetic risk variants for exposure traits: SBP, DBP, PP, LDL, HDL, TG, BMI, WHR, WMH. Only independent SNPs (LD-r^2<0.01 for RadialMR and TwoSampleMR, and LD-r^2<0.05 for GSMR) reaching genome-wide significance ($p<5.10^{-8}$) were included.[38] To select these SNPs, we clumped the summary statistics of the exposures (window: 1 000kb, r^2<0.01) after filtering the SNPs, excluding variants with MAF<0.01, ambiguous alleles, and non-matching alleles between the exposure and i-Share summary statistics and variants with an imputation score<0.9 in i-Share.

In RadialMR,[35] the putative causal effect of an exposure on the outcome was estimated using the fixed-effect inverse-variance weighting (IVW) method.[35] The first step was to calculate the ratio estimate for the causal effect of exposure on outcome for each SNP included in the analyses. Then, the overall IVW estimate was calculated using the modified-second order inverse variance weight of the ratio estimate.[35,39] Cochran's Q statistic was used to test for the presence of heterogeneity (p<0.05) due to horizontal pleiotropy that occurs when instruments affect the outcome independently of the

exposure.[35] Outlier SNPs were identified by regressing the predicted causal estimate against the inverse variance weights. After excluding these SNPs, we ran another set of analyses composed of another IVW test and a MR-Egger regression.[40] In the Egger regression, the heterogeneity was assessed using Rücker's Q' statistic.[35] We formally ruled out horizontal pleiotropy when the MR-Egger intercept was not significant ($p \geq 0.05$) after removing outlier SNPs.

In TwoSampleMR,[36] we harmonized data between exposures and outcomes using the default parameters and tested the same associations as in RadialMR with the weighted median, random-effect IVW and MR-Egger methods in a purpose of confirmation of the observed effects.

Finally, we used GSMR (implemented in GCTA),[37] after removing SNPs that have pleiotropic effects on both exposure and outcome, so not satisfying the underlying assumptions for valid instruments. We used the HEIDI-outlier method to remove such SNPs (pHEIDI<0.01) and ran GSMR, based on two-step least squares approach, to estimate the effect of the exposures on the NODDI markers.[37]

Supplementary references

1. Montagni I, Guichard E, Kurth T. Association of screen time with self-perceived attention problems and hyperactivity levels in French students: a cross-sectional study. BMJ Open. 2016;6:e009089.
2. Tsuchida A, Laurent A, Crivello F, Petit L, Joliot M, Pepe A, et al. The MRi-Share database: brain imaging in a cross-sectional cohort of 1870 university students. Brain Struct Funct. 2021;226:2057–2085.
3. Le Grand Q, Satizabal CL, Sargurupremraj M, Mishra A, Soumaré A, Laurent A, et al. Genomic Studies Across the Lifespan Point to Early Mechanisms Determining Subcortical Volumes. Biol Psychiatry Cogn Neurosci Neuroimaging. 2022;7:616–628.
4. Tobisch A, Stirnberg R, Harms RL, Schultz T, Roebroeck A, Breteler MMB, et al. Compressed Sensing Diffusion Spectrum Imaging for Accelerated Diffusion Microstructure MRI in Long-Term Population Imaging. Front Neurosci. 2018;12:650.
5. Menzel MI, Tan ET, Khare K, Sperl JI, King KF, Tao X, et al. Accelerated diffusion spectrum imaging in the human brain using compressed sensing. Magn Reson Med. 2011;66:1226–1233.
6. Wedeen VJ, Hagmann P, Tseng W-YI, Reese TG, Weisskoff RM. Mapping complex tissue architecture with diffusion spectrum magnetic resonance imaging. Magn Reson Med. 2005;54:1377–1386.
7. Miller KL, Alfaro-Almagro F, Bangerter NK, Thomas DL, Yacoub E, Xu J, et al. Multimodal population brain imaging in the UK Biobank prospective epidemiological study. Nat Neurosci. 2016;19:1523–1536.
8. Smith SM, Douaud G, Chen W, Hanayik T, Alfaro-Almagro F, Sharp K, et al. An expanded set of genome-wide association studies of brain imaging phenotypes in UK Biobank. Nat Neurosci. 2021;24:737–745.
9. Fischl B, Salat DH, Busa E, Albert M, Dieterich M, Haselgrove C, et al. Whole Brain Segmentation. Neuron. 2002;33:341–355.
10. Fischl B. FreeSurfer. NeuroImage. 2012;62:774–781.
11. Andersson JLR, Skare S, Ashburner J. How to correct susceptibility distortions in spin-echo echo-planar images: application to diffusion tensor imaging. NeuroImage. 2003;20:870–888.
12. Tobisch A, Schultz T, Stirnberg R, Varela-Mattatall G, Knutsson H, Irarrázaval P, et al. Comparison of basis functions and q-space sampling schemes for robust compressed sensing reconstruction accelerating diffusion spectrum imaging. NMR Biomed. 2019;32:e4055.
13. Basser PJ, Mattiello J, LeBihan D. MR diffusion tensor spectroscopy and imaging. Biophys J. 1994;66:259–267.
14. Zhang H, Schneider T, Wheeler-Kingshott CA, Alexander DC. NODDI: practical in vivo neurite orientation dispersion and density imaging of the human brain. NeuroImage. 2012;61:1000–1016.
15. Daducci A, Canales-Rodríguez EJ, Zhang H, Dyrby TB, Alexander DC, Thiran J-P. Accelerated Microstructure Imaging via Convex Optimization (AMICO) from diffusion MRI data. NeuroImage. 2015;105:32–44.
16. Harms RL, Fritz FJ, Tobisch A, Goebel R, Roebroeck A. Robust and fast nonlinear optimization of diffusion MRI microstructure models. NeuroImage. 2017;155:82–96.
17. Smith SM, Jenkinson M, Johansen-Berg H, Rueckert D, Nichols TE, Mackay CE, et al. Tract-based spatial statistics: voxelwise analysis of multi-subject diffusion data. NeuroImage. 2006;31:1487–1505.
18. Mori S, Oishi K, Jiang H, Jiang L, Li X, Akhter K, et al. Stereotaxic white matter atlas based on diffusion tensor imaging in an ICBM template. NeuroImage. 2008;40:570–582.
19. Manichaikul A, Mychaleckyj JC, Rich SS, Daly K, Sale M, Chen W-M. Robust relationship inference in genome-wide association studies. Bioinformatics. 2010;26:2867–2873.

20. Price AL, Patterson NJ, Plenge RM, Weinblatt ME, Shadick NA, Reich D. Principal components analysis corrects for stratification in genome-wide association studies. Nat Genet. 2006;38:904–909.
21. 1000 Genomes Project Consortium, Auton A, Brooks LD, Durbin RM, Garrison EP, Kang HM, et al. A global reference for human genetic variation. Nature. 2015;526:68–74.
22. Howie BN, Donnelly P, Marchini J. A flexible and accurate genotype imputation method for the next generation of genome-wide association studies. PLoS Genet. 2009;5:e1000529.
23. Mbatchou J, Barnard L, Backman J, Marcketta A, Kosmicki JA, Ziyatdinov A, et al. Computationally efficient whole-genome regression for quantitative and binary traits. Nat Genet. 2021;53:1097–1103.
24. Wang K, Li M, Hakonarson H. ANNOVAR: functional annotation of genetic variants from high-throughput sequencing data. Nucleic Acids Res. 2010;38:e164–e164.
25. Gusev A, Ko A, Shi H, Bhatia G, Chung W, Penninx BWJH, et al. Integrative approaches for large-scale transcriptome-wide association studies. Nat Genet. 2016;48:245–252.
26. GTEx Consortium. The GTEx Consortium atlas of genetic regulatory effects across human tissues. Science. 2020;369:1318–1330.
27. Feng H, Mancuso N, Gusev A, Majumdar A, Major M, Pasaniuc B, et al. Leveraging expression from multiple tissues using sparse canonical correlation analysis and aggregate tests improves the power of transcriptome-wide association studies. PLOS Genet. 2021;17:e1008973.
28. Giambartolomei C, Vukcevic D, Schadt EE, Franke L, Hingorani AD, Wallace C, et al. Bayesian Test for Colocalisation between Pairs of Genetic Association Studies Using Summary Statistics. PLoS Genet. 2014;10:e1004383.
29. Evangelou E, Warren HR, Mosen-Ansorena D, Mifsud B, Pazoki R, Gao H, et al. Genetic analysis of over one million people identifies 535 new loci associated with blood pressure traits. Nat Genet. 2018;50:1412–1425.
30. Graham SE, Clarke SL, Wu K-HH, Kanoni S, Zajac GJM, Ramdas S, et al. The power of genetic diversity in genome-wide association studies of lipids. Nature. 2021;600:675–679.
31. Pulit SL, Stoneman C, Morris AP, Wood AR, Glastonbury CA, Tyrrell J, et al. Meta-analysis of genome-wide association studies for body fat distribution in 694 649 individuals of European ancestry. Hum Mol Genet. 2019;28:166–174.
32. Mahajan A, Taliun D, Thurner M, Robertson NR, Torres JM, Rayner NW, et al. Fine-mapping type 2 diabetes loci to single-variant resolution using high-density imputation and islet-specific epigenome maps. Nat Genet. 2018;50:1505–1513.
33. Sargurupremraj M, Suzuki H, Jian X, Sarnowski C, Evans TE, Bis JC, et al. Cerebral small vessel disease genomics and its implications across the lifespan. Nat Commun. 2020;11:6285.
34. Gogarten SM, Sofer T, Chen H, Yu C, Brody JA, Thornton TA, et al. Genetic association testing using the GENESIS R/Bioconductor package. Bioinforma Oxf Engl. 2019;35:5346–5348.
35. Bowden J, Spiller W, Del Greco M F, Sheehan N, Thompson J, Minelli C, et al. Improving the visualization, interpretation and analysis of two-sample summary data Mendelian randomization via the Radial plot and Radial regression. Int J Epidemiol. 2018;47:1264–1278.
36. Hemani G, Zheng J, Elsworth B, Wade KH, Haberland V, Baird D, et al. The MR-Base platform supports systematic causal inference across the human phenome. eLife. 2018;7:e34408.
37. Zhu Z, Zheng Z, Zhang F, Wu Y, Trzaskowski M, Maier R, et al. Causal associations between risk factors and common diseases inferred from GWAS summary data. Nat Commun. 2018;9:224.
38. Swerdlow DI, Kuchenbaecker KB, Shah S, Sofat R, Holmes MV, White J, et al. Selecting instruments for Mendelian randomization in the wake of genome-wide association studies. Int J Epidemiol. 2016;45:1600–1616.
39. Bowden J, Del Greco M F, Minelli C, Zhao Q, Lawlor DA, Sheehan NA, et al. Improving the accuracy of two-sample summary-data Mendelian randomization: moving beyond the NOME assumption. Int J Epidemiol. 2019;48:728–742.
40. Bowden J, Davey Smith G, Burgess S. Mendelian randomization with invalid instruments: effect estimation and bias detection through Egger regression. Int J Epidemiol. 2015;44:512–525.

Supplementary Figures

Figure S1: Manhattan Plots of GWAS in the i-Share study

Figure S1A: NDI phenotypes

CWM

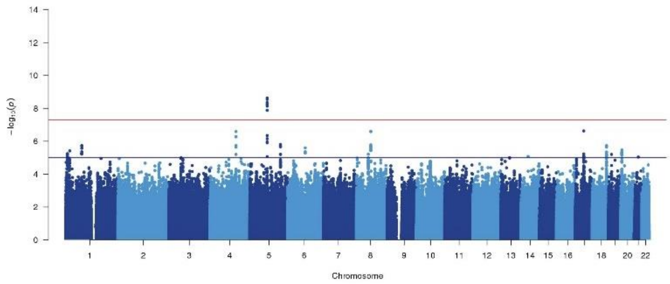

FX.ST

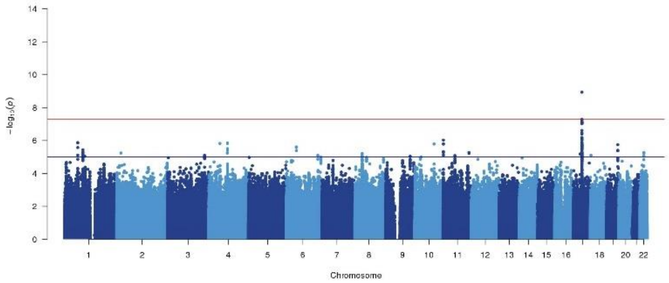

PCR

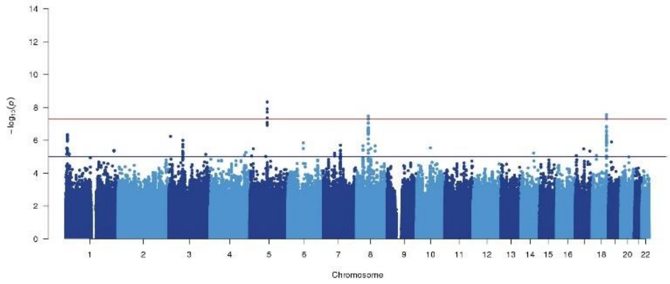

PTR

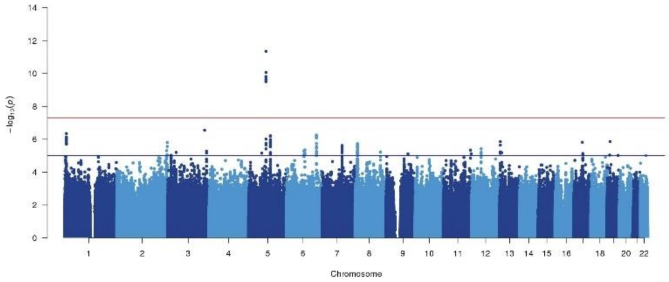

RLIC

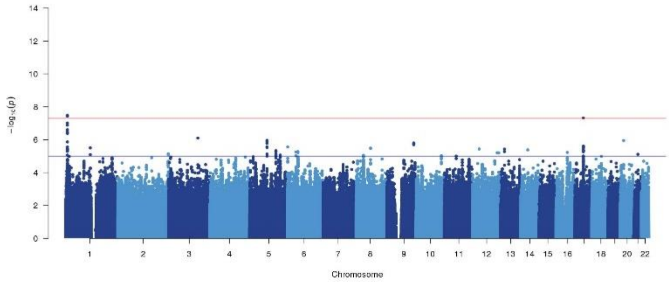

SLF

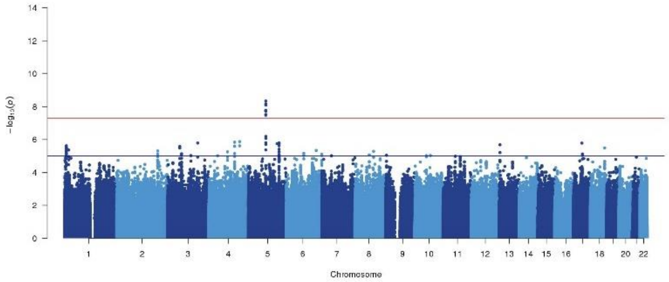

SS

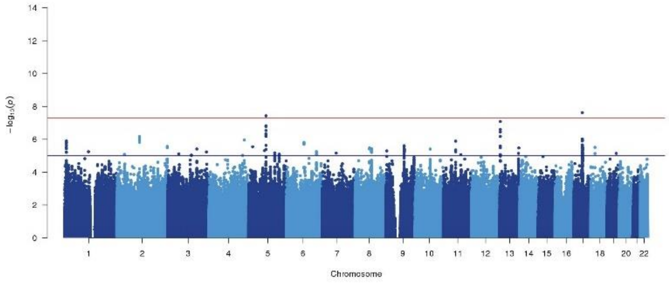

Figure S1B: ODI phenotypes

CWM

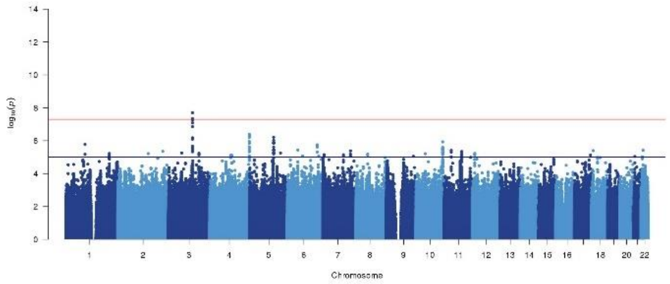

ACR

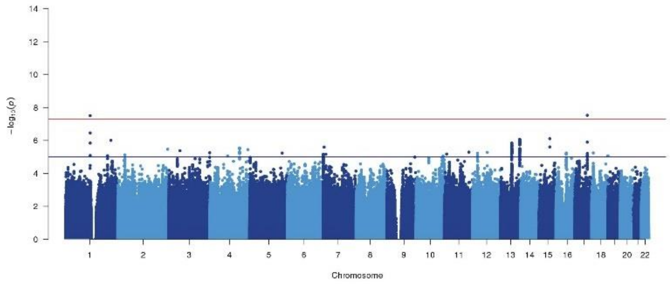

BCC

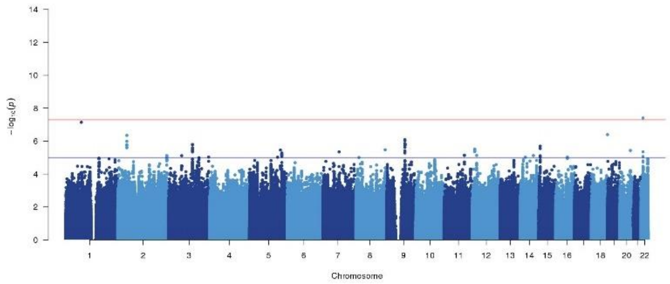

CgC

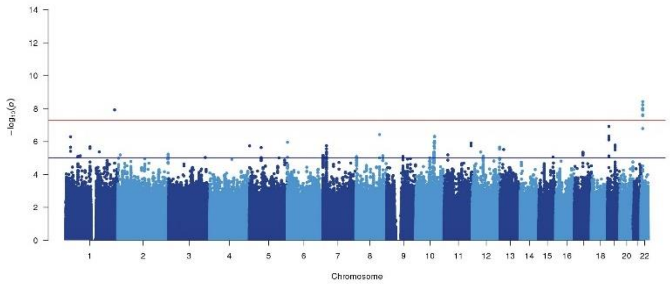

ML

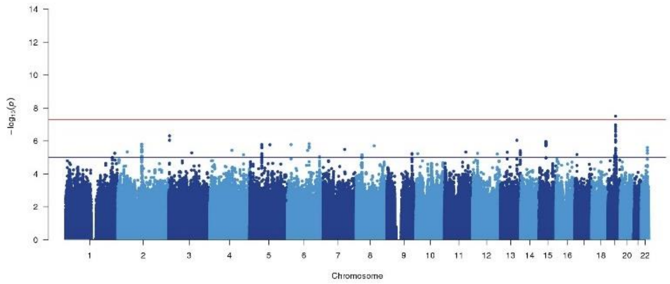

SFO

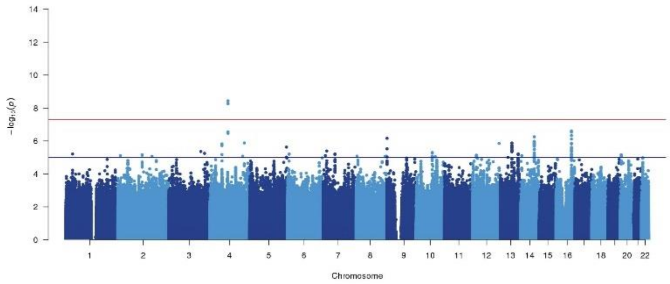

SS

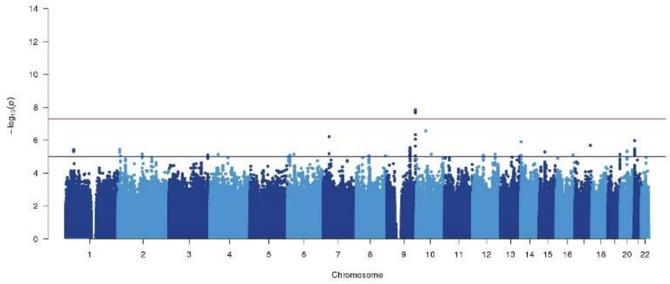

Figure S1C: ISOVF phenotypes

CWM

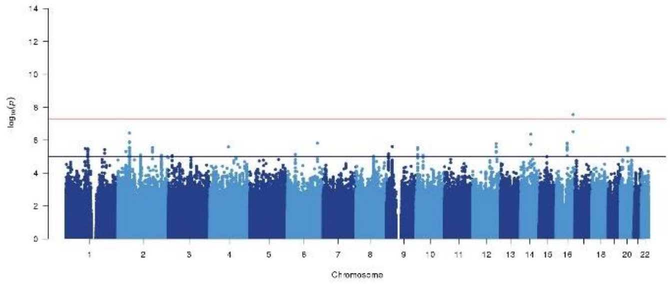

CgC

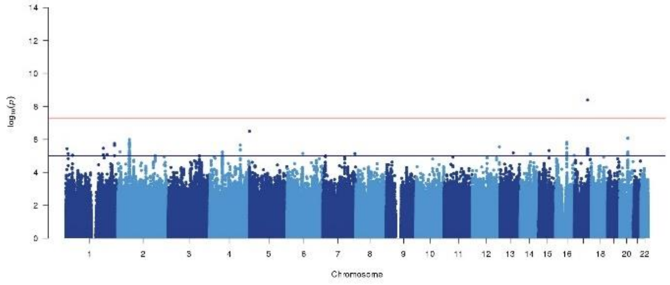

EC

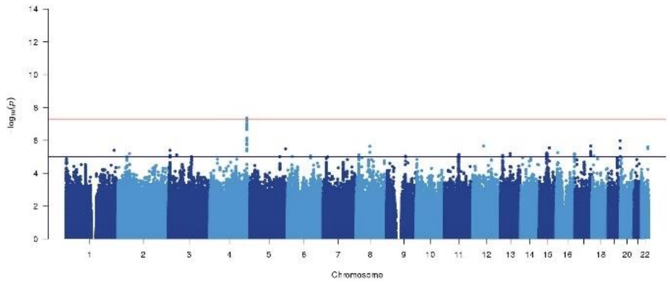

MCP

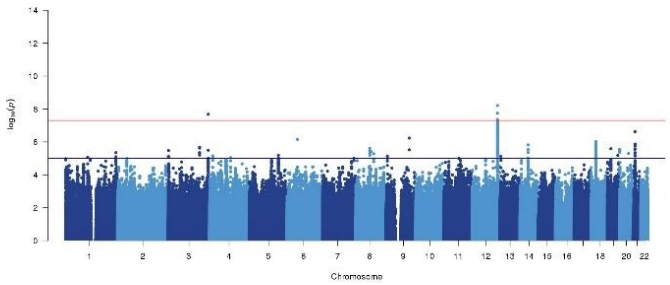

PCR

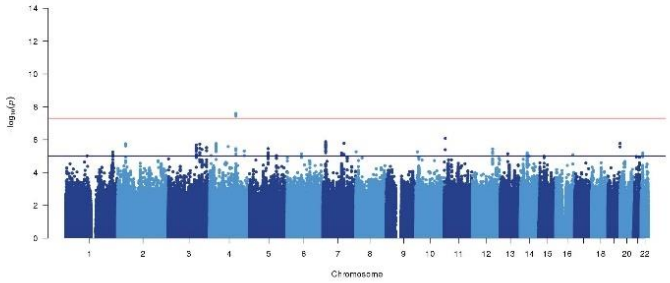

UNC

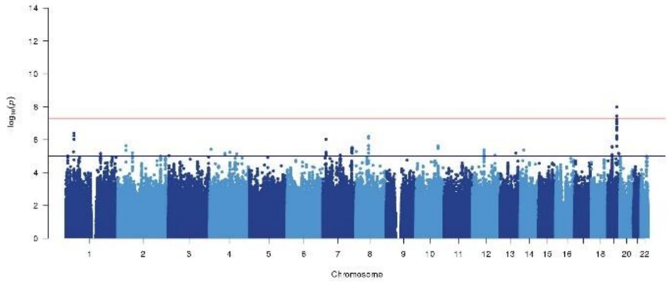

NDI: Neurite Density Index; ODI: Orientation Dispersion Index; ISOVF: Isotropic Volume Fraction

Figure S2: Regional Plots of (replicated) GWAS signals in the i-Share study

rs10052710 (5q14.3 - *VCAN*) - NDI-CWM

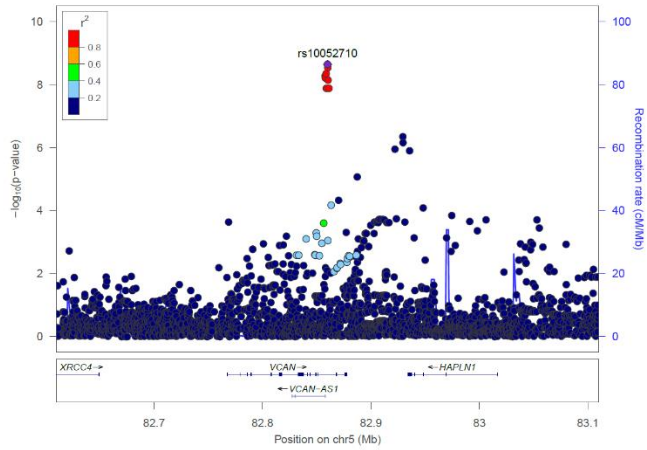

rs13176921 (5q14.3 - *VCAN*) – NDI-PCR

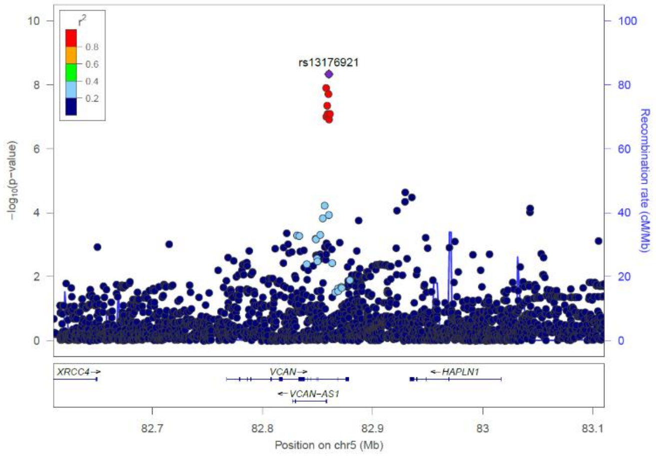

rs13176921 (5q14.3 - *VCAN*) – NDI-PTR

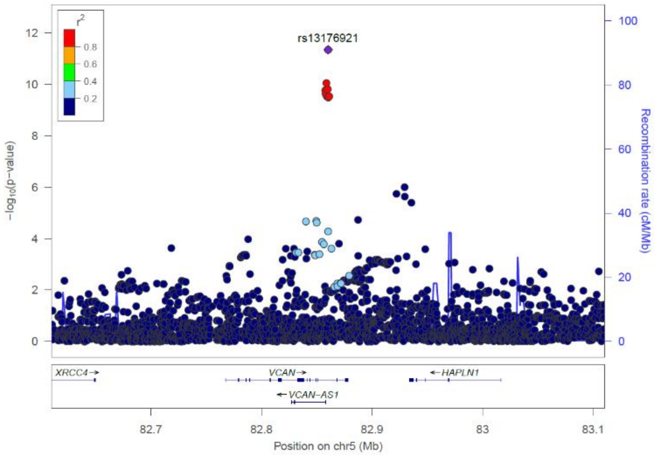

rs13176921 (5q14.3 - *VCAN*) – NDI-SLF

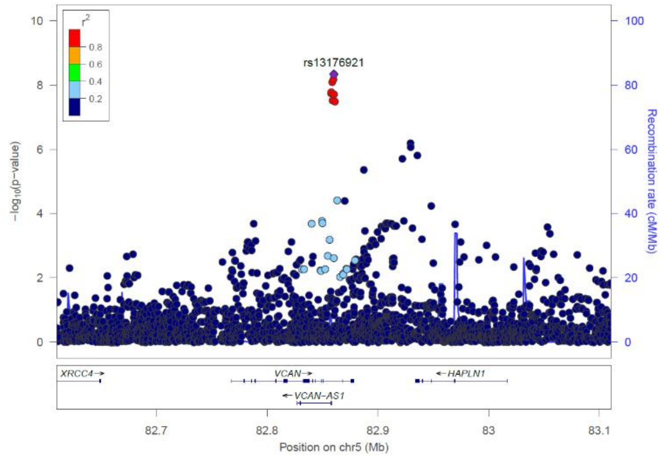

rs13176921 (5q14.3 - *VCAN*) – NDI-SS

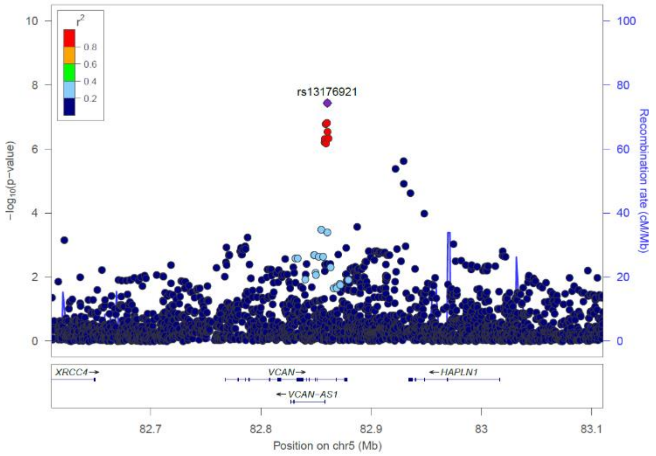

rs1053004 (17q21.2 - *STAT3*) - NDI-FX.ST

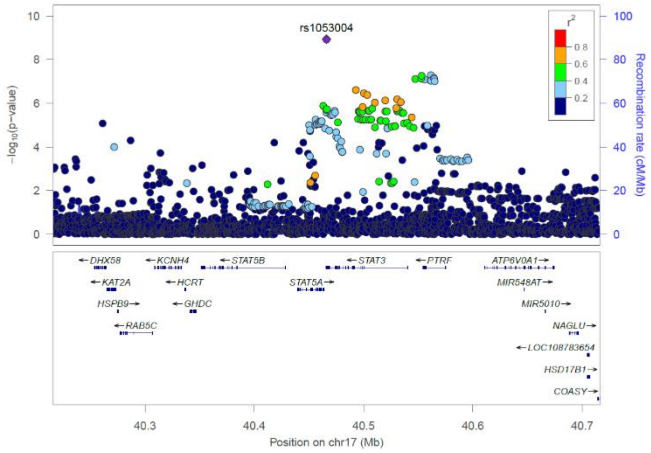

rs1053004 (17q21.2 - *STAT3*) – NDI-SS

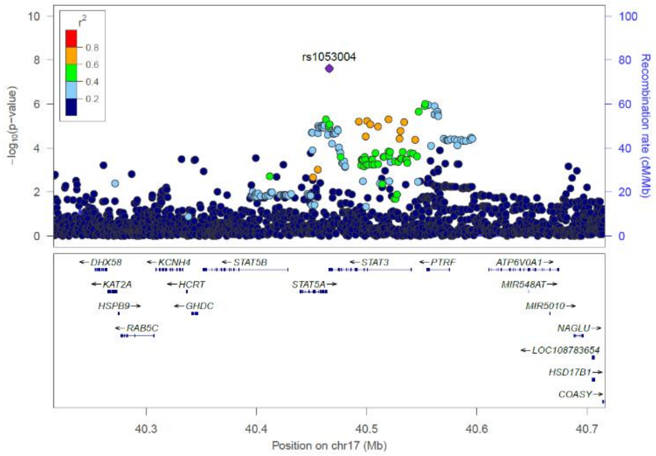

rs807478 (19q13.12 - *PROSER3*) – ODI-ML

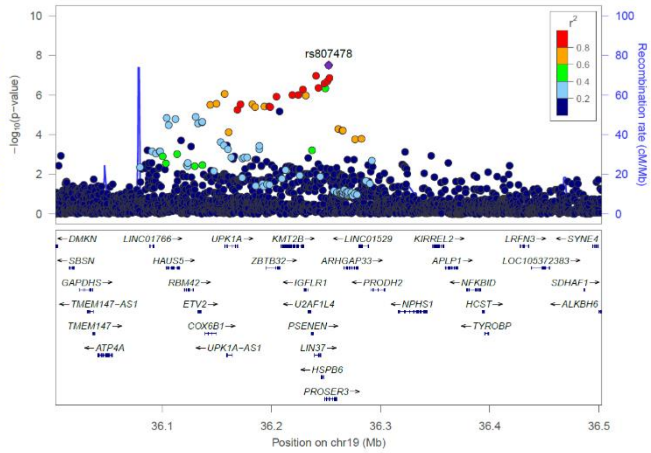

NDI: Neurite Density Index; ODI: Orientation Dispersion Index; ISOVF: Isotropic Volume Fraction

Figure S3: Analyses of NODDI genome-wide significant SNPs adjusted for head motion in the i-Share study

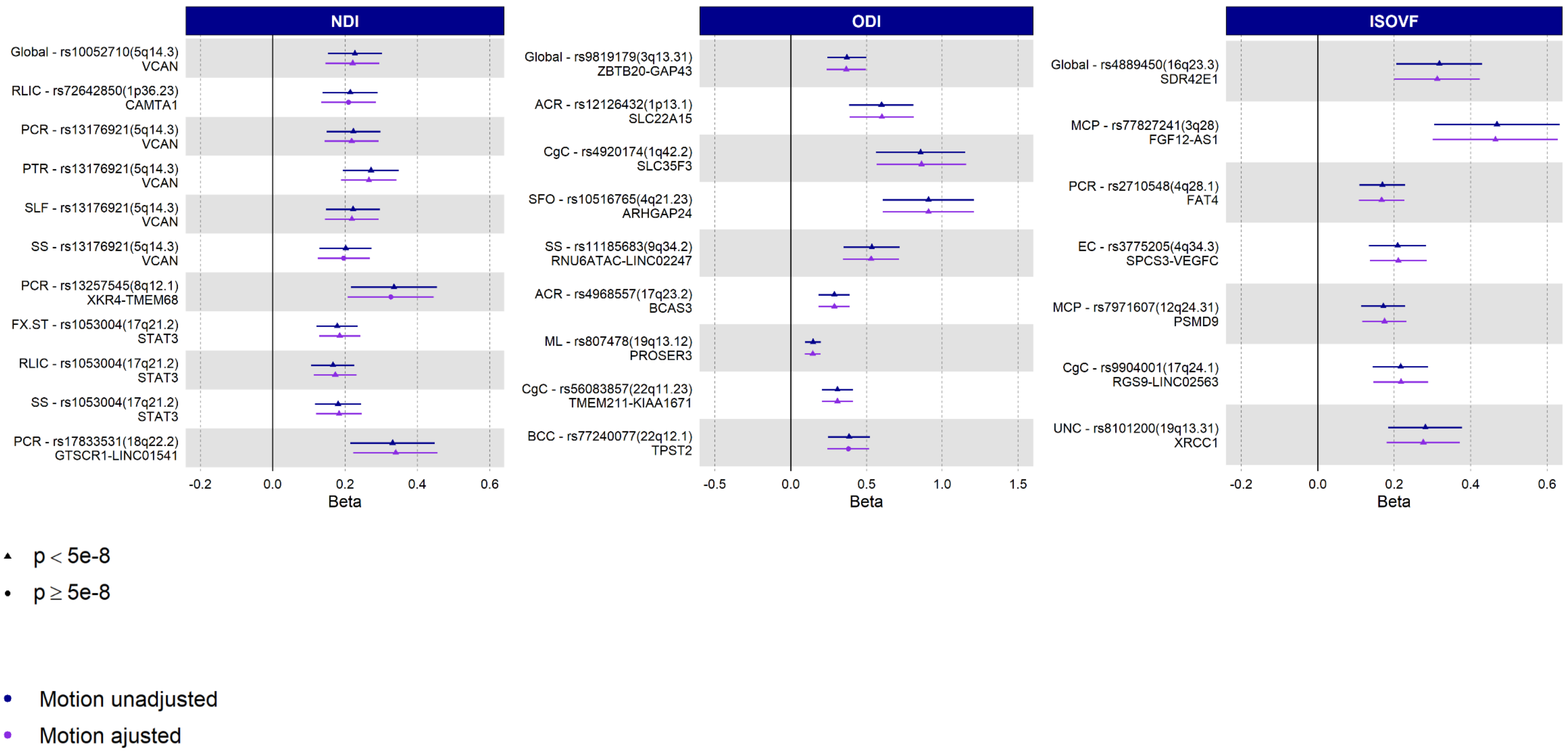

ACR: Anterior corona radiata; BCC: Body corpus callosum; CgC: Cingulum cingulate gyrus; EC: External capsule; FX.ST: Fornix cres or stria terminalis; Global: global metrics; MCP: Middle cerebellar peduncle; ML: Medial lemniscus; PCR: Posterior corona radiata; PTR: Posterior thalamic radiation; RLIC: Retrolenticular part of the internal capsule; SFO: Superior fronto-occipital fasciculus; SLF: Superior longitudinal fasciculus; SS: Sagittal stratum; UNC: Uncinate fasciculus. NDI: Neurite Density Index; ODI: Orientation Dispersion Index; ISOVF: Isotropic Volume Fraction.

Figure S4: Sex-stratified analyses of NODDI genome-wide significant SNPs

Figure S4A: i-Share study

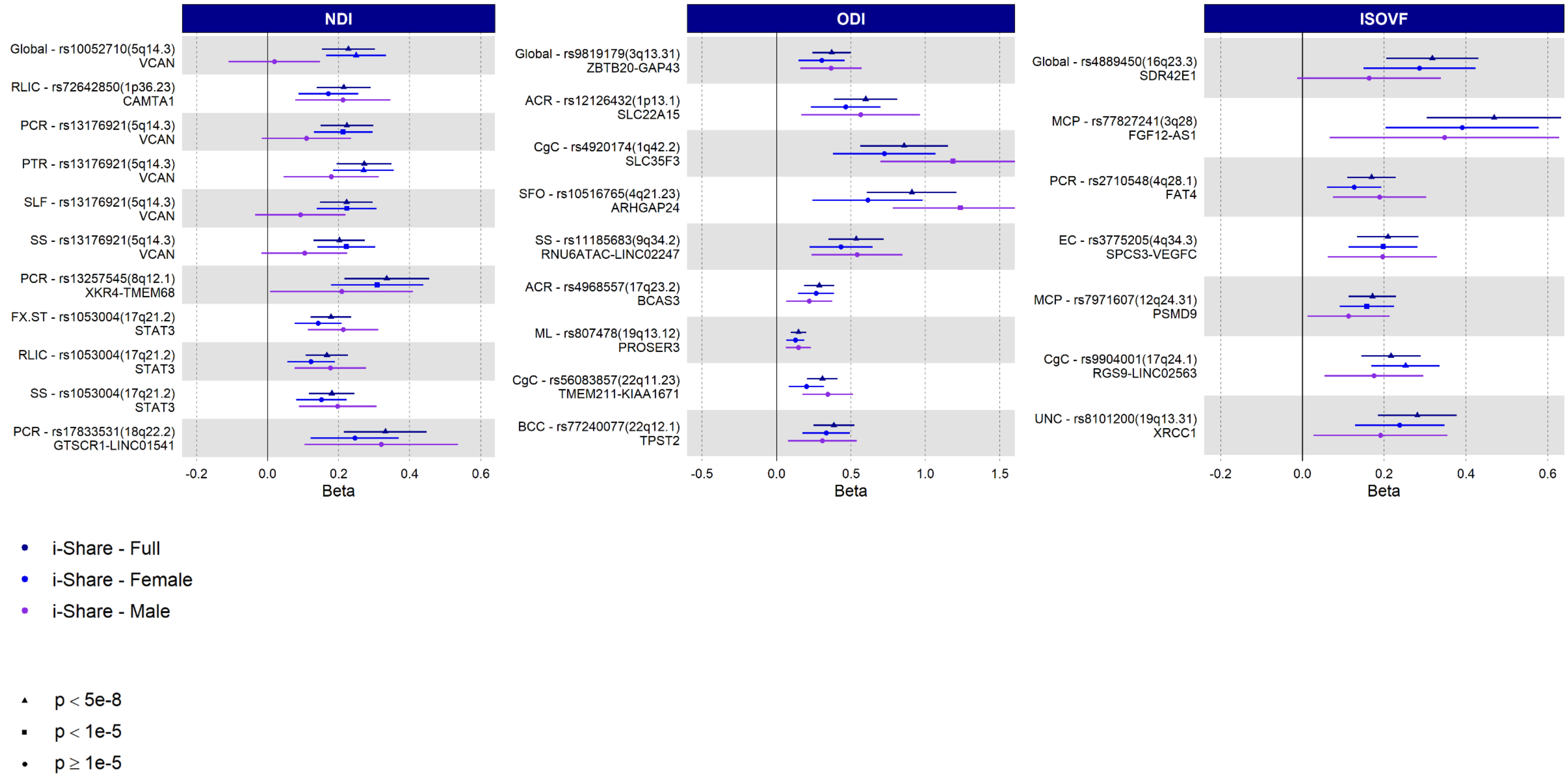

ACR: Anterior corona radiata; BCC: Body corpus callosum; CgC: Cingulum cingulate gyrus; EC: External capsule; FX.ST: Fornix cres or stria terminalis; Global: global metrics; MCP: Middle cerebellar peduncle; ML: Medial lemniscus; PCR: Posterior corona radiata; PTR: Posterior thalamic radiation; RLIC: Retrolenticular part of the internal capsule; SFO: Superior fronto-occipital fasciculus; SLF: Superior longitudinal fasciculus; SS: Sagittal stratum; UNC: Uncinate fasciculus. NDI: Neurite Density Index; ODI: Orientation Dispersion Index; ISOVF: Isotropic Volume Fraction. “i-Share – Full”: Full sample (N=1 758); “i-Share – Female”: Women only (N=1 269); “i-Share – Male”: Men only (N=489).

Figure S4B: The Rhineland Study

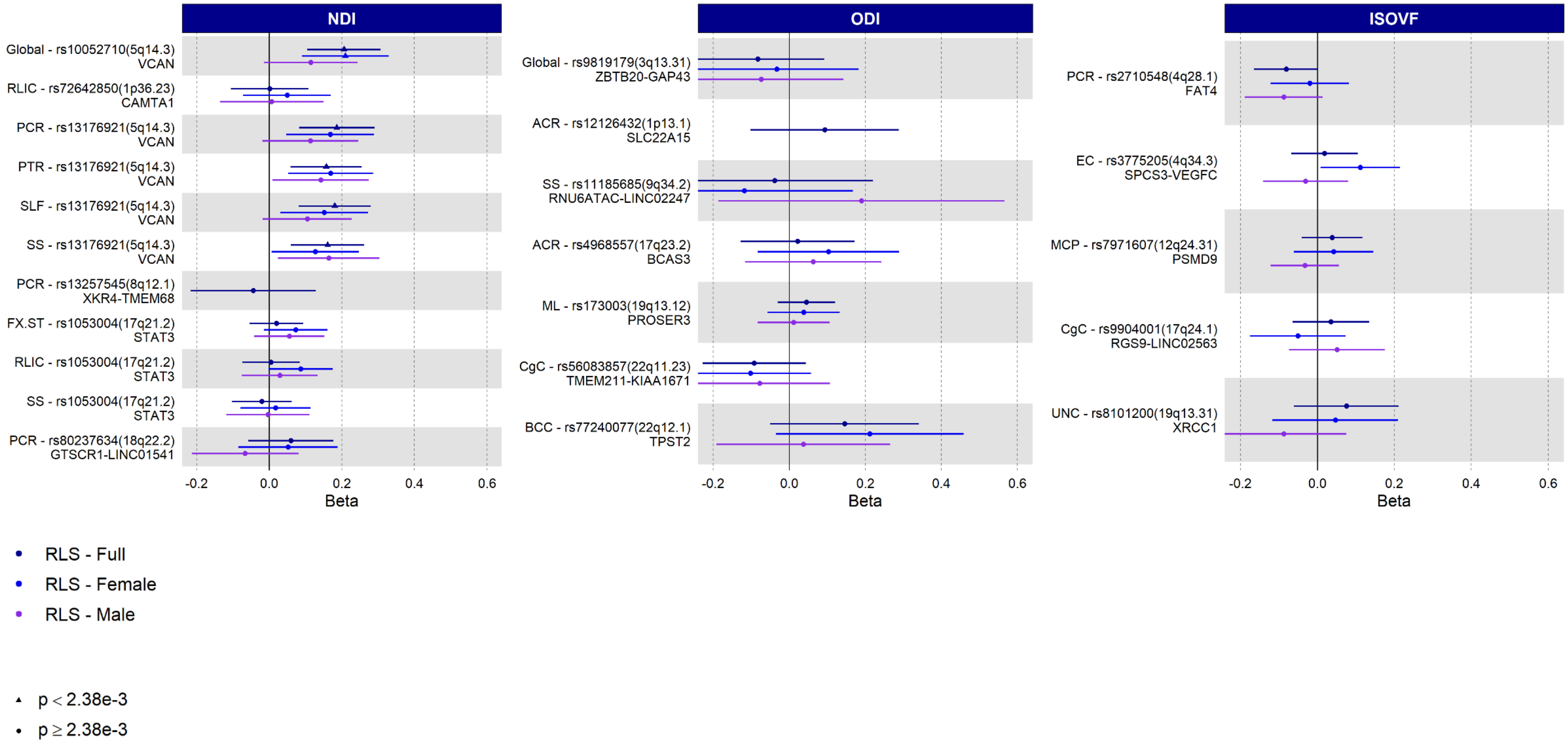

ACR: Anterior corona radiata; BCC: Body corpus callosum; CgC: Cingulum cingulate gyrus; EC: External capsule; FX.ST: Fornix cres or stria terminalis; Global: global metrics; MCP: Middle cerebellar peduncle; ML: Medial lemniscus; PCR: Posterior corona radiata; PTR: Posterior thalamic radiation; RLIC: Retrolenticular part of the internal capsule; SFO: Superior fronto-occipital fasciculus; SLF: Superior longitudinal fasciculus; SS: Sagittal stratum; UNC: Uncinate fasciculus. NDI: Neurite Density Index; ODI: Orientation Dispersion Index; ISOVF: Isotropic Volume Fraction. “RLS – Full”: Full sample (N=714); “RLS – Female”: Women only (N=385); “RLS – Male”: Men only (N=329).

Figure S4C: UK Biobank

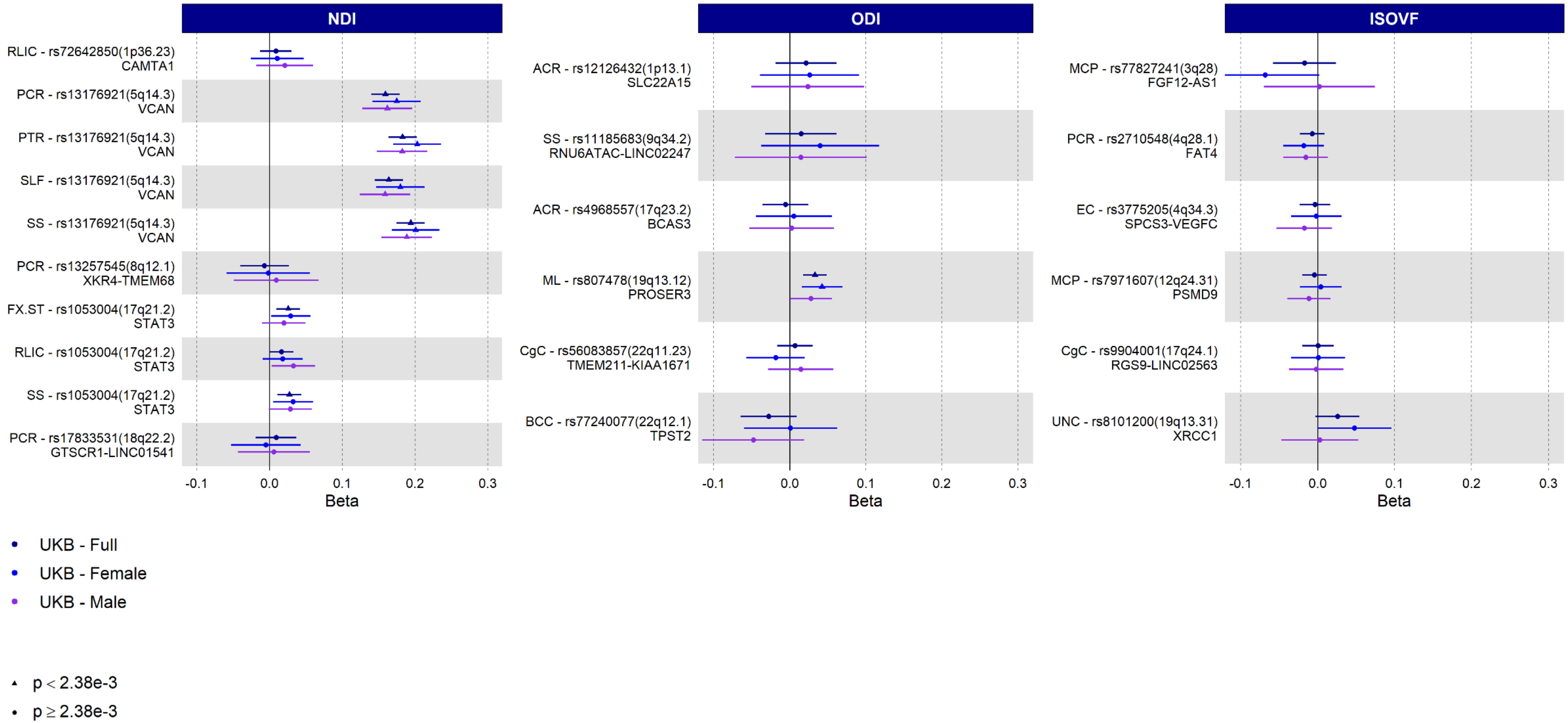

ACR: Anterior corona radiata; BCC: Body corpus callosum; CgC: Cingulum cingulate gyrus; EC: External capsule; FX.ST: Fornix cres or stria terminalis; Global: global metrics; MCP: Middle cerebellar peduncle; ML: Medial lemniscus; PCR: Posterior corona radiata; PTR: Posterior thalamic radiation; RLIC: Retrolenticular part of the internal capsule; SFO: Superior fronto-occipital fasciculus; SLF: Superior longitudinal fasciculus; SS: Sagittal stratum; UNC: Uncinate fasciculus. NDI: Neurite Density Index; ODI: Orientation Dispersion Index; ISOVF: Isotropic Volume Fraction. “UKB – Full”: Full sample (N=33 224); “UKB – Female”: Women only (N= 11 624); “UKB – Male”: Men only (N=10 514).[8]

Figure S5: Association of NODDI genome-wide significant SNPs with DTI metrics in the i-Share study

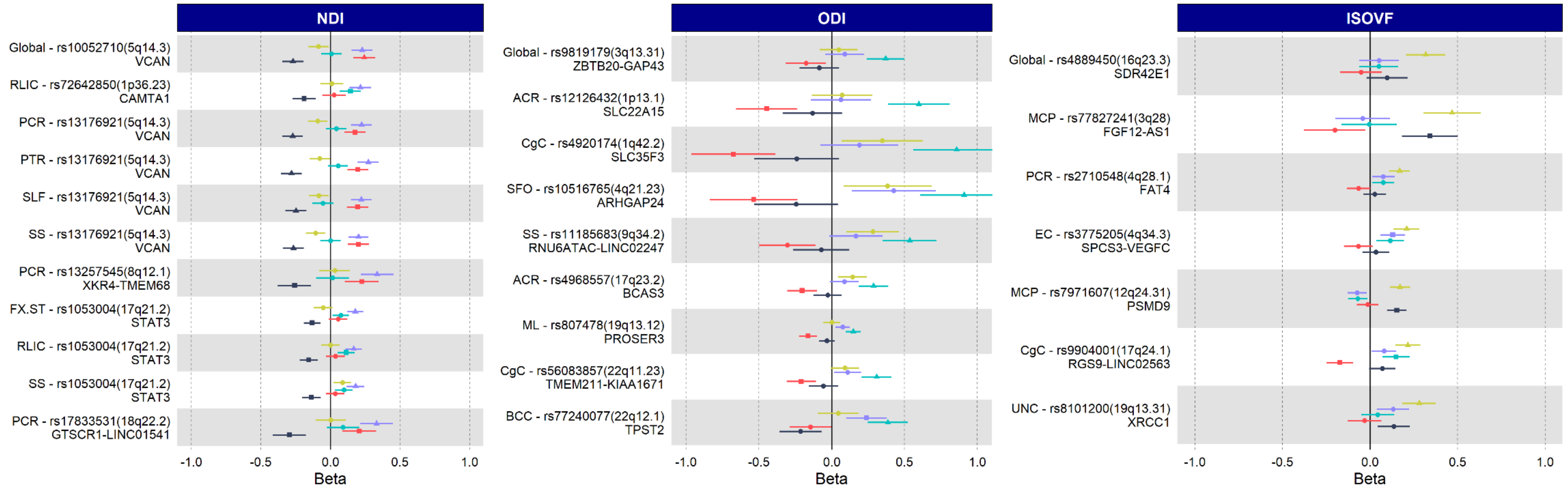

- ▲ p < 5e-8
- ■ p < 1.19e-3
- • p ≥ 1.19e-3

- ISOVF
- NDI
- ODI
- FA
- MD

ACR: Anterior corona radiata; BCC: Body corpus callosum; CgC: Cingulum cingulate gyrus; EC: External capsule; FX.ST: Fornix cres or stria terminalis; Global: global metrics; MCP: Middle cerebellar peduncle; ML: Medial lemniscus; PCR: Posterior corona radiata; PTR: Posterior thalamic radiation; RLIC: Retrolenticular part of the internal capsule; SFO: Superior fronto-occipital fasciculus; SLF: Superior longitudinal fasciculus; SS: Sagittal stratum; UNC: Uncinate fasciculus.
NDI: Neurite Density Index; ODI: Orientation Dispersion Index; ISOVF: Isotropic Volume Fraction

Figure S6: Transcriptome-wide association study (TWAS) of NODDI phenotypes in multiple tissues

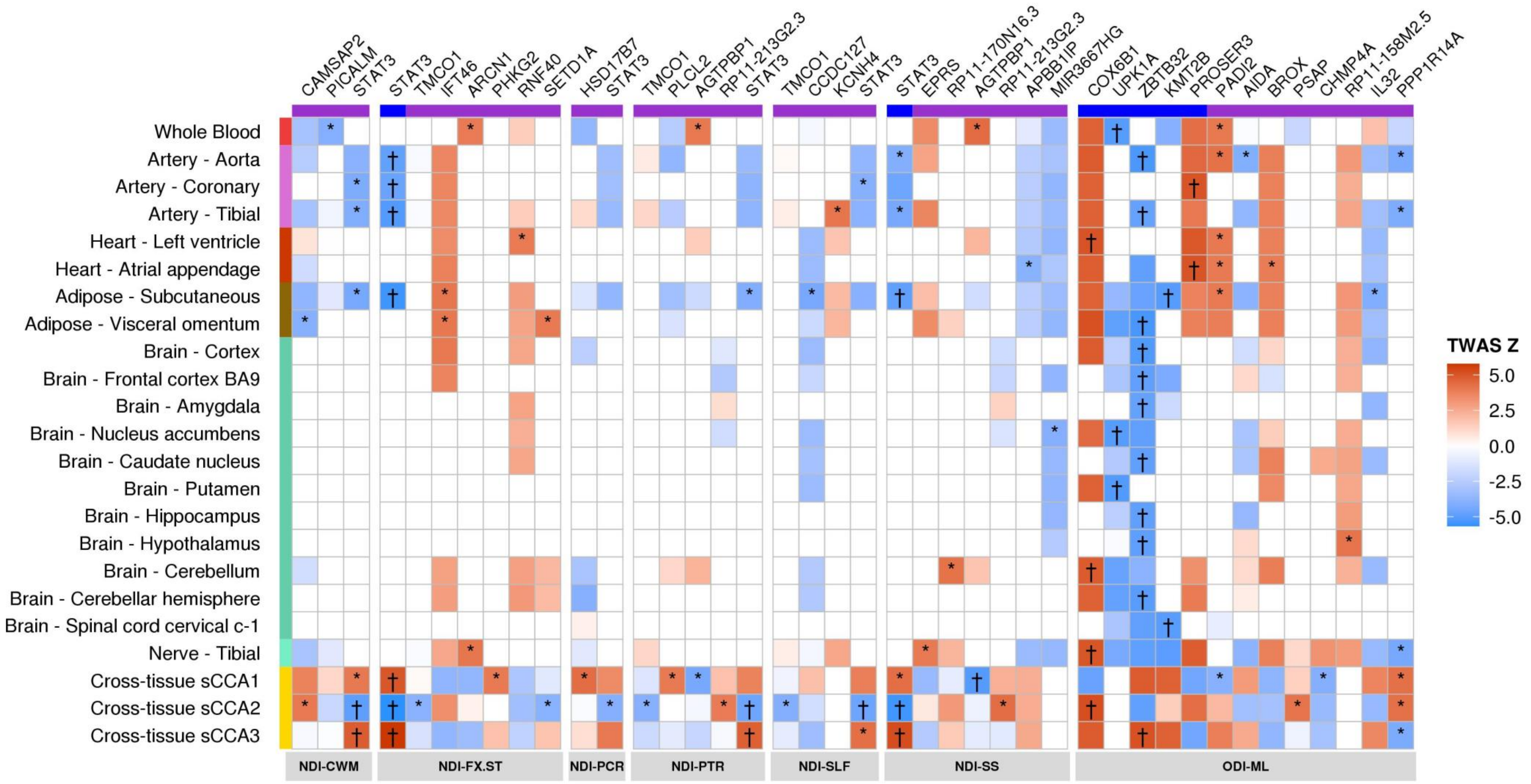

Heatmap of the transcriptome-wide association studies NODDI phenotypes with genome-wide significant loci in the i-Share study and replicated in the Rhineland study or UK Biobank. *: TWAS $p<1x10^{-4}$, p<0.05 in conditional analyses and COLOC-PP4>0.75. † TWAS $p<7.8x10^{-6}$, p<0.05 in conditional analyses and COLOC-PP4>0.75, accounting for the average number of genes tested across all the tissues. Only genes with * in at least one tissue for the corresponding phenotype are shown. Genes are presented on the x-axis, those underlined in blue are in a GWAS locus, those underlined in purple are not; Tissue types are on the y-axis (orange: blood; pink: arterial; dark orange: heart; brown: adipose, green: brain; turquoise blue: nerve; gold: cross-tissue weights). sCCA: Sparse canonical correlation analysis. NDI: Neurite Density Index; ODI: Orientation Dispersion Index; ISOVF: Isotropic Volume Fraction

Figure S7: Lifetime brain gene expression profile of genes in loci associated with NODDI phenotypes in the i-Share study with a correspondence in the Human Brain Transcriptome database

Figure S7A: NDI phenotypes

Nearest genes from top SNPs in GWAS loci

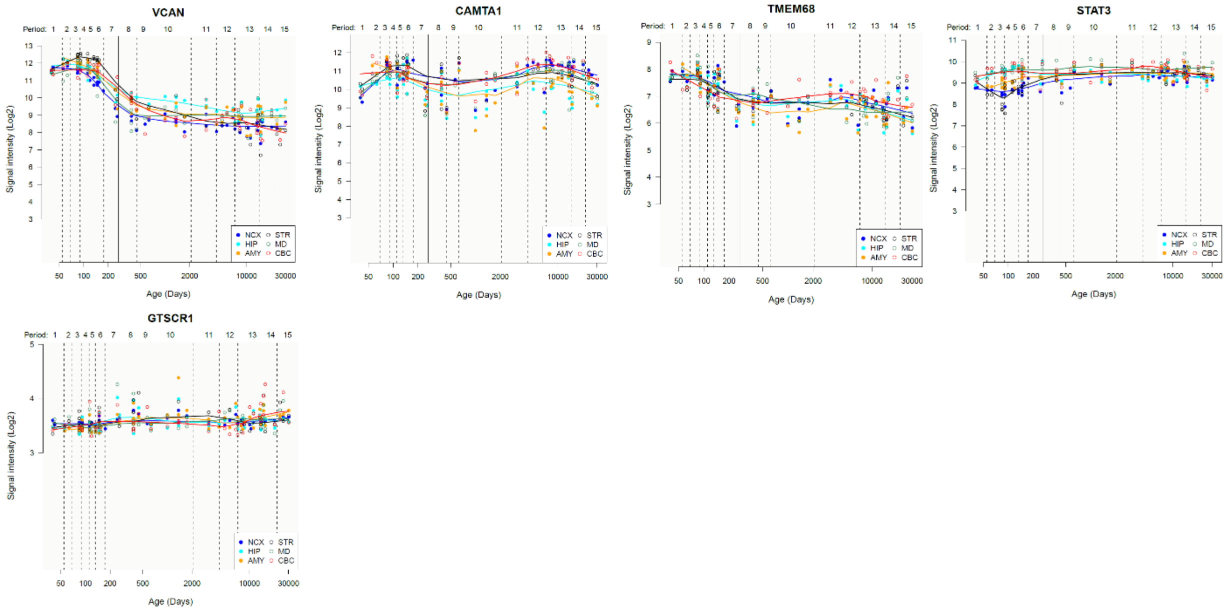

TWAS significant genes

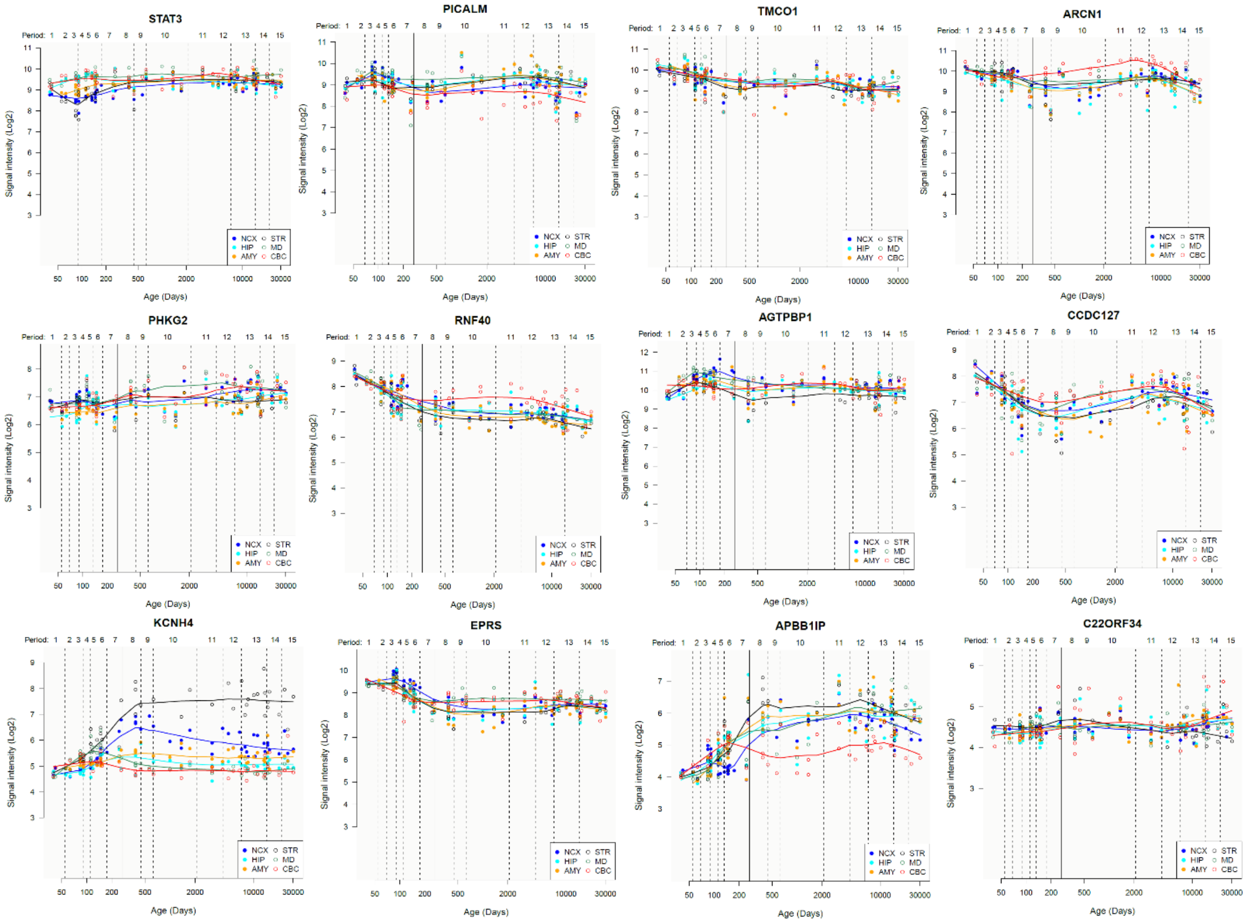

The gene *MIR3667HG* is named *C22orf34*.

Figure S7B: ODI phenotypes

Nearest genes from top SNPs in GWAS loci

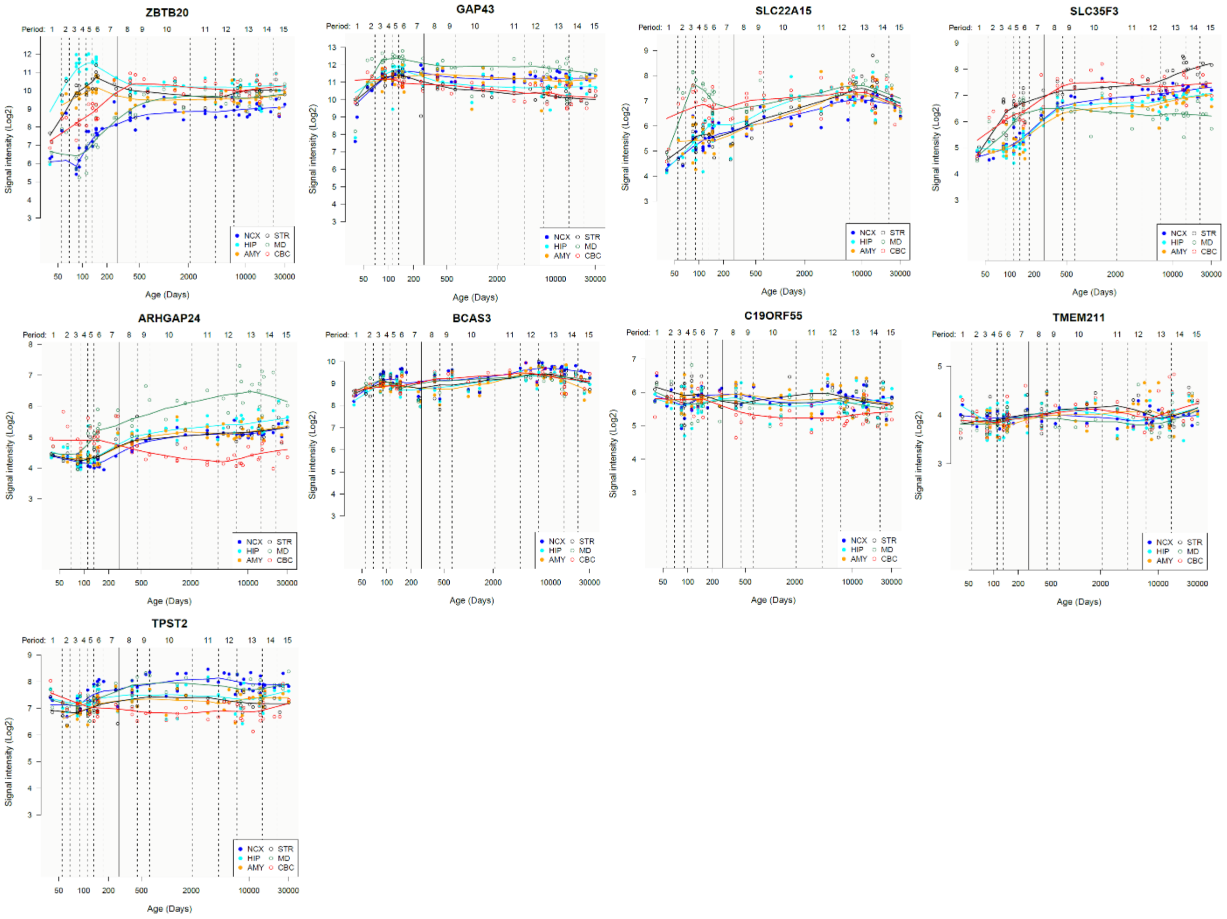

TWAS significant genes

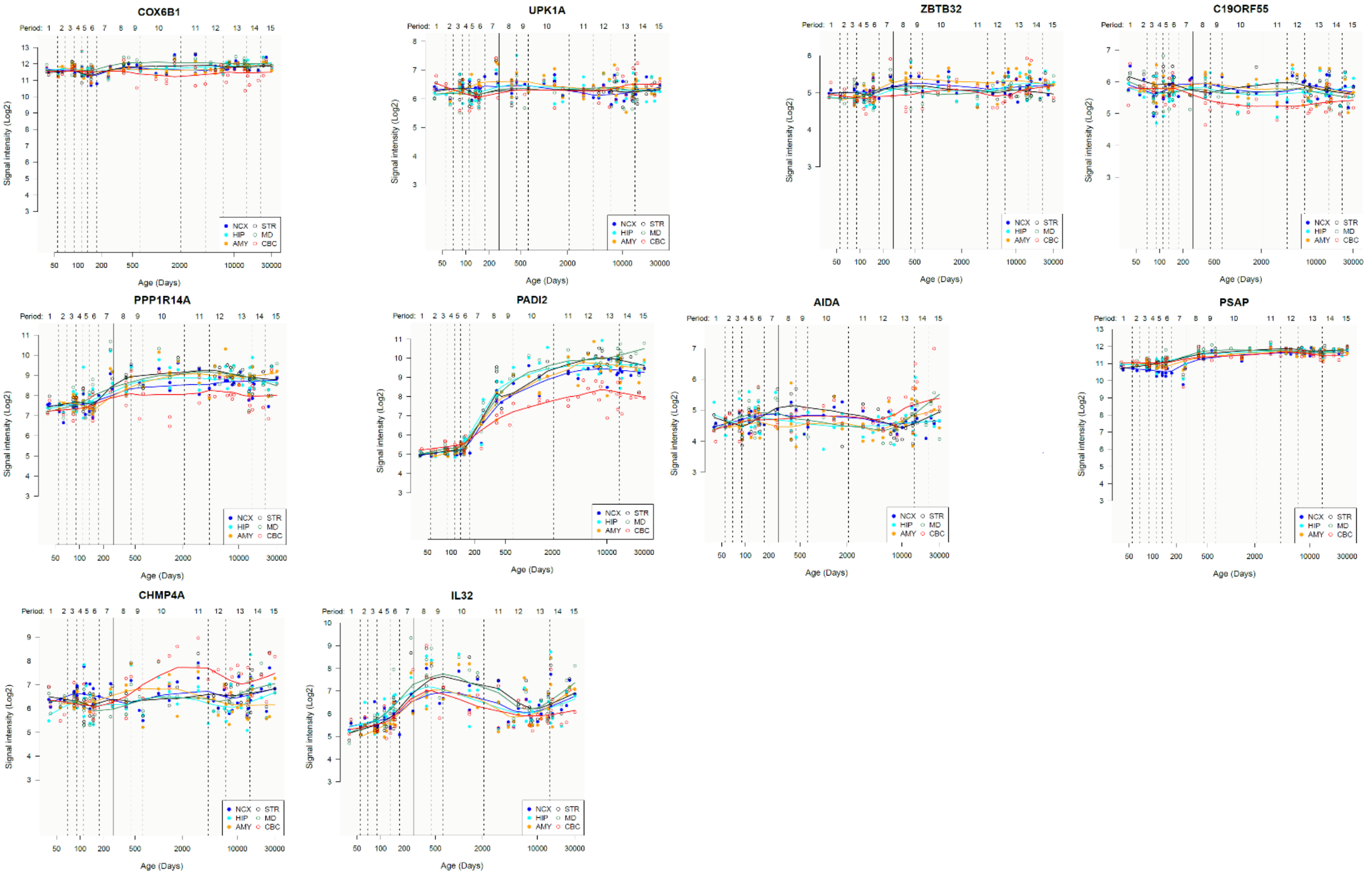

The gene *PROSER3* is named *C19orf55*.

Figure S7C: ISOVF phenotypes

Nearest genes from top SNPs in GWAS loci

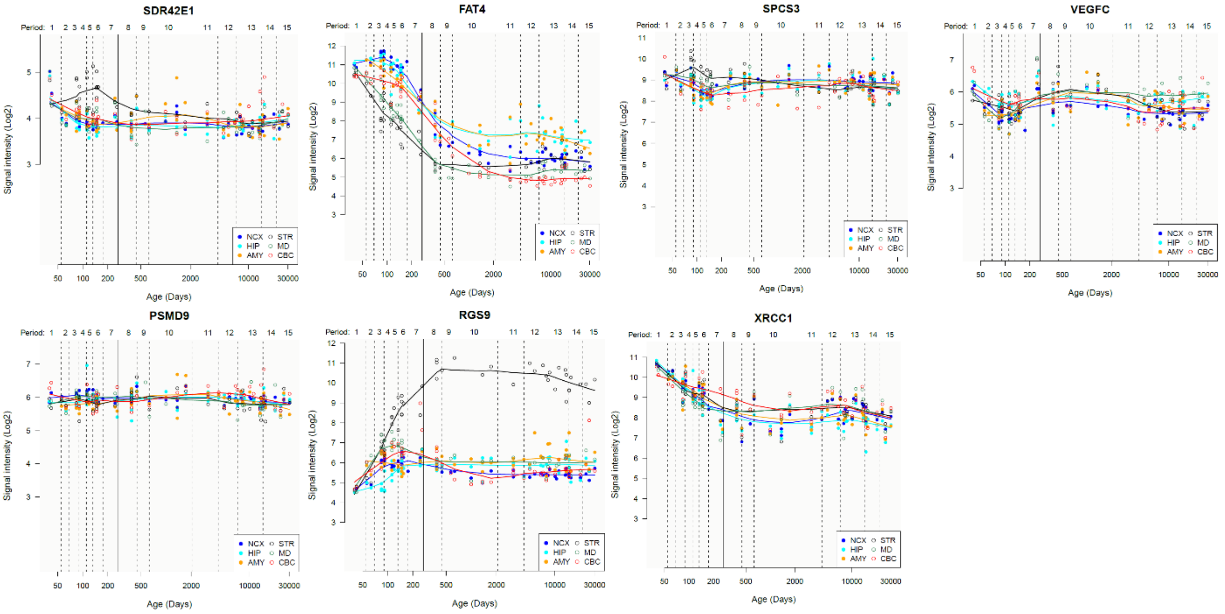

The spatio-temporal gene expression level is plotted as log2-transformed exon array signal intensity (y-axis) against the post conception days (x-axis) as provided by the Human Brain Transcriptome project database. Periods of human development and adulthood are indicated by vertical dashed lines: 4-8 post conception weeks [PCW] (period 1), 8-10 PCW (period 2), 10-13 PCW (period 3), 13-16 PCW (period 4), 16-19 PCW (period 5), 19-24 PCW (period 6), 24-38 PCW (period 7), birth- 6 postnatal months (period 8), 6-12 postnatal months (period 9), 1- 6 years (period 10), 6-12 years (period 11), 12-20 years (period 12), 20-40 years (period 13), 40-60 years (period 14), and 60 years+ (period 15). The boundary between pre- and postnatal periods is indicated by the solid vertical line. Each colored point represents the expression level of each gene across 16 anatomical brain regions and ages. Brain structure includes 11 neocortical areas (NCX, blue), and 5 subcortical regions: hippocampus (HIP, cyan), amygdala (AMY, orange), striatum (STR, black), mediodorsal nucleus of thalamus (MD, dark green), and cerebellar cortex (CBC, red). NDI: Neurite Density Index; ODI: Orientation Dispersion Index; ISOVF : Isotropic Volume Fraction

Figure S8: Association of neurovascular traits and vascular risk factors with NODDI metrics in young adults using genetic risk score and Mendelian randomization approaches

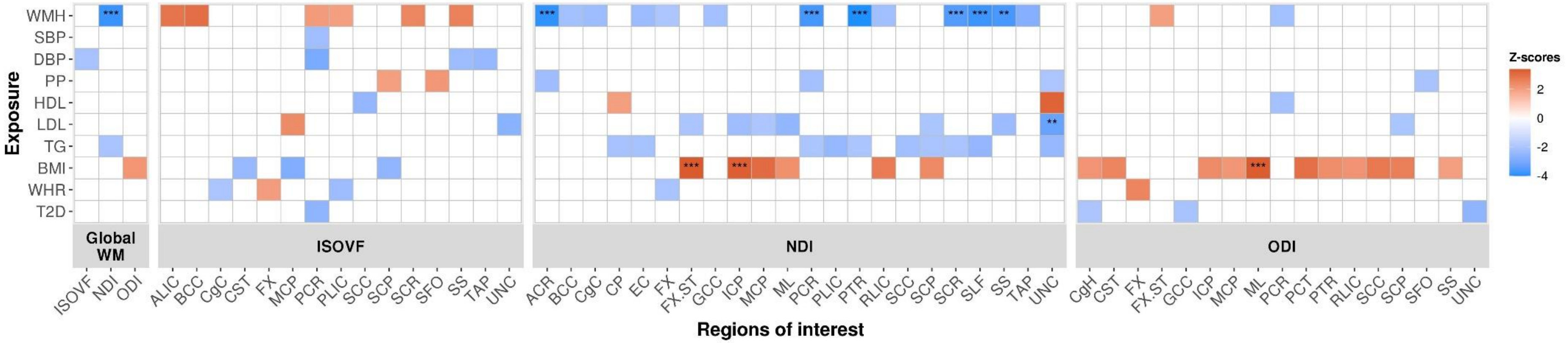

Only regions of interest with at least one nominally significant association are shown. Only results with p<0.05 for GRS are colored. Z-scores correspond to the effect of the GRS of the exposures on the NODDI phenotypes.

*: p<1.27E-03 with GRS and not significant in MR analyses. **: p<1.27E-03 with GRS and p<0.05 with at least one method between RadialMR IVW (after removing outliers) and GSMR . ***: p<1.27E-03 with GRS and p<0.05 with both RadialMR IVW and GSMR.

WMH: White Matter Hyperintensities; SBP: Systolic blood pressure; DBP: Diastolic blood pressure; PP: Pulse pressure; HDL: HDL-cholesterol; LDL: LDL-cholesterol; T2D: Type 2 diabetes; TG: Triglycerides; BMI: Body Mass Index; WHR: Waist-Hip Ratio.

NDI: Neurite Density Index; ODI: Orientation Dispersion Index; ISOVF: Isotropic Volume Fraction

Figure S9: Association of WMH and vascular risk factors with NODDI metrics in young adults (Mendelian randomization), i-Share study (n=1 758)

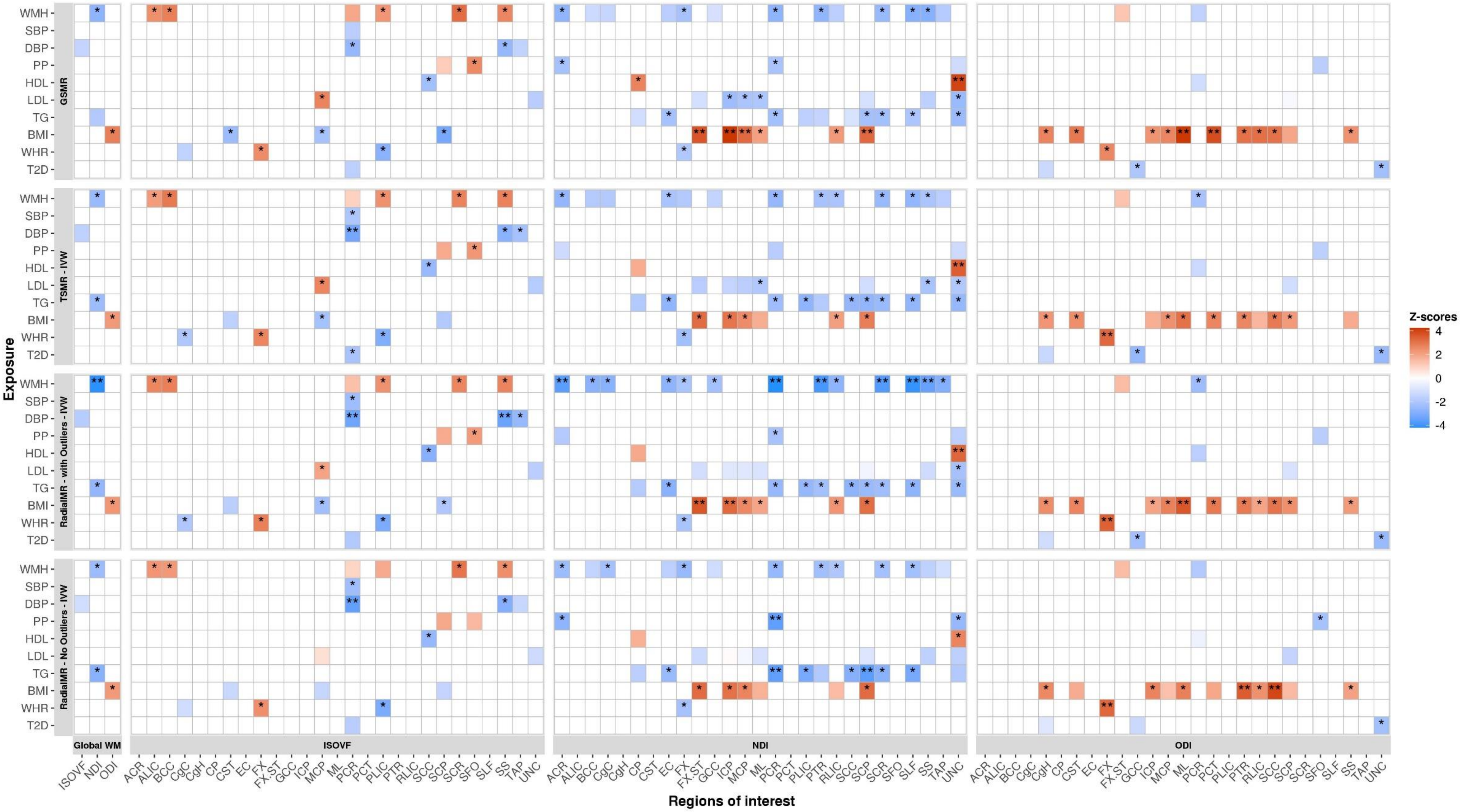

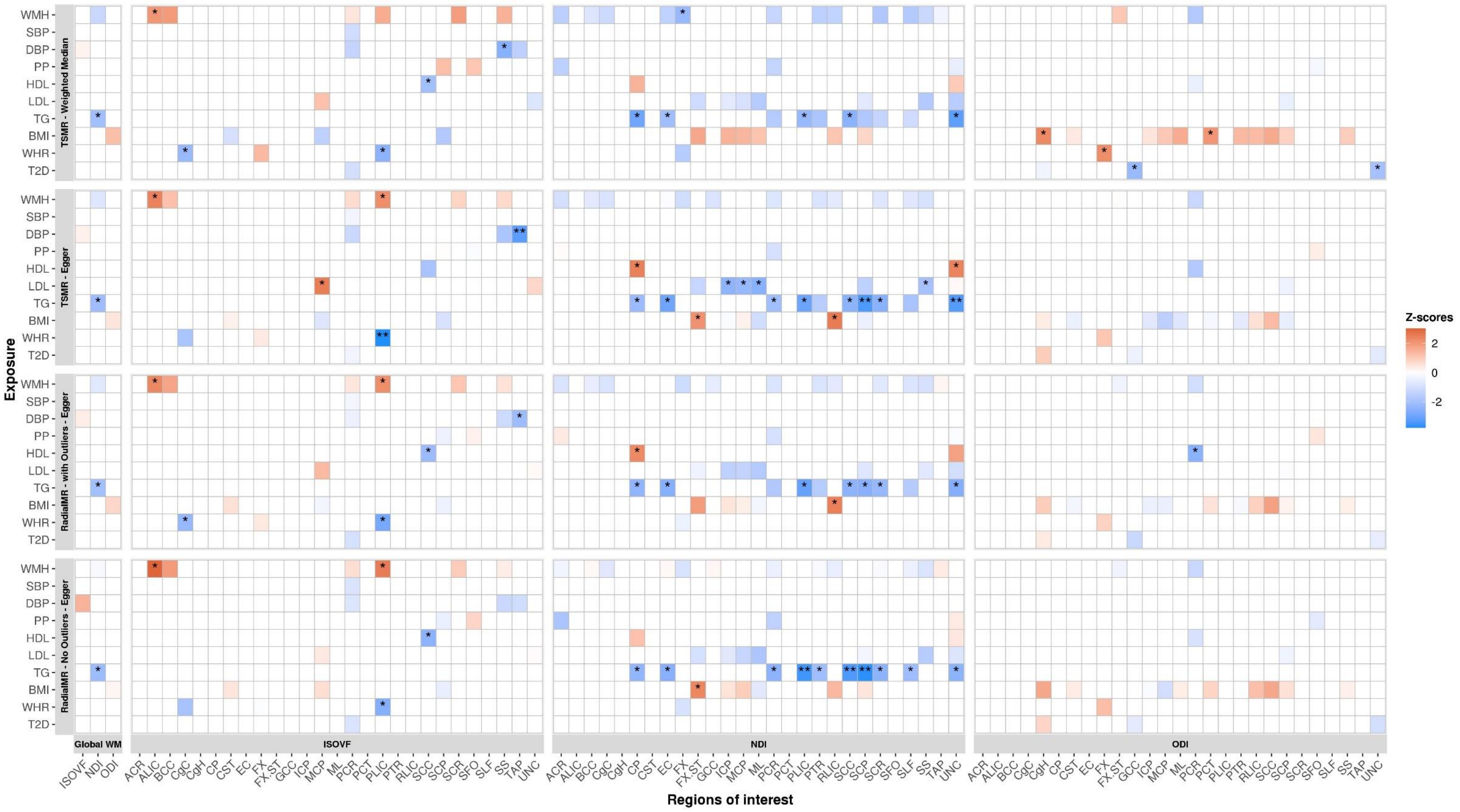

Only results with p<0.05 for genetic risk score analyses are colored. *: p < 0.05. **: p<1.27E-03.

WMH: White Matter Hyperintensities; SBP: Systolic blood pressure; DBP: Diastolic blood pressure; PP: Pulse pressure; HDL: HDL-cholesterol; LDL: LDL-cholesterol; T2D: Type 2 diabetes; TG: Triglycerides; BMI: Body Mass Index; WHR: Waist-Hip Ratio.

TSMR: TwoSampleMR; IVW: inverse variance weighted.

NDI: Neurite Density Index; ODI: Orientation Dispersion Index; ISOVF : Isotropic Volume Fraction

Figure S10: Correlation of mean WMH frequency for 3C-Dijon participants in each of the 27 JHU regions with Z-scores of association between the WMH GRS and NDI within each JHU region in the i-Share cohort

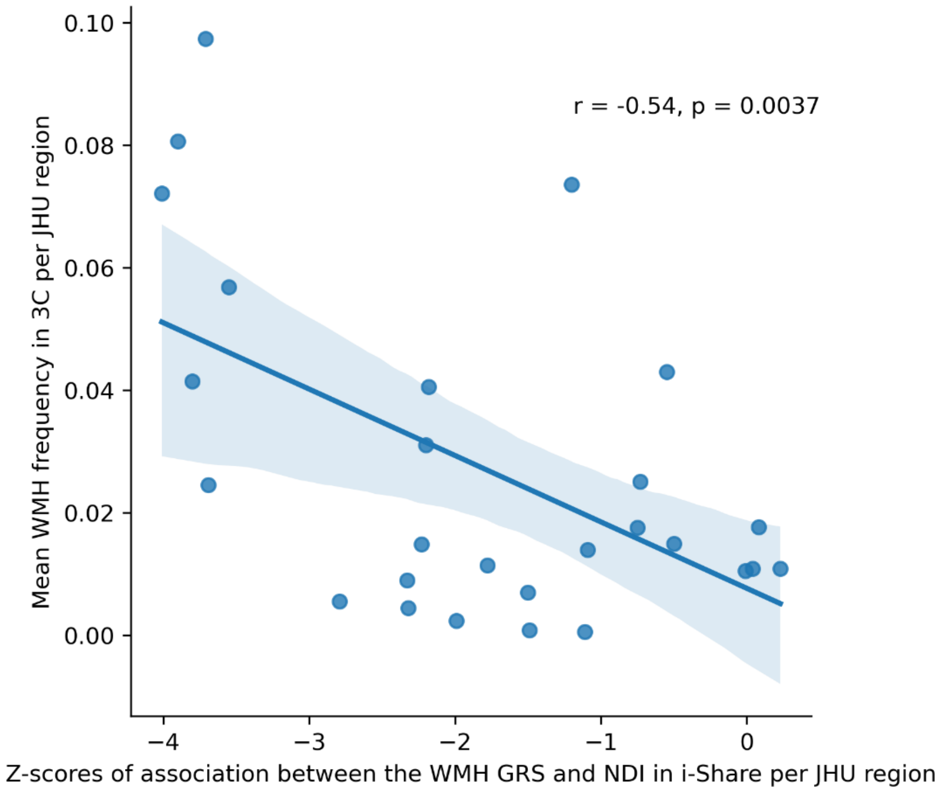

Supplementary tables

Table S1: Description of the 28 NODDI regions of interest (ROI)

Fibers type	ROI	Abbreviation
Global white matter	Cerebral white matter	CWM
Association fibers	Cingulum cingulate gyrus	CgC
	Cingulum hippocampus	CgH
	External capsule	EC
	Fornix	FX
	Fornix cres or stria terminalis	FX.ST
	Superior fronto-occipital fasciculus	SFO
	Superior longitudinal fasciculus	SLF
	Sagittal stratum	SS
	Uncinate fasciculus	UNC
Brainstem tracts	Corticospinal tract	CST
	Inferior cerebellar peduncle	ICP
	Middle cerebellar peduncle	MCP
	Medial lemniscus	ML
	Pontine crossing tract	PCT
	Superior cerebellar peduncle	SCP
Commissural fibers	Body corpus callosum	BCC
	Genu corpus callosum	GCC
	Splenium corpus callosum	SCC
	Tapetum	TAP
Projection fibers	Anterior corona radiata	ACR
	Anterior limb of the internal capsule	ALIC
	Cerebral peduncle	CP
	Posterior corona radiata	PCR
	Posterior limb of the internal capsule	PLIC
	Posterior thalamic radiation	PTR
	Retrolenticular part of the internal capsule	RLIC
	Superior corona radiata	SCR

Table S2: Association of lead variants (and proxies) from NODDI markers GWAS in young adults in GWAS of neurovascular traits and vascular risk factors

								i-Share GWAS		Lookup		
Lead SNP	SNP tested	Nearest gene	Locus	Position	A0	A1	FreqA1	Type NODDI	Z	Trait	Z	p
rs12126432	rs12126432	SLC22A15	1p13.1	116595405	G	C	0.97	ODI	5.53	AD	-2.52	1.17E-02
rs77827241	rs77827241	FGF12-AS1	3q28	191973700	T	C	0.04	ISOVF	5.60	HDL	-2.91	2.63E-02
rs9819179	rs9833318	ZBTB20;GAP43	3q13.31	115151530	T	C	0.94	ODI	5.27	BMI	-4.40	**1.48E-05**
rs9819179	rs9825881	ZBTB20;GAP43	3q13.31	115162379	G	A	0.95	ODI	4.62	SBP	-1.98	4.73E-02
rs9819179	rs28771126	ZBTB20;GAP43	3q13.31	115176561	G	T	0.97	ODI	4.93	PP	-2.94	3.21E-03
rs9819179	rs28771126	ZBTB20;GAP43	3q13.31	115176561	G	T	0.97	ODI	4.93	T2D	-3.27	8.40E-04
rs2710548	rs2710547	FAT4	4q28.1	126273372	T	G	0.58	ISOVF	5.56	LDL	-3.20	5.77E-03
rs3775205	rs12508325	SPCS3;VEGFC	4q34.3	177583120	G	A	0.20	ISOVF	5.33	DBP	-2.73	6.35E-03
rs3775205	rs959953	SPCS3;VEGFC	4q34.3	177601762	A	G	0.20	ISOVF	5.24	WHR	-2.78	4.61E-03
rs10052710	rs7733216	VCAN	5q14.3	82857870	C	T	0.78	NDI	5.84	WMH	-6.81	**9.73E-12**†
rs10052710	rs3852188	VCAN	5q14.3	82858014	G	C	0.81	NDI	6.34	PP	-2.58	9.93E-03
rs10052710	rs3852188	VCAN	5q14.3	82858014	G	C	0.81	NDI	6.34	SBP	-2.77	5.68E-03
rs10052710	rs13176921	VCAN	5q14.3	82860348	G	A	0.79	NDI	6.92	DBP	-2.07	3.94E-02
rs13176921	rs3852188	VCAN	5q14.3	82858014	G	C	0.81	NDI	6.34	PP	-2.58	9.93E-03
rs13176921	rs3852188	VCAN	5q14.3	82858014	G	C	0.81	NDI	6.34	SBP	-2.77	5.68E-03
rs13176921	rs3852188	VCAN	5q14.3	82858014	G	C	0.81	NDI	6.34	WMH	-6.75	**1.13E-11**†
rs13176921	rs13176921	VCAN	5q14.3	82860348	G	A	0.79	NDI	6.92	DBP	-2.07	3.94E-02
rs7971607	rs6486781	PSMD9	12q24.31	122318985	C	A	0.44	ISOVF	5.63	LDL	5.07	**1.24E-05**
rs7971607	rs6486781	PSMD9	12q24.31	122318985	C	A	0.44	ISOVF	5.63	PP	5.06	**4.00E-07**
rs7971607	rs10743186	PSMD9	12q24.31	122356436	T	C	0.42	ISOVF	5.41	SBP	5.31	**1.13E-07**
rs7971607	rs6486782	PSMD9	12q24.31	122359172	C	T	0.42	ISOVF	5.40	BMI	-3.71	2.44E-04
rs7971607	rs10770186	PSMD9	12q24.31	122361412	G	A	0.42	ISOVF	5.36	DBP	3.48	5.03E-04
rs1053004	rs1053004	STAT3	17q21.2	40466092	A	G	0.40	NDI	6.09	HDL	-3.26	1.29E-02
rs1053004	rs1053004	STAT3	17q21.2	40466092	A	G	0.40	NDI	6.09	LDL	3.69	1.47E-03
rs1053004	rs1053004	STAT3	17q21.2	40466092	A	G	0.40	NDI	6.09	MS*	0.12	**1.08E-11**†
rs1053004	rs1053004	STAT3	17q21.2	40466092	A	G	0.40	NDI	6.09	PP	-6.15	**7.90E-10**†
rs1053004	rs1053004	STAT3	17q21.2	40466092	A	G	0.40	NDI	6.09	SBP	-4.12	**3.78E-05**
rs4968557	rs4968557	BCAS3	17q23.2	59462087	A	G	0.87	ODI	5.54	DBP	3.82	**1.30E-04**
rs4968557	rs4968557	BCAS3	17q23.2	59462087	A	G	0.87	ODI	5.54	PP	4.55	**5.24E-06**
rs4968557	rs4968557	BCAS3	17q23.2	59462087	A	G	0.87	ODI	5.54	SBP	5.49	**4.12E-08**†
rs4968557	rs4968557	BCAS3	17q23.2	59462087	A	G	0.87	ODI	5.54	WHR	-3.91	**8.13E-05**
rs9904001	rs9904001	RGS9;LINC02563	17q24.1	63309198	G	A	0.80	ISOVF	5.88	WHR	2.30	2.34E-02
rs807478	rs173003	PROSER3	19q13.12	36240960	C	A	0.49	ODI	5.32	MS*	-0.04	7.22E-03
rs8101200	rs8101200	XRCC1	19q13.31	44051558	G	A	0.12	ISOVF	5.72	HDL	2.64	4.42E-02
rs77240077	rs77240077	TPST2	22q12.1	26944635	G	A	0.05	ODI	5.49	DBP	3.55	3.86E-04
rs77240077	rs77240077	TPST2	22q12.1	26944635	G	A	0.05	ODI	5.49	HDL	2.76	3.52E-02
rs77240077	rs77240077	TPST2	22q12.1	26944635	G	A	0.05	ODI	5.49	SBP	2.96	3.08E-03

A1: Effect allele; A0: Non-effect allele; FreqA1: frequency of A1; Z: Z-score defined as Beta of A1 / Standard error.
AD: Alzheimer disease; BMI: Body mass index; DBP: Diastolic blood pressure; MS: Multiple sclerosis; PP: Pulse pressure; SBP: Systolic blood pressure; T2D: type 2 diabetes; WHR: Waist-hip ratio; WMH: White matter hyperintensities.
NDI: Neurite Density Index; ODI: Orientation Dispersion Index; ISOVF: Isotropic Volume Fraction
* For multiple sclerosis, SE was not available in the summary statistics, beta is presented instead. †: Genome-wide significant pvalues (5×10^{-8}). $p<1.98\times10^{-4}$ are in **bold**. Only results with p<0.05 are shown. For i-Share, the Z-scores are presented for the region of interest with the lowest pvalue for the SNP tested.

Table S3: Association of lead variants from NODDI markers GWAS in cognitive tests in young adults

Cognitive test	SNP	Locus	Position	Gene	A0	A1	Beta	SE	p	N
**ISOVF loci**										
Stroop reaction time	rs2710548	4q28.1	126273530	FAT4	C	T	0.08	0.04	0.023	1365
Verbal learning test (immediate recall)	rs8101200	19q13.31	44051558	XRCC1	G	A	0.14	0.06	0.018	1268
**NDI loci**										
Stroop reaction time	rs10052710	5q14.3	82860025	VCAN	G	T	-0.09	0.04	0.050	1365
Verbal learning test (immediate recall)	rs1053004	17q21.2	40466092	STAT3	A	G	0.08	0.04	0.037	1268
**ODI loci**										
Matrices	rs11185683	9q34.2	137131455	RNU6ATAC;LINC02247	A	G	0.51	0.11	**3.94E-06**	1300
Vocabulary	rs807478	19q13.12	36252494	PROSER3	G	A	0.08	0.04	0.020	1368
Matrices	rs56083857	22q11.23	25386309	TMEM211;KIAA1671	T	C	-0.15	0.06	0.011	1300
Rotation	rs56083857	22q11.23	25386309	TMEM211;KIAA1671	T	C	-0.12	0.06	0.039	1332

Only results with $p \leq 0.05$ are shown. $p < 2.98 \times 10^{-4}$ are in bold. A1: effect allele; A0: non-effect allele; Beta: effect for A1; SE: Standard error. NDI: Neurite Density Index; ODI: Orientation Dispersion Index; ISOVF: Isotropic Volume Fraction.

Table S4: Significant results of transcriptome-wide association study of NODDI for phenotypes presenting replicated genome-wide significant loci in GWAS

Tissue	Gene	CHR	Start	Stop	NWGT	MODEL	TWAS.Z	TWAS.P	JOINT.Z	JOINT.P	COLOC.PP4
**NDI - CWM**											
Adipose - Visceral omentum	CAMSAP2	1	200708686	200829832	9	lasso	-3.96	7.45E-05	-4.00	7.40E-05	0.83
Cross-tissue sCCA2	CAMSAP2	1	200708686	200829832	9	lasso	3.98	6.97E-05	4.00	6.90E-05	0.84
Whole Blood	PICALM	11	85668727	85780924	1	top1	-4.03	5.70E-05	-4.00	5.70E-05	0.95
Adipose - Subcutaneous	STAT3	17	40465342	40540586	1	top1	-4.38	1.17E-05	-4.40	1.20E-05	0.88
Artery - Coronary	STAT3	17	40465342	40540586	20	enet	-4.22	2.46E-05	-4.20	2.50E-05	0.81
Artery - Tibial	STAT3	17	40465342	40540586	261	susie	-4.13	3.62E-05	-4.10	3.60E-05	0.95
Cross-tissue sCCA1	STAT3	17	40465342	40540586	1	top1	3.99	6.69E-05	4.00	6.70E-05	0.94
Cross-tissue sCCA2	STAT3	17	40465342	40540586	32	enet	-4.91	**8.92E-07**	-4.90	8.90E-07	1.00
Cross-tissue sCCA3	STAT3	17	40465342	40540586	7	lasso	4.83	**1.40E-06**	4.80	1.40E-06	0.92
**NDI - FX.ST**											
Cross-tissue sCCA2	TMCO1	1	165696032	165796992	1	top1	-3.90	9.74E-05	-3.90	9.70E-05	0.95
Adipose - Subcutaneous	IFT46	11	118415243	118443685	12	lasso	4.07	4.60E-05	4.10	4.60E-05	0.93
Adipose - Visceral omentum	IFT46	11	118415243	118443685	1	top1	4.05	5.10E-05	4.10	5.10E-05	0.97
Nerve - Tibial	ARCN1	11	118443105	118473748	1	top1	4.09	4.41E-05	4.10	4.40E-05	0.93
Whole Blood	ARCN1	11	118443105	118473748	1	top1	3.91	9.15E-05	3.90	9.20E-05	0.92
Cross-tissue sCCA1	PHKG2	16	30759591	30772490	1	top1	3.95	7.72E-05	4.00	7.70E-05	0.89
Heart - Left ventricle	RNF40	16	30773066	30787628	1	top1	3.92	9.00E-05	3.90	9.00E-05	0.91
Adipose - Visceral omentum	SETD1A	16	30968615	30996437	1	top1	3.95	7.72E-05	4.00	7.70E-05	0.97
Cross-tissue sCCA2	SETD1A	16	30968615	30996437	1	top1	-3.92	9.00E-05	-3.90	9.00E-05	0.84
Artery - Aorta	STAT3	17	40465342	40540586	259	susie	-4.83	**1.36E-06**	-4.80	1.40E-06	0.91
Artery - Coronary	STAT3	17	40465342	40540586	20	enet	-4.67	**3.06E-06**	-4.70	3.10E-06	0.81
Artery - Tibial	STAT3	17	40465342	40540586	261	susie	-5.13	**2.96E-07**	-5.10	2.90E-07	0.97
Cross-tissue sCCA1	STAT3	17	40465342	40540586	1	top1	4.98	**6.49E-07**	5.00	6.50E-07	0.95
Cross-tissue sCCA2	STAT3	17	40465342	40540586	32	enet	-5.66	**1.51E-08**	-5.70	1.50E-08	1.00
Cross-tissue sCCA3	STAT3	17	40465342	40540586	7	lasso	5.77	**7.92E-09**	5.80	7.90E-09	0.95
Adipose - Subcutaneous	STAT3	17	40465342	40540586	1	top1	-5.45	**5.12E-08**	-5.40	5.10E-08	0.96
**NDI - PCR**											
Cross-tissue sCCA1	HSD17B7	1	162760492	162782607	50	enet	4.35	1.36E-05	4.30	1.40E-05	0.81
Cross-tissue sCCA2	STAT3	17	40465342	40540586	32	enet	-4.01	6.10E-05	-4.00	6.10E-05	0.94
**NDI - PTR**											
Cross-tissue sCCA2	TMCO1	1	165696032	165796992	1	top1	-3.96	7.49E-05	-4.00	7.50E-05	0.96
Cross-tissue sCCA1	PLCL2	3	16844159	17132086	11	lasso	3.96	7.58E-05	4.00	7.60E-05	0.95
Cross-tissue sCCA1	AGTPBP1	9	88161455	88356944	7	lasso	-4.41	1.05E-05	-4.40	1.00E-05	0.96
Whole Blood	AGTPBP1	9	88161455	88356944	358	susie	4.01	5.99E-05	4.00	6.00E-05	0.85
Cross-tissue sCCA2	RP11-213G2.3	9	88430881	88464426	1	top1	3.91	9.15E-05	3.90	9.20E-05	0.77
Adipose - Subcutaneous	STAT3	17	40465342	40540586	1	top1	-4.09	4.41E-05	-4.10	4.40E-05	0.78

Tissue	Gene	CHR	Start	Stop	NWGT	MODEL	TWAS.Z	TWAS.P	JOINT.Z	JOINT.P	COLOC.PP4
Cross-tissue sCCA2	STAT3	17	40465342	40540586	32	enet	-4.59	**4.42E-06**	-4.60	4.40E-06	0.99
Cross-tissue sCCA3	STAT3	17	40465342	40540586	7	lasso	4.69	**2.74E-06**	4.70	2.70E-06	0.86
**NDI - SLF**											
Cross-tissue sCCA2	TMCO1	1	165696032	165796992	1	top1	-4.04	5.30E-05	-4.00	5.30E-05	0.97
Adipose - Subcutaneous	CCDC127	5	196986	218330	10	lasso	-4.36	1.31E-05	-4.40	1.30E-05	0.97
Artery - Tibial	KCNH4	17	40308909	40333296	17	enet	4.11	3.95E-05	4.10	4.00E-05	0.76
Artery - Coronary	STAT3	17	40465342	40540586	20	enet	-3.97	7.25E-05	-4.00	7.20E-05	0.81
Cross-tissue sCCA2	STAT3	17	40465342	40540586	32	enet	-4.60	**4.22E-06**	-4.60	4.20E-06	0.99
Cross-tissue sCCA3	STAT3	17	40465342	40540586	7	lasso	4.27	1.99E-05	4.30	2.00E-05	0.83
**NDI - SS**											
Nerve - Tibial	EPRS	1	220141943	220220000	30	enet	3.94	8.12E-05	3.90	8.10E-05	0.82
Brain - Cerebellum	RP11-170N16.3	4	123803033	123805867	21	enet	4.17	3.06E-05	4.20	3.10E-05	0.79
Cross-tissue sCCA1	AGTPBP1	9	88161455	88356944	7	lasso	-5.08	**3.87E-07**	-5.10	3.90E-07	0.98
Whole Blood	AGTPBP1	9	88161455	88356944	358	susie	4.38	1.17E-05	4.40	1.20E-05	0.92
Cross-tissue sCCA2	RP11-213G2.3	9	88430881	88464426	1	top1	4.35	1.35E-05	4.40	1.30E-05	0.84
Heart - Atrial appendage	APBB1IP	10	26727132	26856732	433	susie	-3.92	8.86E-05	-3.90	8.90E-05	0.90
Artery - Aorta	STAT3	17	40465342	40540586	259	susie	-4.05	5.05E-05	-4.10	5.00E-05	0.88
Artery - Tibial	STAT3	17	40465342	40540586	261	susie	-4.37	1.25E-05	-4.40	1.30E-05	0.96
Cross-tissue sCCA1	STAT3	17	40465342	40540586	1	top1	4.30	1.68E-05	4.30	1.70E-05	0.92
Cross-tissue sCCA2	STAT3	17	40465342	40540586	32	enet	-5.42	**5.89E-08**	-5.40	5.90E-08	1.00
Cross-tissue sCCA3	STAT3	17	40465342	40540586	7	lasso	5.22	**1.82E-07**	5.20	1.80E-07	0.95
Adipose - Subcutaneous	STAT3	17	40465342	40540586	1	top1	-4.85	**1.22E-06**	-4.90	1.20E-06	0.93
Brain - Nucleus accumbens	MIR3667HG	22	49808176	50051190	3	lasso	-4.00	6.40E-05	-4.00	6.40E-05	0.78
**ODI - ML**											
Adipose - Subcutaneous	PADI2	1	17393256	17445948	1	top1	3.95	7.82E-05	4.00	7.80E-05	0.95
Artery - Aorta	PADI2	1	17393256	17445948	19	enet	4.22	2.44E-05	4.20	2.40E-05	0.96
Heart - Atrial appendage	PADI2	1	17393256	17445948	1	top1	3.95	7.82E-05	4.00	7.80E-05	0.96
Heart - Left ventricle	PADI2	1	17393256	17445948	1	top1	4.01	6.15E-05	4.00	6.10E-05	0.97
Cross-tissue sCCA1	PADI2	1	17393256	17445948	1	top1	-3.95	7.82E-05	-4.00	7.80E-05	0.96
Whole Blood	PADI2	1	17393256	17445948	383	susie	3.99	6.60E-05	4.00	6.60E-05	0.96
Artery - Aorta	AIDA	1	222841355	222886552	7	lasso	-4.08	4.51E-05	-4.10	4.50E-05	0.94
Heart - Atrial appendage	BROX	1	222885895	222908538	321	susie	3.90	9.51E-05	3.90	9.50E-05	0.95
Cross-tissue sCCA2	PSAP	10	73576055	73611126	4	lasso	4.15	3.28E-05	4.20	3.30E-05	0.91
Cross-tissue sCCA1	CHMP4A	14	24678789	24683075	22	enet	-3.99	6.53E-05	-4.00	6.50E-05	0.93
Brain - Hypothalamus	RP11-158M2.5	15	86298172	86299468	561	susie	4.12	3.81E-05	4.10	3.80E-05	0.98
Adipose - Subcutaneous	IL32	16	3115298	3131908	22	enet	-4.09	4.27E-05	-4.10	4.30E-05	0.84
Brain - Cerebellum	COX6B1	19	36139125	36149763	1	top1	4.86	**1.16E-06**	4.90	1.20E-06	0.97
Heart - Left ventricle	COX6B1	19	36139125	36149763	25	enet	5.09	**3.56E-07**	5.10	3.60E-07	0.99
Nerve - Tibial	COX6B1	19	36139125	36149763	1	top1	5.02	**5.25E-07**	5.00	5.20E-07	0.98
Cross-tissue sCCA2	COX6B1	19	36139125	36149763	1	top1	5.20	**1.96E-07**	5.20	2.00E-07	0.99
Brain - Nucleus accumbens	UPK1A	19	36157715	36169367	2	lasso	-5.07	**3.92E-07**	-5.10	3.90E-07	0.97
Brain - Putamen	UPK1A	19	36157715	36169367	445	susie	-5.14	**2.69E-07**	-5.10	2.70E-07	0.99

Tissue	Gene	CHR	Start	Stop	NWGT	MODEL	TWAS.Z	TWAS.P	JOINT.Z	JOINT.P	COLOC.PP4
Whole Blood	UPK1A	19	36157715	36169367	17	enet	-5.06	**4.11E-07**	-5.10	4.10E-07	1.00
Adipose - Visceral omentum	ZBTB32	19	36195429	36207940	1	top1	-5.32	**1.05E-07**	-5.30	1.10E-07	1.00
Artery - Aorta	ZBTB32	19	36195429	36207940	1	top1	-5.27	**1.36E-07**	-5.30	1.40E-07	1.00
Artery - Tibial	ZBTB32	19	36195429	36207940	6	lasso	-4.77	**1.87E-06**	-4.80	1.90E-06	0.97
Brain - Amygdala	ZBTB32	19	36195429	36207940	440	susie	-4.77	**1.86E-06**	-4.80	1.90E-06	0.88
Brain - Caudate nucleus	ZBTB32	19	36195429	36207940	1	top1	-4.86	**1.16E-06**	-4.90	1.20E-06	0.96
Brain - Cerebellar hemisphere	ZBTB32	19	36195429	36207940	435	susie	-5.02	**5.09E-07**	-5.00	5.10E-07	0.99
Brain - Cortex	ZBTB32	19	36195429	36207940	437	susie	-5.16	**2.46E-07**	-5.20	2.50E-07	0.99
Brain - Frontal cortex BA9	ZBTB32	19	36195429	36207940	1	top1	-4.86	**1.16E-06**	-3.40	6.70E-04	0.86
Brain - Hippocampus	ZBTB32	19	36195429	36207940	1	top1	-4.86	**1.16E-06**	-4.90	1.20E-06	0.94
Brain - Hypothalamus	ZBTB32	19	36195429	36207940	4	lasso	-4.90	**9.35E-07**	-4.90	9.40E-07	0.95
Cross-tissue sCCA3	ZBTB32	19	36195429	36207940	4	lasso	5.11	**3.31E-07**	5.10	3.30E-07	0.99
Adipose - Subcutaneous	KMT2B	19	36208921	36229779	440	susie	-5.06	**4.18E-07**	-5.10	4.20E-07	0.99
Brain - Spinal cord cervical c-1	KMT2B	19	36208921	36229779	431	susie	-5.05	**4.41E-07**	-5.10	4.40E-07	0.99
Artery - Coronary	PROSER3	19	36249044	36261930	1	top1	5.04	**4.70E-07**	5.00	4.70E-07	0.99
Heart - Atrial appendage	PROSER3	19	36249044	36261930	1	top1	5.04	**4.70E-07**	5.00	4.70E-07	0.99
Artery - Aorta	PPP1R14A	19	38741877	38747231	13	enet	-4.26	2.08E-05	-4.30	2.10E-05	0.96
Artery - Tibial	PPP1R14A	19	38741877	38747231	1	top1	-4.20	2.62E-05	-4.20	2.60E-05	0.98
Nerve - Tibial	PPP1R14A	19	38741877	38747231	9	enet	-4.38	1.20E-05	-4.40	1.20E-05	0.96
Cross-tissue sCCA1	PPP1R14A	19	38741877	38747231	1	top1	4.24	2.27E-05	4.20	2.30E-05	0.98
Cross-tissue sCCA2	PPP1R14A	19	38741877	38747231	3	lasso	4.25	2.18E-05	4.20	2.20E-05	0.81
Cross-tissue sCCA3	PPP1R14A	19	38741877	38747231	2	lasso	-4.23	2.35E-05	-4.20	2.40E-05	0.98

TWAS.P in **bold** are significant after correction for multiple testing ($7.8x10^{-6}$). Only conditionally significant ($p<0.05$) and colocalized (COLOC.PP4>0.75) results are shown.

TWAS.Z: Z-score from TWAS analyses; TWAS.P: pvalue from TWAS analyses; JOINT.Z: Z-score from conditional analyses; Joint.P: pvalue from conditional analyses; COLOC.PP4: Posterior probability 4 from colocalization analyses, corresponding to the probability to colocalized functional/GWAS associations; MODEL: Best performing model; NWGT: Number of SNPs with non-zero weights in the model

NDI: Neurite Density Index; ODI: Orientation Dispersion Index; ISOVF: Isotropic Volume Fraction

Table S5: Association of known WMH risk variants and global NODDI markers in young adults (i-Share study, n=1 758)

							NDI		ODI		ISOVF	
SNP	Locus	Pos	Nearest gene	A1	A0	FreqA1	Z	p	Z	p	Z	p
rs786921	1p22.2	89286673	PKN2	A	G	0.56	-0.13	8.93E-01	0.11	9.10E-01	0.98	3.28E-01
rs73923006	2p21	43132224	HAAO	G	C	0.82	-0.01	9.95E-01	2.10	**3.59E-02**	1.61	1.08E-01
rs7596872	2p16.1	56128091	EFEMP1	A	C	0.10	-0.03	9.79E-01	1.82	6.84E-02	0.24	8.11E-01
rs62172472	2q32.1	188028317	CALCRL	G	A	0.81	-0.05	9.60E-01	-0.32	7.52E-01	-1.81	6.95E-02
rs7603972	2q33.2	203780515	CARF1	A	G	0.86	0.62	5.34E-01	0.62	5.37E-01	0.02	9.87E-01
rs6797002	3q27.1	183363263	KLHL24									
rs17205972	5q14.3	82859065	VCAN	T	G	0.19	-5.68	**1.32E-08***	0.02	9.81E-01	2.46	**1.38E-02**
rs2303655	5q23.2	121518378	LOC100505841	T	C	0.79	-0.13	8.98E-01	-1.51	1.30E-01	-0.13	8.96E-01
rs6940540	6q25.1	151018909	PLEKHG1	G	T	0.42	-0.14	8.85E-01	1.38	1.69E-01	0.35	7.24E-01
rs73184312	8p23.1	8179639	SGK223	G	A	0.76	-0.76	4.47E-01	0.43	6.64E-01	-0.35	7.24E-01
rs11249945	8p23.1	9628753	TNKS	A	G	0.35	-0.97	3.34E-01	-0.90	3.66E-01	-0.39	6.98E-01
rs7004825	8p23.1	11031472	XKR6	T	C	0.47	-1.17	2.41E-01	-0.83	4.06E-01	0.52	6.05E-01
rs4630220	10q24.33	105459116	SH3PXD2A	G	A	0.73	-1.16	2.47E-01	1.14	2.56E-01	0.79	4.29E-01
rs71471298	10q24.33	105507145	SH3PXD2A-AS1	T	C	0.11	-2.61	**9.18E-03**	0.11	9.16E-01	0.43	6.70E-01
rs10786772	10q24.33	105610326	SH3PXD2A	G	A	0.69	-1.68	9.25E-02	0.44	6.61E-01	2.40	**1.64E-02**
rs55940034	13q34	111043309	COL4A2	G	A	0.31	2.14	**3.25E-02**	0.79	4.30E-01	-0.66	5.07E-01
rs72680374	14q22.1	52604843	NID2d	T	A	0.66	0.08	9.39E-01	-1.27	2.02E-01	-0.24	8.12E-01
rs1285847	14q32.11	91884655	CCDC88C	T	C	0.55	-0.53	5.94E-01	0.36	7.22E-01	0.65	5.18E-01
rs7157599	14q32.2	100625902	DEGS2	C	T	0.29	0.37	7.10E-01	1.54	1.24E-01	0.95	3.40E-01
rs12443113	15q22.31	65355468	RASL12	G	A	0.56	-0.71	4.75E-01	1.18	2.39E-01	0.80	4.25E-01
rs1948948	16q12.1	51442679	SALL1	C	T	0.58	0.12	9.07E-01	0.51	6.12E-01	-2.67	**7.59E-03**
rs12921170	16q24.2	87227397	C16orf95	A	G	0.57	-1.50	1.32E-01	-0.82	4.15E-01	0.24	8.07E-01
rs6503417	17q21.31	43144218	NMT1	C	T	0.63	-3.41	**6.46E-04***	-0.41	6.81E-01	2.00	**4.56E-02**
rs34974290	17q25.1	73888354	TRIM65	A	G	0.22	-1.16	2.44E-01	-0.14	8.87E-01	-0.40	6.87E-01
rs5762197	22q12.1	27887471	MN1	C	A	0.65	0.12	9.04E-01	1.49	1.37E-01	-0.11	9.09E-01

A1: effect allele (aligned with allele increasing WMH risk); A0: non-effect allele; Freq: frequency of A1; Z: Z-score defined as Beta of A1 / Standard error.

NDI: Neurite Density Index; ODI: Orientation Dispersion Index; ISOVF: Isotropic Volume Fraction

Nominally significant results are in bold. *: Significant results ($p<2x10^{-3}$).

Table S6: Association of WMH and vascular risk factors with NODDI metrics in young adults, i-Share study (n=1 758)

									Egger intercept	
ROI	Exposure	Methods	N SNPs	Mean F	Z	p	Q	p(Q)	Beta	p
**NDI**										
ACR	WMH	GRS	23		-3.90	**9.63E-05**				
ACR	WMH	GSMR	22		-2.71	**6.70E-03**				
ACR	WMH	RadialMR - with outliers - IVW	22	55.0	-3.67	**2.40E-04**	34.1	0.0355		
ACR	WMH	RadialMR - with outliers - Egger	22	55.0	-0.86	4.02E-01	34.0	0.0365	0.10	9.25E-01
ACR	WMH	RadialMR - no outliers - IVW	19	55.2	-2.44	**1.47E-02**	13.4	0.7670		
ACR	WMH	RadialMR - no outliers - Egger	19	55.2	-0.33	7.46E-01	13.3	0.7751	-0.34	6.27E-01
ACR	WMH	TSMR - WM	22		-1.56	1.20E-01				
ACR	WMH	TSMR - IVW	22		-2.66	**7.74E-03**	33.2	0.0444		
ACR	WMH	TSMR - Egger	22		-0.98	3.37E-01	33.1	0.0325	0.00	9.10E-01
ACR	PP	GRS	666		-2.26	**2.40E-02**				
ACR	PP	GSMR	788		-2.26	**2.38E-02**				
ACR	PP	RadialMR - with outliers - IVW	651	64.0	-1.91	5.64E-02	727.0	0.0190		
ACR	PP	RadialMR - with outliers - Egger	651	64.0	0.40	6.89E-01	725.8	0.0204	-0.13	3.04E-01
ACR	PP	RadialMR - no outliers - IVW	603	61.5	-2.75	**5.91E-03**	453.6	1.0000		
ACR	PP	RadialMR - no outliers - Egger	603	61.5	-1.89	5.88E-02	452.6	1.0000	0.11	3.28E-01
ACR	PP	TSMR - WM	681		-1.52	1.28E-01				
ACR	PP	TSMR - IVW	681		-1.31	1.92E-01	777.1	0.0056		
ACR	PP	TSMR - Egger	681		0.06	9.51E-01	776.7	0.0054	0.00	5.50E-01
BCC	WMH	GRS	23		-2.20	**2.79E-02**				
BCC	WMH	GSMR	22		-1.34	1.79E-01				
BCC	WMH	RadialMR - with outliers - IVW	22	55.0	-2.82	**4.77E-03**	38.8	0.0103		
BCC	WMH	RadialMR - with outliers - Egger	22	55.0	-0.54	5.94E-01	38.8	0.0103	0.00	9.97E-01
BCC	WMH	RadialMR - no outliers - IVW	19	55.2	-1.56	1.18E-01	11.5	0.8705		
BCC	WMH	RadialMR - no outliers - Egger	19	55.2	0.13	9.01E-01	11.2	0.8849	-0.46	4.85E-01
BCC	WMH	TSMR - WM	22		-0.80	4.25E-01				
BCC	WMH	TSMR - IVW	22		-1.90	5.69E-02	38.1	0.0126		
BCC	WMH	TSMR - Egger	22		-0.67	5.13E-01	38.1	0.0087	0.00	9.67E-01
CgC	WMH	GRS	23		-2.32	**2.04E-02**				
CgC	WMH	GSMR	22		-1.50	1.33E-01				
CgC	WMH	RadialMR - with outliers - IVW	22	55.0	-2.87	**4.09E-03**	39.1	0.0097		
CgC	WMH	RadialMR - with outliers - Egger	22	55.0	-0.88	3.88E-01	38.4	0.0115	0.36	7.37E-01
CgC	WMH	RadialMR - no outliers - IVW	20	56.5	-2.26	**2.36E-02**	16.3	0.6340		
CgC	WMH	RadialMR - no outliers - Egger	20	56.5	-0.73	4.76E-01	16.3	0.6350	0.04	9.53E-01
CgC	WMH	TSMR - WM	22		-1.18	2.38E-01				
CgC	WMH	TSMR - IVW	22		-1.93	5.39E-02	38.1	0.0126		
CgC	WMH	TSMR - Egger	22		-0.89	3.86E-01	37.9	0.0090	0.01	7.92E-01
CP	HDL	GRS	513		2.00	**4.57E-02**				
CP	HDL	GSMR	691		2.70	**6.88E-03**				
CP	HDL	RadialMR - with outliers - IVW	484	252.7	1.90	5.81E-02	490.1	0.4022		
CP	HDL	RadialMR - with outliers - Egger	484	252.7	2.29	**2.23E-02**	487.9	0.4297	-0.11	1.45E-01
CP	HDL	RadialMR - no outliers - IVW	455	254.6	1.75	8.05E-02	324.4	1.0000		
CP	HDL	RadialMR - no outliers - Egger	455	254.6	1.19	2.33E-01	324.4	1.0000	0.00	9.60E-01
CP	HDL	TSMR - WM	549		1.49	1.35E-01				
CP	HDL	TSMR - IVW	549		1.83	6.66E-02	582.1	0.1519		
CP	HDL	TSMR - Egger	549		2.48	**1.34E-02**	579.0	0.1662	0.00	8.99E-02
CP	TG	GRS	490		-2.11	**3.51E-02**				
CP	TG	GSMR	680		-1.20	2.32E-01				
CP	TG	RadialMR - with outliers - IVW	463	192.0	-1.88	6.02E-02	462.6	0.4830		
CP	TG	RadialMR - with outliers - Egger	463	192.0	-2.41	**1.61E-02**	459.8	0.5200	0.14	9.81E-02
CP	TG	RadialMR - no outliers - IVW	439	193.6	-1.74	8.27E-02	338.4	0.9999		
CP	TG	RadialMR - no outliers - Egger	439	193.6	-2.39	**1.75E-02**	336.5	0.9999	0.12	1.21E-01
CP	TG	TSMR - WM	536		-2.96	**3.10E-03**				
CP	TG	TSMR - IVW	536		-1.96	5.02E-02	539.7	0.4351		
CP	TG	TSMR - Egger	536		-2.21	**2.72E-02**	538.1	0.4422	0.00	2.09E-01
CWM	WMH	GRS	23		-3.83	**1.28E-04**				
CWM	WMH	GSMR	22		-2.74	**6.17E-03**				
CWM	WMH	RadialMR - with outliers - IVW	22	55.0	-4.16	**3.15E-05**	53.4	0.0001		
CWM	WMH	RadialMR - with outliers - Egger	22	55.0	-0.72	4.80E-01	53.3	0.0001	0.05	9.65E-01
CWM	WMH	RadialMR - no outliers - IVW	19	55.2	-2.44	**1.48E-02**	13.9	0.7384		
CWM	WMH	RadialMR - no outliers - Egger	19	55.2	-0.19	8.54E-01	13.6	0.7543	-0.44	5.36E-01
CWM	WMH	TSMR - WM	22		-1.25	2.13E-01				
CWM	WMH	TSMR - IVW	22		-2.51	**1.22E-02**	51.4	0.0002		
CWM	WMH	TSMR - Egger	22		-0.76	4.54E-01	51.4	0.0001	0.00	9.49E-01

									Egger intercept	
ROI	Exposure	Methods	N SNPs	Mean F	Z	p	Q	p(Q)	Beta	p
CWM	TG	GRS	490		-2.06	**3.97E-02**				
CWM	TG	GSMR	677		-1.93	5.40E-02				
CWM	TG	RadialMR - with outliers - IVW	463	192.0	-2.65	**8.03E-03**	528.2	0.0178		
CWM	TG	RadialMR - with outliers - Egger	463	192.0	-2.04	**4.16E-02**	527.3	0.0189	0.07	4.12E-01
CWM	TG	RadialMR - no outliers - IVW	433	199.0	-2.88	**3.99E-03**	357.9	0.9961		
CWM	TG	RadialMR - no outliers - Egger	433	199.0	-2.17	**3.06E-02**	357.7	0.9962	0.04	6.11E-01
CWM	TG	TSMR - WM	536		-2.10	**3.57E-02**				
CWM	TG	TSMR - IVW	536		-2.44	**1.48E-02**	623.0	0.0050		
CWM	TG	TSMR - Egger	536		-2.14	**3.29E-02**	622.3	0.0049	0.00	4.42E-01
EC	WMH	GRS	23		-2.33	**1.99E-02**				
EC	WMH	GSMR	22		-1.80	7.23E-02				
EC	WMH	RadialMR - with outliers - IVW	22	55.0	-2.76	**5.79E-03**	37.1	0.0162		
EC	WMH	RadialMR - with outliers - Egger	22	55.0	-0.03	9.79E-01	36.8	0.0176	-0.57	5.92E-01
EC	WMH	RadialMR - no outliers - IVW	19	57.3	-1.70	8.98E-02	14.4	0.7021		
EC	WMH	RadialMR - no outliers - Egger	19	57.3	0.13	8.97E-01	14.0	0.7260	-0.50	4.98E-01
EC	WMH	TSMR - WM	22		-1.46	1.45E-01				
EC	WMH	TSMR - IVW	22		-2.17	**3.02E-02**	32.5	0.0518		
EC	WMH	TSMR - Egger	22		-0.09	9.26E-01	31.8	0.0453	-0.02	5.13E-01
EC	TG	GRS	490		-2.13	**3.33E-02**				
EC	TG	GSMR	676		-2.26	**2.40E-02**				
EC	TG	RadialMR - with outliers - IVW	463	192.0	-2.86	**4.29E-03**	533.2	0.0121		
EC	TG	RadialMR - with outliers - Egger	463	192.0	-2.61	**9.48E-03**	531.0	0.0144	0.12	1.70E-01
EC	TG	RadialMR - no outliers - IVW	428	184.9	-2.53	**1.14E-02**	325.7	0.9999		
EC	TG	RadialMR - no outliers - Egger	428	184.9	-2.57	**1.06E-02**	324.6	0.9999	0.09	2.36E-01
EC	TG	TSMR - WM	536		-2.12	**3.40E-02**				
EC	TG	TSMR - IVW	536		-2.69	**7.17E-03**	644.4	0.0008		
EC	TG	TSMR - Egger	536		-2.97	**3.16E-03**	641.3	0.0010	0.00	1.05E-01
FX	WMH	GRS	23		-1.99	**4.67E-02**				
FX	WMH	GSMR	23		-2.22	**2.67E-02**				
FX	WMH	RadialMR - with outliers - IVW	22	55.0	-2.14	**3.27E-02**	23.3	0.3269		
FX	WMH	RadialMR - with outliers - Egger	22	55.0	-1.14	2.66E-01	22.6	0.3645	0.50	5.42E-01
FX	WMH	RadialMR - no outliers - IVW	21	56.0	-2.56	**1.03E-02**	15.3	0.7610		
FX	WMH	RadialMR - no outliers - Egger	21	56.0	-0.90	3.78E-01	15.2	0.7630	0.08	9.10E-01
FX	WMH	TSMR - WM	22		-2.26	**2.41E-02**				
FX	WMH	TSMR - IVW	22		-1.94	5.19E-02	22.5	0.3684		
FX	WMH	TSMR - Egger	22		-1.06	3.04E-01	22.3	0.3226	0.01	6.66E-01
FX	WHR	GRS	525		-2.06	**3.93E-02**				
FX	WHR	GSMR	629		-1.98	**4.79E-02**				
FX	WHR	RadialMR - with outliers - IVW	511	69.3	-2.17	**3.02E-02**	511.6	0.4713		
FX	WHR	RadialMR - with outliers - Egger	511	69.3	-0.43	6.70E-01	511.6	0.4721	-0.04	7.40E-01
FX	WHR	RadialMR - no outliers - IVW	481	70.6	-2.25	**2.47E-02**	364.1	1.0000		
FX	WHR	RadialMR - no outliers - Egger	481	70.6	-0.93	3.50E-01	364.1	1.0000	0.00	9.65E-01
FX	WHR	TSMR - WM	542		-1.67	9.57E-02				
FX	WHR	TSMR - IVW	542		-2.34	**1.95E-02**	538.1	0.5272		
FX	WHR	TSMR - Egger	542		-0.01	9.94E-01	537.1	0.5268	0.00	3.27E-01
FX.ST	LDL	GRS	546		-2.02	**4.37E-02**				
FX.ST	LDL	GSMR	761		-1.14	2.56E-01				
FX.ST	LDL	RadialMR - with outliers - IVW	521	222.3	-1.18	2.38E-01	537.9	0.2847		
FX.ST	LDL	RadialMR - with outliers - Egger	521	222.3	-0.38	7.07E-01	537.7	0.2866	-0.03	6.74E-01
FX.ST	LDL	RadialMR - no outliers - IVW	491	213.2	-1.34	1.79E-01	370.0	1.0000		
FX.ST	LDL	RadialMR - no outliers - Egger	491	213.2	-0.97	3.33E-01	370.0	1.0000	0.00	9.52E-01
FX.ST	LDL	TSMR - WM	598		-1.11	2.65E-01				
FX.ST	LDL	TSMR - IVW	598		-1.81	7.00E-02	612.5	0.3209		
FX.ST	LDL	TSMR - Egger	598		-1.30	1.95E-01	612.5	0.3108	0.00	9.18E-01
FX.ST	BMI	GRS	1028		3.38	**7.24E-04**				
FX.ST	BMI	GSMR	1184		3.67	**2.42E-04**				
FX.ST	BMI	RadialMR - with outliers - IVW	1018	59.3	3.64	**2.68E-04**	1044.4	0.2688		
FX.ST	BMI	RadialMR - with outliers - Egger	1018	59.3	1.93	5.34E-02	1043.2	0.2773	-0.08	3.95E-01
FX.ST	BMI	RadialMR - no outliers - IVW	972	59.1	3.22	**1.29E-03**	759.8	1.0000		
FX.ST	BMI	RadialMR - no outliers - Egger	972	59.1	2.34	**1.92E-02**	758.1	1.0000	-0.11	2.07E-01
FX.ST	BMI	TSMR - WM	1043		1.72	8.54E-02				
FX.ST	BMI	TSMR - IVW	1043		3.21	**1.32E-03**	1083.1	0.1830		
FX.ST	BMI	TSMR - Egger	1043		2.11	**3.49E-02**	1081.9	0.1840	0.00	2.84E-01
GCC	WMH	GRS	23		-2.18	**2.93E-02**				
GCC	WMH	GSMR	22		-1.07	2.83E-01				
GCC	WMH	RadialMR - with outliers - IVW	22	55.0	-2.27	**2.30E-02**	32.0	0.0580		
GCC	WMH	RadialMR - with outliers - Egger	22	55.0	-0.59	5.61E-01	32.0	0.0590	0.10	9.17E-01
GCC	WMH	RadialMR - no outliers - IVW	19	55.2	-1.22	2.23E-01	13.7	0.7483		

									Egger intercept	
ROI	Exposure	Methods	N SNPs	Mean F	Z	p	Q	p(Q)	Beta	p
GCC	WMH	RadialMR - no outliers - Egger	19	55.2	0.15	8.84E-01	13.5	0.7636	-0.40	5.76E-01
GCC	WMH	TSMR - WM	22		-0.05	9.62E-01				
GCC	WMH	TSMR - IVW	22		-1.58	1.14E-01	31.6	0.0637		
GCC	WMH	TSMR - Egger	22		-0.85	4.04E-01	31.4	0.0496	0.01	7.29E-01
ICP	LDL	GRS	546		-2.25	**2.42E-02**				
ICP	LDL	GSMR	766		-2.40	**1.62E-02**				
ICP	LDL	RadialMR - with outliers - IVW	521	222.3	-0.82	4.13E-01	552.2	0.1592		
ICP	LDL	RadialMR - with outliers - Egger	521	222.3	-1.48	1.40E-01	550.5	0.1718	0.09	2.10E-01
ICP	LDL	RadialMR - no outliers - IVW	497	221.6	0.09	9.31E-01	425.6	0.9900		
ICP	LDL	RadialMR - no outliers - Egger	497	221.6	-0.69	4.90E-01	424.9	0.9907	0.06	3.42E-01
ICP	LDL	TSMR - WM	598		-0.65	5.15E-01				
ICP	LDL	TSMR - IVW	598		-1.52	1.27E-01	618.9	0.2595		
ICP	LDL	TSMR - Egger	598		-2.44	**1.50E-02**	615.1	0.2852	0.00	5.69E-02
ICP	BMI	GRS	1028		3.31	**9.48E-04**				
ICP	BMI	GSMR	1180		4.24	**2.25E-05**				
ICP	BMI	RadialMR - with outliers - IVW	1018	59.3	3.26	**1.11E-03**	1066.8	0.1355		
ICP	BMI	RadialMR - with outliers - Egger	1018	59.3	0.49	6.27E-01	1066.6	0.1362	0.05	5.91E-01
ICP	BMI	RadialMR - no outliers - IVW	952	59.0	3.10	**1.91E-03**	700.3	1.0000		
ICP	BMI	RadialMR - no outliers - Egger	952	59.0	0.53	5.97E-01	700.1	1.0000	0.06	5.21E-01
ICP	BMI	TSMR - WM	1043		1.49	1.37E-01				
ICP	BMI	TSMR - IVW	1043		3.02	**2.57E-03**	1088.7	0.1535		
ICP	BMI	TSMR - Egger	1043		-0.14	8.90E-01	1087.0	0.1566	0.00	2.11E-01
MCP	LDL	GRS	546		-1.97	**4.88E-02**				
MCP	LDL	GSMR	766		-2.11	**3.45E-02**				
MCP	LDL	RadialMR - with outliers - IVW	521	222.3	-0.84	4.03E-01	545.6	0.2110		
MCP	LDL	RadialMR - with outliers - Egger	521	222.3	-1.43	1.53E-01	544.2	0.2241	0.09	2.39E-01
MCP	LDL	RadialMR - no outliers - IVW	489	227.8	-0.29	7.72E-01	383.0	0.9999		
MCP	LDL	RadialMR - no outliers - Egger	489	227.8	-1.33	1.85E-01	381.3	0.9999	0.09	1.53E-01
MCP	LDL	TSMR - WM	598		-0.92	3.57E-01				
MCP	LDL	TSMR - IVW	598		-1.76	7.78E-02	590.5	0.5672		
MCP	LDL	TSMR - Egger	598		-2.32	**2.06E-02**	588.2	0.5827	0.00	1.26E-01
MCP	BMI	GRS	1028		3.07	**2.15E-03**				
MCP	BMI	GSMR	1185		3.27	**1.08E-03**				
MCP	BMI	RadialMR - with outliers - IVW	1018	59.3	2.60	**9.35E-03**	1075.6	0.0986		
MCP	BMI	RadialMR - with outliers - Egger	1018	59.3	0.31	7.56E-01	1075.4	0.0993	0.05	6.14E-01
MCP	BMI	RadialMR - no outliers - IVW	962	58.3	2.69	**7.21E-03**	772.2	1.0000		
MCP	BMI	RadialMR - no outliers - Egger	962	58.3	0.96	3.39E-01	772.2	1.0000	0.00	9.82E-01
MCP	BMI	TSMR - WM	1043		1.40	1.60E-01				
MCP	BMI	TSMR - IVW	1043		2.51	**1.21E-02**	1095.3	0.1224		
MCP	BMI	TSMR - Egger	1043		0.26	7.97E-01	1094.9	0.1199	0.00	5.20E-01
ML	LDL	GRS	546		-2.52	**1.17E-02**				
ML	LDL	GSMR	763		-2.05	**4.02E-02**				
ML	LDL	RadialMR - with outliers - IVW	521	222.3	-0.96	3.38E-01	580.9	0.0330		
ML	LDL	RadialMR - with outliers - Egger	521	222.3	-1.70	9.06E-02	578.5	0.0383	0.11	1.49E-01
ML	LDL	RadialMR - no outliers - IVW	489	228.0	-1.10	2.70E-01	399.8	0.9986		
ML	LDL	RadialMR - no outliers - Egger	489	228.0	-1.88	6.08E-02	398.1	0.9989	0.09	1.52E-01
ML	LDL	TSMR - WM	598		-1.68	9.21E-02				
ML	LDL	TSMR - IVW	598		-1.98	**4.78E-02**	663.7	0.0298		
ML	LDL	TSMR - Egger	598		-2.56	**1.06E-02**	660.7	0.0337	0.00	9.70E-02
ML	BMI	GRS	1028		2.31	**2.07E-02**				
ML	BMI	GSMR	1185		2.08	**3.77E-02**				
ML	BMI	RadialMR - with outliers - IVW	1018	59.3	1.98	**4.73E-02**	1034.4	0.3454		
ML	BMI	RadialMR - with outliers - Egger	1018	59.3	-0.28	7.83E-01	1033.5	0.3522	0.09	3.47E-01
ML	BMI	RadialMR - no outliers - IVW	960	59.2	1.68	9.32E-02	731.1	1.0000		
ML	BMI	RadialMR - no outliers - Egger	960	59.2	-0.70	4.85E-01	729.7	1.0000	0.12	1.70E-01
ML	BMI	TSMR - WM	1043		1.16	2.46E-01				
ML	BMI	TSMR - IVW	1043		1.79	7.41E-02	1064.3	0.3087		
ML	BMI	TSMR - Egger	1043		-1.06	2.90E-01	1061.1	0.3259	0.00	7.46E-02
PCR	WMH	GRS	23		-3.71	**2.03E-04**				
PCR	WMH	GSMR	22		-2.75	**5.98E-03**				
PCR	WMH	RadialMR - with outliers - IVW	22	55.0	-4.20	**2.66E-05**	47.6	0.0008		
PCR	WMH	RadialMR - with outliers - Egger	22	55.0	-0.85	4.08E-01	47.3	0.0009	0.14	9.07E-01
PCR	WMH	RadialMR - no outliers - IVW	19	55.2	-2.60	**9.32E-03**	13.1	0.7868		
PCR	WMH	RadialMR - no outliers - Egger	19	55.2	-0.46	6.50E-01	13.0	0.7917	-0.29	6.76E-01
PCR	WMH	TSMR - WM	22		-1.32	1.88E-01				
PCR	WMH	TSMR - IVW	22		-2.63	**8.45E-03**	48.1	0.0007		
PCR	WMH	TSMR - Egger	22		-0.81	4.26E-01	48.1	0.0004	0.00	9.55E-01
PCR	PP	GRS	666		-2.18	**2.96E-02**				

									Egger intercept	
ROI	Exposure	Methods	N SNPs	Mean F	Z	p	Q	p(Q)	Beta	p
PCR	PP	GSMR	788		-2.15	**3.16E-02**				
PCR	PP	RadialMR - with outliers - IVW	651	64.0	-2.19	**2.87E-02**	707.6	0.0581		
PCR	PP	RadialMR - with outliers - Egger	651	64.0	-0.97	3.32E-01	707.4	0.0587	0.04	7.47E-01
PCR	PP	RadialMR - no outliers - IVW	610	63.8	-3.58	**3.45E-04**	456.8	1.0000		
PCR	PP	RadialMR - no outliers - Egger	610	63.8	-1.48	1.40E-01	456.7	1.0000	0.02	8.57E-01
PCR	PP	TSMR - WM	681		-1.37	1.69E-01				
PCR	PP	TSMR - IVW	681		-1.82	6.89E-02	720.1	0.1392		
PCR	PP	TSMR - Egger	681		-0.96	3.37E-01	720.0	0.1339	0.00	7.66E-01
PCR	TG	GRS	490		-2.02	**4.30E-02**				
PCR	TG	GSMR	678		-2.32	**2.04E-02**				
PCR	TG	RadialMR - with outliers - IVW	463	192.0	-2.59	**9.57E-03**	507.9	0.0690		
PCR	TG	RadialMR - with outliers - Egger	463	192.0	-1.77	7.68E-02	507.6	0.0703	0.04	6.15E-01
PCR	TG	RadialMR - no outliers - IVW	434	191.5	-3.58	**3.41E-04**	332.4	0.9999		
PCR	TG	RadialMR - no outliers - Egger	434	191.5	-2.47	**1.38E-02**	332.4	0.9999	0.02	7.78E-01
PCR	TG	TSMR - WM	536		-1.68	9.24E-02				
PCR	TG	TSMR - IVW	536		-2.40	**1.65E-02**	609.5	0.0140		
PCR	TG	TSMR - Egger	536		-2.08	**3.77E-02**	608.9	0.0135	0.00	4.67E-01
PLIC	TG	GRS	490		-2.47	**1.36E-02**				
PLIC	TG	GSMR	681		-1.71	8.65E-02				
PLIC	TG	RadialMR - with outliers - IVW	463	192.0	-2.64	**8.33E-03**	473.2	0.3487		
PLIC	TG	RadialMR - with outliers - Egger	463	192.0	-3.10	**2.03E-03**	469.0	0.4005	0.17	4.59E-02
PLIC	TG	RadialMR - no outliers - IVW	433	195.5	-3.17	**1.50E-03**	321.9	1.0000		
PLIC	TG	RadialMR - no outliers - Egger	433	195.5	-3.53	**4.54E-04**	319.4	1.0000	0.13	7.39E-02
PLIC	TG	TSMR - WM	536		-2.31	**2.07E-02**				
PLIC	TG	TSMR - IVW	536		-2.71	**6.66E-03**	567.3	0.1612		
PLIC	TG	TSMR - Egger	536		-3.00	**2.81E-03**	564.4	0.1750	0.00	9.87E-02
PTR	WMH	GRS	23		-4.01	**6.15E-05**				
PTR	WMH	GSMR	22		-2.69	**7.19E-03**				
PTR	WMH	RadialMR - with outliers - IVW	22	55.0	-3.90	**9.58E-05**	63.9	0.0000		
PTR	WMH	RadialMR - with outliers - Egger	22	55.0	-0.86	4.00E-01	62.7	0.0000	0.40	7.71E-01
PTR	WMH	RadialMR - no outliers - IVW	18	56.6	-2.35	**1.90E-02**	13.8	0.6810		
PTR	WMH	RadialMR - no outliers - Egger	18	56.6	-0.16	8.72E-01	13.6	0.6978	-0.45	5.57E-01
PTR	WMH	TSMR - WM	22		-1.51	1.30E-01				
PTR	WMH	TSMR - IVW	22		-2.22	**2.66E-02**	62.7	0.0000		
PTR	WMH	TSMR - Egger	22		-0.86	4.02E-01	62.7	0.0000	0.01	8.94E-01
PTR	TG	GRS	490		-2.02	**4.32E-02**				
PTR	TG	GSMR	680		-1.56	1.20E-01				
PTR	TG	RadialMR - with outliers - IVW	463	192.0	-2.29	**2.21E-02**	519.6	0.0326		
PTR	TG	RadialMR - with outliers - Egger	463	192.0	-1.64	1.02E-01	519.3	0.0334	0.05	5.85E-01
PTR	TG	RadialMR - no outliers - IVW	435	198.1	-1.96	5.02E-02	362.1	0.9948		
PTR	TG	RadialMR - no outliers - Egger	435	198.1	-2.11	**3.56E-02**	361.1	0.9954	0.09	2.68E-01
PTR	TG	TSMR - WM	536		-1.89	5.82E-02				
PTR	TG	TSMR - IVW	536		-1.90	5.78E-02	604.9	0.0192		
PTR	TG	TSMR - Egger	536		-1.63	1.04E-01	604.6	0.0183	0.00	5.83E-01
RLIC	WMH	GRS	23		-2.23	**2.56E-02**				
RLIC	WMH	GSMR	22		-1.74	8.21E-02				
RLIC	WMH	RadialMR - with outliers - IVW	22	55.0	-2.74	**6.23E-03**	38.4	0.0116		
RLIC	WMH	RadialMR - with outliers - Egger	22	55.0	-0.67	5.12E-01	38.2	0.0122	0.15	8.89E-01
RLIC	WMH	RadialMR - no outliers - IVW	20	56.5	-2.00	**4.56E-02**	15.7	0.6804		
RLIC	WMH	RadialMR - no outliers - Egger	20	56.5	-0.50	6.24E-01	15.7	0.6800	-0.08	9.11E-01
RLIC	WMH	TSMR - WM	22		-1.43	1.52E-01				
RLIC	WMH	TSMR - IVW	22		-2.12	**3.38E-02**	35.4	0.0255		
RLIC	WMH	TSMR - Egger	22		-0.63	5.39E-01	35.4	0.0181	0.00	9.39E-01
RLIC	BMI	GRS	1028		2.73	**6.38E-03**				
RLIC	BMI	GSMR	1185		2.22	**2.63E-02**				
RLIC	BMI	RadialMR - with outliers - IVW	1018	59.3	2.33	**1.96E-02**	1066.3	0.1375		
RLIC	BMI	RadialMR - with outliers - Egger	1018	59.3	2.48	**1.31E-02**	1061.7	0.1609	-0.19	6.21E-02
RLIC	BMI	RadialMR - no outliers - IVW	965	57.6	1.49	1.37E-01	760.4	1.0000		
RLIC	BMI	RadialMR - no outliers - Egger	965	57.6	1.39	1.64E-01	759.5	1.0000	-0.09	3.41E-01
RLIC	BMI	TSMR - WM	1043		1.11	2.68E-01				
RLIC	BMI	TSMR - IVW	1043		2.11	**3.49E-02**	1090.5	0.1442		
RLIC	BMI	TSMR - Egger	1043		2.56	**1.07E-02**	1086.6	0.1589	-0.01	5.18E-02
SCC	TG	GRS	490		-2.22	**2.67E-02**				
SCC	TG	GSMR	677		-1.03	3.05E-01				
SCC	TG	RadialMR - with outliers - IVW	463	192.0	-2.76	**5.86E-03**	459.4	0.5259		
SCC	TG	RadialMR - with outliers - Egger	463	192.0	-2.47	**1.38E-02**	458.0	0.5438	0.10	2.54E-01
SCC	TG	RadialMR - no outliers - IVW	434	187.2	-2.93	**3.42E-03**	295.8	1.0000		
SCC	TG	RadialMR - no outliers - Egger	434	187.2	-3.40	**7.43E-04**	293.7	1.0000	0.12	8.32E-02

									Egger intercept	
ROI	Exposure	Methods	N SNPs	Mean F	Z	p	Q	p(Q)	Beta	p
SCC	TG	TSMR - WM	536		-2.52	**1.16E-02**				
SCC	TG	TSMR - IVW	536		-2.66	**7.77E-03**	553.7	0.2796		
SCC	TG	TSMR - Egger	536		-2.49	**1.30E-02**	552.5	0.2806	0.00	2.99E-01
SCP	LDL	GRS	546		-2.02	**4.34E-02**				
SCP	LDL	GSMR	765		-1.10	2.70E-01				
SCP	LDL	RadialMR - with outliers - IVW	521	222.3	-0.35	7.29E-01	549.0	0.1833		
SCP	LDL	RadialMR - with outliers - Egger	521	222.3	-0.76	4.50E-01	548.5	0.1875	0.05	4.87E-01
SCP	LDL	RadialMR - no outliers - IVW	498	223.2	-0.92	3.58E-01	421.6	0.9939		
SCP	LDL	RadialMR - no outliers - Egger	498	223.2	-0.28	7.79E-01	421.4	0.9939	-0.03	6.73E-01
SCP	LDL	TSMR - WM	598		-0.67	5.01E-01				
SCP	LDL	TSMR - IVW	598		-1.19	2.33E-01	641.0	0.1032		
SCP	LDL	TSMR - Egger	598		-1.48	1.38E-01	640.1	0.1026	0.00	3.59E-01
SCP	TG	GRS	490		-2.07	**3.87E-02**				
SCP	TG	GSMR	683		-2.27	**2.33E-02**				
SCP	TG	RadialMR - with outliers - IVW	463	192.0	-2.52	**1.19E-02**	498.4	0.1170		
SCP	TG	RadialMR - with outliers - Egger	463	192.0	-2.57	**1.04E-02**	495.9	0.1330	0.13	1.37E-01
SCP	TG	RadialMR - no outliers - IVW	438	190.3	-3.64	**2.74E-04**	361.4	0.9965		
SCP	TG	RadialMR - no outliers - Egger	438	190.3	-3.70	**2.44E-04**	358.7	0.9975	0.14	7.22E-02
SCP	TG	TSMR - WM	536		-1.79	7.42E-02				
SCP	TG	TSMR - IVW	536		-2.70	**7.04E-03**	567.5	0.1602		
SCP	TG	TSMR - Egger	536		-3.41	**6.98E-04**	562.4	0.1909	0.01	2.87E-02
SCP	BMI	GRS	1028		2.45	**1.45E-02**				
SCP	BMI	GSMR	1187		3.29	**1.01E-03**				
SCP	BMI	RadialMR - with outliers - IVW	1018	59.3	3.15	**1.63E-03**	1088.2	0.0597		
SCP	BMI	RadialMR - with outliers - Egger	1018	59.3	-0.03	9.73E-01	1087.2	0.0622	0.11	2.98E-01
SCP	BMI	RadialMR - no outliers - IVW	952	58.7	3.22	**1.28E-03**	737.3	1.0000		
SCP	BMI	RadialMR - no outliers - Egger	952	58.7	0.49	6.21E-01	737.0	1.0000	0.06	4.94E-01
SCP	BMI	TSMR - WM	1043		0.78	4.35E-01				
SCP	BMI	TSMR - IVW	1043		2.82	**4.84E-03**	1117.8	0.0508		
SCP	BMI	TSMR - Egger	1043		-0.47	6.39E-01	1115.3	0.0541	0.00	1.25E-01
SCR	WMH	GRS	23		-3.55	**3.84E-04**				
SCR	WMH	GSMR	22		-2.55	**1.08E-02**				
SCR	WMH	RadialMR - with outliers - IVW	22	55.0	-3.77	**1.62E-04**	41.5	0.0048		
SCR	WMH	RadialMR - with outliers - Egger	22	55.0	-0.71	4.86E-01	41.5	0.0049	0.00	9.97E-01
SCR	WMH	RadialMR - no outliers - IVW	19	55.2	-2.28	**2.26E-02**	12.4	0.8259		
SCR	WMH	RadialMR - no outliers - Egger	19	55.2	-0.14	8.88E-01	12.1	0.8402	-0.44	5.11E-01
SCR	WMH	TSMR - WM	22		-1.81	7.02E-02				
SCR	WMH	TSMR - IVW	22		-2.54	**1.12E-02**	39.7	0.0080		
SCR	WMH	TSMR - Egger	22		-0.79	4.42E-01	39.7	0.0054	0.00	9.58E-01
SCR	TG	GRS	490		-2.04	**4.14E-02**				
SCR	TG	GSMR	680		-2.33	**1.98E-02**				
SCR	TG	RadialMR - with outliers - IVW	463	192.0	-2.36	**1.82E-02**	502.6	0.0932		
SCR	TG	RadialMR - with outliers - Egger	463	192.0	-2.29	**2.24E-02**	500.8	0.1028	0.11	2.09E-01
SCR	TG	RadialMR - no outliers - IVW	434	198.3	-2.85	**4.43E-03**	346.2	0.9992		
SCR	TG	RadialMR - no outliers - Egger	434	198.3	-2.47	**1.40E-02**	345.6	0.9992	0.07	3.95E-01
SCR	TG	TSMR - WM	536		-1.35	1.78E-01				
SCR	TG	TSMR - IVW	536		-2.50	**1.24E-02**	607.7	0.0158		
SCR	TG	TSMR - Egger	536		-2.53	**1.16E-02**	606.0	0.0165	0.00	2.21E-01
SLF	WMH	GRS	23		-3.80	**1.47E-04**				
SLF	WMH	GSMR	22		-2.89	**3.87E-03**				
SLF	WMH	RadialMR - with outliers - IVW	22	55.0	-4.08	**4.51E-05**	45.6	0.0015		
SLF	WMH	RadialMR - with outliers - Egger	22	55.0	-0.84	4.12E-01	45.3	0.0016	0.14	9.06E-01
SLF	WMH	RadialMR - no outliers - IVW	19	55.2	-2.33	**1.97E-02**	10.9	0.8981		
SLF	WMH	RadialMR - no outliers - Egger	19	55.2	-0.44	6.68E-01	10.8	0.9013	-0.27	6.73E-01
SLF	WMH	TSMR - WM	22		-1.74	8.20E-02				
SLF	WMH	TSMR - IVW	22		-2.65	**8.12E-03**	46.2	0.0012		
SLF	WMH	TSMR - Egger	22		-0.75	4.63E-01	46.1	0.0008	0.00	8.98E-01
SLF	TG	GRS	490		-2.51	**1.22E-02**				
SLF	TG	GSMR	678		-2.10	**3.59E-02**				
SLF	TG	RadialMR - with outliers - IVW	463	192.0	-2.74	**6.13E-03**	501.9	0.0971		
SLF	TG	RadialMR - with outliers - Egger	463	192.0	-1.67	9.49E-02	501.8	0.0976	0.02	7.81E-01
SLF	TG	RadialMR - no outliers - IVW	434	192.7	-3.22	**1.30E-03**	338.6	0.9997		
SLF	TG	RadialMR - no outliers - Egger	434	192.7	-2.13	**3.39E-02**	338.5	0.9997	0.01	8.78E-01
SLF	TG	TSMR - WM	536		-1.13	2.59E-01				
SLF	TG	TSMR - IVW	536		-2.67	**7.51E-03**	593.3	0.0409		
SLF	TG	TSMR - Egger	536		-1.94	5.27E-02	593.1	0.0386	0.00	7.49E-01
SS	WMH	GRS	23		-3.69	**2.21E-04**				
SS	WMH	GSMR	21		-2.89	**3.84E-03**				

ROI	Exposure	Methods	N SNPs	Mean F	Z	p	Q	p(Q)	Egger intercept	
									Beta	p
SS	WMH	RadialMR - with outliers - IVW	22	55.0	-3.49	**4.86E-04**	49.8	0.0004		
SS	WMH	RadialMR - with outliers - Egger	22	55.0	-0.96	3.51E-01	48.7	0.0005	0.45	7.09E-01
SS	WMH	RadialMR - no outliers - IVW	18	55.5	-1.69	9.17E-02	13.7	0.6854		
SS	WMH	RadialMR - no outliers - Egger	18	55.5	-0.84	4.12E-01	13.6	0.6951	0.23	7.54E-01
SS	WMH	TSMR - WM	22		-1.26	2.08E-01				
SS	WMH	TSMR - IVW	22		-2.13	**3.33E-02**	50.1	0.0004		
SS	WMH	TSMR - Egger	22		-0.94	3.58E-01	49.9	0.0002	0.01	8.01E-01
SS	LDL	GRS	546		-2.32	**2.02E-02**				
SS	LDL	GSMR	762		-1.72	8.47E-02				
SS	LDL	RadialMR - with outliers - IVW	521	222.3	-1.26	2.09E-01	566.8	0.0765		
SS	LDL	RadialMR - with outliers - Egger	521	222.3	-0.72	4.69E-01	566.8	0.0765	0.00	9.92E-01
SS	LDL	RadialMR - no outliers - IVW	484	212.8	-1.69	9.08E-02	362.6	1.0000		
SS	LDL	RadialMR - no outliers - Egger	484	212.8	-1.63	1.04E-01	362.3	1.0000	0.04	5.53E-01
SS	LDL	TSMR - WM	598		-1.73	8.31E-02				
SS	LDL	TSMR - IVW	598		-2.18	**2.89E-02**	617.4	0.2737		
SS	LDL	TSMR - Egger	598		-1.99	**4.70E-02**	616.8	0.2689	0.00	4.84E-01
TAP	WMH	GRS	23		-2.79	**5.34E-03**				
TAP	WMH	GSMR	22		-1.88	5.96E-02				
TAP	WMH	RadialMR - with outliers - IVW	22	55.0	-2.96	**3.12E-03**	49.3	0.0005		
TAP	WMH	RadialMR - with outliers - Egger	22	55.0	0.15	8.79E-01	48.5	0.0006	-0.82	4.96E-01
TAP	WMH	RadialMR - no outliers - IVW	18	56.8	-1.13	2.58E-01	14.9	0.6043		
TAP	WMH	RadialMR - no outliers - Egger	18	56.8	0.39	7.03E-01	14.3	0.6437	-0.58	4.46E-01
TAP	WMH	TSMR - WM	22		-0.32	7.52E-01				
TAP	WMH	TSMR - IVW	22		-1.79	7.37E-02	49.4	0.0004		
TAP	WMH	TSMR - Egger	22		0.01	9.96E-01	48.4	0.0004	-0.02	5.32E-01
UNC	PP	GRS	666		-1.99	**4.66E-02**				
UNC	PP	GSMR	792		-1.26	2.08E-01				
UNC	PP	RadialMR - with outliers - IVW	651	64.0	-1.73	8.43E-02	640.2	0.6008		
UNC	PP	RadialMR - with outliers - Egger	651	64.0	-0.10	9.22E-01	640.0	0.6028	-0.06	6.30E-01
UNC	PP	RadialMR - no outliers - IVW	618	64.0	-2.60	**9.36E-03**	456.3	1.0000		
UNC	PP	RadialMR - no outliers - Egger	618	64.0	0.39	6.98E-01	454.8	1.0000	-0.15	1.52E-01
UNC	PP	TSMR - WM	681		-0.50	6.17E-01				
UNC	PP	TSMR - IVW	681		-1.30	1.94E-01	685.8	0.4311		
UNC	PP	TSMR - Egger	681		0.05	9.62E-01	685.4	0.4240	0.00	5.63E-01
UNC	HDL	GRS	513		3.21	**1.31E-03**				
UNC	HDL	GSMR	691		3.99	**6.49E-05**				
UNC	HDL	RadialMR - with outliers - IVW	484	252.7	3.34	**8.26E-04**	486.6	0.4451		
UNC	HDL	RadialMR - with outliers - Egger	484	252.7	1.82	6.97E-02	486.6	0.4455	0.02	8.36E-01
UNC	HDL	RadialMR - no outliers - IVW	458	248.9	2.69	**7.07E-03**	351.4	0.9999		
UNC	HDL	RadialMR - no outliers - Egger	458	248.9	0.48	6.32E-01	349.2	0.9999	0.12	8.77E-02
UNC	HDL	TSMR - WM	549		1.01	3.12E-01				
UNC	HDL	TSMR - IVW	549		3.50	**4.63E-04**	557.2	0.3839		
UNC	HDL	TSMR - Egger	549		2.41	**1.65E-02**	557.1	0.3730	0.00	8.31E-01
UNC	LDL	GRS	546		-3.28	**1.04E-03**				
UNC	LDL	GSMR	761		-2.52	**1.16E-02**				
UNC	LDL	RadialMR - with outliers - IVW	521	222.3	-2.23	**2.58E-02**	544.9	0.2170		
UNC	LDL	RadialMR - with outliers - Egger	521	222.3	-1.02	3.06E-01	544.8	0.2183	-0.03	7.02E-01
UNC	LDL	RadialMR - no outliers - IVW	488	224.8	-1.70	8.87E-02	365.3	1.0000		
UNC	LDL	RadialMR - no outliers - Egger	488	224.8	-0.82	4.13E-01	365.2	1.0000	-0.03	6.31E-01
UNC	LDL	TSMR - WM	598		-1.61	1.08E-01				
UNC	LDL	TSMR - IVW	598		-1.97	**4.84E-02**	615.2	0.2939		
UNC	LDL	TSMR - Egger	598		0.10	9.19E-01	611.4	0.3227	-0.01	5.20E-02
UNC	TG	GRS	490		-2.47	**1.35E-02**				
UNC	TG	GSMR	677		-2.48	**1.30E-02**				
UNC	TG	RadialMR - with outliers - IVW	463	192.0	-2.52	**1.18E-02**	544.8	0.0047		
UNC	TG	RadialMR - with outliers - Egger	463	192.0	-2.61	**9.37E-03**	541.7	0.0061	0.15	1.10E-01
UNC	TG	RadialMR - no outliers - IVW	426	186.5	-1.93	5.38E-02	336.4	0.9994		
UNC	TG	RadialMR - no outliers - Egger	426	186.5	-2.49	**1.32E-02**	334.5	0.9996	0.12	1.19E-01
UNC	TG	TSMR - WM	536		-3.21	**1.32E-03**				
UNC	TG	TSMR - IVW	536		-2.61	**9.17E-03**	602.3	0.0229		
UNC	TG	TSMR - Egger	536		-3.35	**8.78E-04**	596.9	0.0304	0.01	2.95E-02
**ODI**										
CgH	T2D	GRS	241		-2.00	**4.56E-02**				
CgH	T2D	GSMR	280		-1.38	1.67E-01				
CgH	T2D	RadialMR - with outliers - IVW	243	72.0	-1.23	2.17E-01	233.2	0.6465		
CgH	T2D	RadialMR - with outliers - Egger	243	72.0	0.40	6.91E-01	232.2	0.6632	-0.15	3.14E-01
CgH	T2D	RadialMR - no outliers - IVW	230	72.5	-0.86	3.87E-01	159.8	0.9998		
CgH	T2D	RadialMR - no outliers - Egger	230	72.5	0.79	4.28E-01	158.5	0.9999	-0.18	1.76E-01

									Egger intercept	
ROI	Exposure	Methods	N SNPs	Mean F	Z	p	Q	p(Q)	Beta	p
CgH	T2D	TSMR - WM	263		-0.33	7.40E-01				
CgH	T2D	TSMR - IVW	263		-1.41	1.58E-01	255.9	0.5944		
CgH	T2D	TSMR - Egger	263		0.95	3.42E-01	252.8	0.6313	-0.01	7.72E-02
CgH	BMI	GRS	1028		2.21	**2.73E-02**				
CgH	BMI	GSMR	1186		2.71	**6.70E-03**				
CgH	BMI	RadialMR - with outliers - IVW	1018	59.3	2.48	**1.30E-02**	1021.6	0.4534		
CgH	BMI	RadialMR - with outliers - Egger	1018	59.3	0.97	3.34E-01	1021.5	0.4543	-0.02	8.42E-01
CgH	BMI	RadialMR - no outliers - IVW	972	59.2	2.58	**9.90E-03**	764.7	1.0000		
CgH	BMI	RadialMR - no outliers - Egger	972	59.2	1.72	8.55E-02	763.9	1.0000	-0.08	3.94E-01
CgH	BMI	TSMR - WM	1043		2.29	**2.19E-02**				
CgH	BMI	TSMR - IVW	1043		2.31	**2.08E-02**	1060.5	0.3386		
CgH	BMI	TSMR - Egger	1043		0.37	7.13E-01	1060.2	0.3323	0.00	6.49E-01
CST	BMI	GRS	1028		2.55	**1.09E-02**				
CST	BMI	GSMR	1186		2.93	**3.42E-03**				
CST	BMI	RadialMR - with outliers - IVW	1018	59.3	2.68	**7.26E-03**	1126.6	0.0091		
CST	BMI	RadialMR - with outliers - Egger	1018	59.3	-0.29	7.70E-01	1125.3	0.0098	0.12	2.50E-01
CST	BMI	RadialMR - no outliers - IVW	960	59.3	1.84	6.58E-02	789.5	1.0000		
CST	BMI	RadialMR - no outliers - Egger	960	59.3	0.36	7.17E-01	789.5	1.0000	0.03	7.72E-01
CST	BMI	TSMR - WM	1043		0.42	6.74E-01				
CST	BMI	TSMR - IVW	1043		2.47	**1.36E-02**	1154.4	0.0083		
CST	BMI	TSMR - Egger	1043		-0.45	6.53E-01	1152.3	0.0088	0.00	1.66E-01
CWM	BMI	GRS	1028		2.20	**2.79E-02**				
CWM	BMI	GSMR	1184		2.81	**5.01E-03**				
CWM	BMI	RadialMR - with outliers - IVW	1018	59.3	2.32	**2.02E-02**	1003.1	0.6162		
CWM	BMI	RadialMR - with outliers - Egger	1018	59.3	0.78	4.35E-01	1003.1	0.6163	0.00	9.60E-01
CWM	BMI	RadialMR - no outliers - IVW	972	59.0	2.22	**2.67E-02**	722.0	1.0000		
CWM	BMI	RadialMR - no outliers - Egger	972	59.0	0.18	8.60E-01	721.7	1.0000	0.06	5.12E-01
CWM	BMI	TSMR - WM	1043		1.26	2.07E-01				
CWM	BMI	TSMR - IVW	1043		2.23	**2.60E-02**	1029.2	0.6053		
CWM	BMI	TSMR - Egger	1043		0.49	6.21E-01	1029.1	0.5975	0.00	7.72E-01
FX	WHR	GRS	525		2.54	**1.11E-02**				
FX	WHR	GSMR	628		2.61	**9.00E-03**				
FX	WHR	RadialMR - with outliers - IVW	511	69.3	3.43	**5.93E-04**	514.9	0.4311		
FX	WHR	RadialMR - with outliers - Egger	511	69.3	0.86	3.89E-01	514.9	0.4315	0.04	7.45E-01
FX	WHR	RadialMR - no outliers - IVW	483	69.3	3.31	**9.29E-04**	353.8	1.0000		
FX	WHR	RadialMR - no outliers - Egger	483	69.3	1.28	2.00E-01	353.8	1.0000	0.01	9.60E-01
FX	WHR	TSMR - WM	542		2.21	**2.74E-02**				
FX	WHR	TSMR - IVW	542		3.28	**1.03E-03**	537.7	0.5319		
FX	WHR	TSMR - Egger	542		1.10	2.70E-01	537.7	0.5202	0.00	8.48E-01
FX.ST	WMH	GRS	23		2.01	**4.46E-02**				
FX.ST	WMH	GSMR	23		1.24	2.14E-01				
FX.ST	WMH	RadialMR - with outliers - IVW	22	55.0	1.46	1.44E-01	14.1	0.8644		
FX.ST	WMH	RadialMR - with outliers - Egger	22	55.0	-0.34	7.37E-01	13.6	0.8853	0.54	3.96E-01
FX.ST	WMH	RadialMR - no outliers - IVW	22	55.0	1.46	1.44E-01	14.1	0.8644		
FX.ST	WMH	RadialMR - no outliers - Egger	22	55.0	-0.34	7.37E-01	13.6	0.8853	0.54	3.96E-01
FX.ST	WMH	TSMR - WM	22		1.06	2.91E-01				
FX.ST	WMH	TSMR - IVW	22		1.32	1.87E-01	14.7	0.8374		
FX.ST	WMH	TSMR - Egger	22		0.04	9.68E-01	14.5	0.8031	0.01	6.72E-01
GCC	T2D	GRS	241		-2.08	**3.78E-02**				
GCC	T2D	GSMR	282		-2.05	**3.99E-02**				
GCC	T2D	RadialMR - with outliers - IVW	243	72.0	-2.31	**2.08E-02**	272.6	0.0859		
GCC	T2D	RadialMR - with outliers - Egger	243	72.0	-1.26	2.08E-01	272.3	0.0877	0.07	6.85E-01
GCC	T2D	RadialMR - no outliers - IVW	229	71.8	-1.36	1.74E-01	199.8	0.9114		
GCC	T2D	RadialMR - no outliers - Egger	229	71.8	-0.63	5.29E-01	199.8	0.9114	0.00	9.75E-01
GCC	T2D	TSMR - WM	263		-2.22	**2.61E-02**				
GCC	T2D	TSMR - IVW	263		-2.58	**9.90E-03**	287.6	0.1330		
GCC	T2D	TSMR - Egger	263		-0.40	6.90E-01	286.8	0.1307	0.00	3.97E-01
ICP	BMI	GRS	1028		2.41	**1.59E-02**				
ICP	BMI	GSMR	1185		2.36	**1.84E-02**				
ICP	BMI	RadialMR - with outliers - IVW	1018	59.3	2.02	**4.35E-02**	989.7	0.7244		
ICP	BMI	RadialMR - with outliers - Egger	1018	59.3	-0.44	6.62E-01	988.5	0.7336	0.11	2.55E-01
ICP	BMI	RadialMR - no outliers - IVW	973	58.8	2.70	**6.92E-03**	720.0	1.0000		
ICP	BMI	RadialMR - no outliers - Egger	973	58.8	-0.14	8.89E-01	719.0	1.0000	0.10	2.36E-01
ICP	BMI	TSMR - WM	1043		0.51	6.14E-01				
ICP	BMI	TSMR - IVW	1043		1.77	7.71E-02	1029.5	0.6029		
ICP	BMI	TSMR - Egger	1043		-0.70	4.86E-01	1027.5	0.6109	0.00	1.64E-01
MCP	BMI	GRS	1028		2.17	**2.97E-02**				
MCP	BMI	GSMR	1185		2.64	**8.27E-03**				

									Egger intercept	
ROI	Exposure	Methods	N SNPs	Mean F	Z	p	Q	p(Q)	Beta	p
MCP	BMI	RadialMR - with outliers - IVW	1018	59.3	2.75	**5.87E-03**	1095.4	0.0436		
MCP	BMI	RadialMR - with outliers - Egger	1018	59.3	-0.49	6.23E-01	1093.4	0.0476	0.14	1.63E-01
MCP	BMI	RadialMR - no outliers - IVW	960	59.7	1.36	1.74E-01	767.0	1.0000		
MCP	BMI	RadialMR - no outliers - Egger	960	59.7	-1.03	3.04E-01	764.8	1.0000	0.14	1.10E-01
MCP	BMI	TSMR - WM	1043		1.07	2.83E-01				
MCP	BMI	TSMR - IVW	1043		2.42	**1.54E-02**	1147.3	0.0123		
MCP	BMI	TSMR - Egger	1043		-1.53	1.27E-01	1140.4	0.0167	0.01	1.20E-02
ML	BMI	GRS	1028		3.35	**8.07E-04**				
ML	BMI	GSMR	1190		4.20	**2.66E-05**				
ML	BMI	RadialMR - with outliers - IVW	1018	59.3	3.64	**2.74E-04**	1034.8	0.3421		
ML	BMI	RadialMR - with outliers - Egger	1018	59.3	0.03	9.72E-01	1033.6	0.3513	0.11	2.47E-01
ML	BMI	RadialMR - no outliers - IVW	963	59.3	2.77	**5.61E-03**	731.9	1.0000		
ML	BMI	RadialMR - no outliers - Egger	963	59.3	0.34	7.31E-01	731.7	1.0000	0.06	4.85E-01
ML	BMI	TSMR - WM	1043		1.62	1.05E-01				
ML	BMI	TSMR - IVW	1043		3.10	**1.94E-03**	1091.9	0.1376		
ML	BMI	TSMR - Egger	1043		-0.78	4.33E-01	1087.8	0.1524	0.01	4.86E-02
PCR	WMH	GRS	23		-2.19	**2.82E-02**				
PCR	WMH	GSMR	23		-1.62	1.04E-01				
PCR	WMH	RadialMR - with outliers - IVW	22	55.0	-2.42	**1.56E-02**	24.8	0.2567		
PCR	WMH	RadialMR - with outliers - Egger	22	55.0	-1.04	3.10E-01	24.3	0.2788	0.39	6.50E-01
PCR	WMH	RadialMR - no outliers - IVW	21	55.6	-1.93	5.41E-02	19.8	0.4734		
PCR	WMH	RadialMR - no outliers - Egger	21	55.6	-1.25	2.25E-01	18.9	0.5261	0.57	4.63E-01
PCR	WMH	TSMR - WM	22		-1.73	8.36E-02				
PCR	WMH	TSMR - IVW	22		-2.21	**2.70E-02**	24.8	0.2578		
PCR	WMH	TSMR - Egger	22		-1.24	2.28E-01	24.4	0.2255	0.01	5.92E-01
PCR	HDL	GRS	513		-2.14	**3.24E-02**				
PCR	HDL	GSMR	688		-1.21	2.25E-01				
PCR	HDL	RadialMR - with outliers - IVW	484	252.7	-1.74	8.15E-02	564.5	0.0061		
PCR	HDL	RadialMR - with outliers - Egger	484	252.7	-2.53	**1.18E-02**	559.9	0.0087	0.16	5.14E-02
PCR	HDL	RadialMR - no outliers - IVW	456	243.8	-0.46	6.43E-01	386.6	0.9911		
PCR	HDL	RadialMR - no outliers - Egger	456	243.8	-0.94	3.49E-01	386.1	0.9915	0.06	4.28E-01
PCR	HDL	TSMR - WM	549		-0.48	6.32E-01				
PCR	HDL	TSMR - IVW	549		-1.34	1.80E-01	590.0	0.1041		
PCR	HDL	TSMR - Egger	549		-1.73	8.45E-02	588.6	0.1059	0.00	2.58E-01
PCT	BMI	GRS	1028		2.96	**3.06E-03**				
PCT	BMI	GSMR	1187		3.84	**1.23E-04**				
PCT	BMI	RadialMR - with outliers - IVW	1018	59.3	2.91	**3.63E-03**	1078.7	0.0875		
PCT	BMI	RadialMR - with outliers - Egger	1018	59.3	0.54	5.91E-01	1078.7	0.0876	0.04	7.16E-01
PCT	BMI	RadialMR - no outliers - IVW	961	59.5	1.92	5.51E-02	749.2	1.0000		
PCT	BMI	RadialMR - no outliers - Egger	961	59.5	0.83	4.09E-01	749.2	1.0000	-0.01	8.81E-01
PCT	BMI	TSMR - WM	1043		2.06	**3.93E-02**				
PCT	BMI	TSMR - IVW	1043		2.66	**7.86E-03**	1111.3	0.0669		
PCT	BMI	TSMR - Egger	1043		-0.23	8.19E-01	1109.7	0.0684	0.00	2.23E-01
PTR	BMI	GRS	1028		2.35	**1.86E-02**				
PTR	BMI	GSMR	1182		3.10	**1.96E-03**				
PTR	BMI	RadialMR - with outliers - IVW	1018	59.3	2.88	**4.02E-03**	1120.2	0.0129		
PTR	BMI	RadialMR - with outliers - Egger	1018	59.3	-0.19	8.52E-01	1119.0	0.0137	0.11	2.71E-01
PTR	BMI	RadialMR - no outliers - IVW	946	58.8	3.53	**4.21E-04**	737.6	1.0000		
PTR	BMI	RadialMR - no outliers - Egger	946	58.8	-0.07	9.42E-01	736.3	1.0000	0.13	1.63E-01
PTR	BMI	TSMR - WM	1043		1.37	1.70E-01				
PTR	BMI	TSMR - IVW	1043		2.61	**8.96E-03**	1129.6	0.0299		
PTR	BMI	TSMR - Egger	1043		-0.61	5.40E-01	1126.8	0.0325	0.01	1.07E-01
RLIC	BMI	GRS	1028		2.22	**2.62E-02**				
RLIC	BMI	GSMR	1182		3.11	**1.87E-03**				
RLIC	BMI	RadialMR - with outliers - IVW	1018	59.3	1.98	**4.78E-02**	1020.3	0.4654		
RLIC	BMI	RadialMR - with outliers - Egger	1018	59.3	0.90	3.68E-01	1020.1	0.4668	-0.03	7.67E-01
RLIC	BMI	RadialMR - no outliers - IVW	968	59.6	2.43	**1.52E-02**	740.4	1.0000		
RLIC	BMI	RadialMR - no outliers - Egger	968	59.6	1.25	2.13E-01	740.2	1.0000	-0.03	7.00E-01
RLIC	BMI	TSMR - WM	1043		1.34	1.82E-01				
RLIC	BMI	TSMR - IVW	1043		1.60	1.10E-01	1048.4	0.4386		
RLIC	BMI	TSMR - Egger	1043		0.59	5.54E-01	1048.4	0.4300	0.00	9.66E-01
SCC	BMI	GRS	1028		2.80	**5.18E-03**				
SCC	BMI	GSMR	1185		3.09	**2.01E-03**				
SCC	BMI	RadialMR - with outliers - IVW	1018	59.3	3.04	**2.39E-03**	1056.5	0.1896		
SCC	BMI	RadialMR - with outliers - Egger	1018	59.3	1.84	6.64E-02	1055.1	0.1979	-0.10	3.40E-01
SCC	BMI	RadialMR - no outliers - IVW	977	59.6	3.95	**7.83E-05**	808.2	1.0000		
SCC	BMI	RadialMR - no outliers - Egger	977	59.6	1.71	8.84E-02	807.9	1.0000	-0.03	7.27E-01
SCC	BMI	TSMR - WM	1043		1.67	9.57E-02				

ROI	Exposure	Methods	N SNPs	Mean F	Z	p	Q	p(Q)	Egger intercept Beta	Egger intercept p
SCC	BMI	TSMR - IVW	1043		2.91	**3.58E-03**	1075.7	0.2284		
SCC	BMI	TSMR - Egger	1043		1.33	1.85E-01	1075.5	0.2227	0.00	7.31E-01
SCP	LDL	GRS	546		-2.02	**4.30E-02**				
SCP	LDL	GSMR	763		-0.23	8.19E-01				
SCP	LDL	RadialMR - with outliers - IVW	521	222.3	-1.02	3.08E-01	483.8	0.8703		
SCP	LDL	RadialMR - with outliers - Egger	521	222.3	-0.08	9.35E-01	483.4	0.8735	-0.05	4.76E-01
SCP	LDL	RadialMR - no outliers - IVW	502	223.0	-1.56	1.19E-01	381.6	1.0000		
SCP	LDL	RadialMR - no outliers - Egger	502	223.0	-0.36	7.22E-01	380.9	1.0000	-0.06	3.49E-01
SCP	LDL	TSMR - WM	598		-0.48	6.32E-01				
SCP	LDL	TSMR - IVW	598		-1.20	2.32E-01	541.9	0.9482		
SCP	LDL	TSMR - Egger	598		-0.38	7.03E-01	541.5	0.9461	0.00	5.65E-01
SCP	BMI	GRS	1028		2.65	**8.03E-03**				
SCP	BMI	GSMR	1185		1.95	5.13E-02				
SCP	BMI	RadialMR - with outliers - IVW	1018	59.3	2.45	**1.43E-02**	1136.2	0.0052		
SCP	BMI	RadialMR - with outliers - Egger	1018	59.3	0.23	8.16E-01	1136.0	0.0053	0.05	6.04E-01
SCP	BMI	RadialMR - no outliers - IVW	949	59.9	1.52	1.29E-01	759.8	1.0000		
SCP	BMI	RadialMR - no outliers - Egger	949	59.9	0.57	5.70E-01	759.8	1.0000	0.00	9.77E-01
SCP	BMI	TSMR - WM	1043		0.85	3.94E-01				
SCP	BMI	TSMR - IVW	1043		2.21	**2.74E-02**	1140.3	0.0177		
SCP	BMI	TSMR - Egger	1043		-0.52	6.05E-01	1138.3	0.0186	0.00	1.74E-01
SFO	PP	GRS	666		-2.08	**3.74E-02**				
SFO	PP	GSMR	792		-1.76	7.84E-02				
SFO	PP	RadialMR - with outliers - IVW	651	64.0	-1.81	7.10E-02	636.0	0.6458		
SFO	PP	RadialMR - with outliers - Egger	651	64.0	0.47	6.40E-01	634.8	0.6581	-0.13	2.67E-01
SFO	PP	RadialMR - no outliers - IVW	618	63.5	-2.26	**2.39E-02**	466.7	1.0000		
SFO	PP	RadialMR - no outliers - Egger	618	63.5	-0.65	5.14E-01	466.7	1.0000	-0.02	8.57E-01
SFO	PP	TSMR - WM	681		-0.23	8.20E-01				
SFO	PP	TSMR - IVW	681		-1.70	8.94E-02	677.1	0.5246		
SFO	PP	TSMR - Egger	681		0.33	7.41E-01	676.0	0.5257	0.00	2.95E-01
SS	BMI	GRS	1028		1.99	**4.63E-02**				
SS	BMI	GSMR	1181		2.40	**1.66E-02**				
SS	BMI	RadialMR - with outliers - IVW	1018	59.3	2.19	**2.83E-02**	1065.4	0.1418		
SS	BMI	RadialMR - with outliers - Egger	1018	59.3	0.30	7.65E-01	1065.3	0.1423	0.04	6.95E-01
SS	BMI	RadialMR - no outliers - IVW	951	59.0	2.07	**3.86E-02**	689.7	1.0000		
SS	BMI	RadialMR - no outliers - Egger	951	59.0	0.29	7.73E-01	689.6	1.0000	0.04	6.13E-01
SS	BMI	TSMR - WM	1043		0.96	3.38E-01				
SS	BMI	TSMR - IVW	1043		1.84	6.57E-02	1111.5	0.0664		
SS	BMI	TSMR - Egger	1043		-0.03	9.76E-01	1110.9	0.0650	0.00	4.80E-01
UNC	T2D	GRS	241		-2.59	**9.58E-03**				
UNC	T2D	GSMR	282		-2.32	**2.02E-02**				
UNC	T2D	RadialMR - with outliers - IVW	243	72.0	-2.51	**1.19E-02**	215.8	0.8864		
UNC	T2D	RadialMR - with outliers - Egger	243	72.0	-0.56	5.77E-01	215.5	0.8888	-0.09	5.53E-01
UNC	T2D	RadialMR - no outliers - IVW	235	72.9	-2.33	**1.98E-02**	169.7	0.9995		
UNC	T2D	RadialMR - no outliers - Egger	235	72.9	-1.03	3.06E-01	169.7	0.9995	-0.02	9.03E-01
UNC	T2D	TSMR - WM	263		-2.00	**4.58E-02**				
UNC	T2D	TSMR - IVW	263		-2.54	**1.12E-02**	241.8	0.8095		
UNC	T2D	TSMR - Egger	263		-0.65	5.19E-01	241.5	0.8012	0.00	5.81E-01
**ISOVF**										
ALIC	WMH	GRS	23		2.89	**3.88E-03**				
ALIC	WMH	GSMR	23		2.46	**1.40E-02**				
ALIC	WMH	RadialMR - with outliers - IVW	22	55.0	2.64	**8.20E-03**	29.9	0.0935		
ALIC	WMH	RadialMR - with outliers - Egger	22	55.0	2.33	**3.02E-02**	24.1	0.2871	-1.49	9.12E-02
ALIC	WMH	RadialMR - no outliers - IVW	20	56.2	2.31	**2.07E-02**	13.9	0.7897		
ALIC	WMH	RadialMR - no outliers - Egger	20	56.2	3.01	**7.54E-03**	10.3	0.9449	-1.32	3.68E-02
ALIC	WMH	TSMR - WM	22		2.03	**4.27E-02**				
ALIC	WMH	TSMR - IVW	22		2.16	**3.05E-02**	31.3	0.0692		
ALIC	WMH	TSMR - Egger	22		2.43	**2.45E-02**	27.0	0.1342	-0.04	9.20E-02
BCC	WMH	GRS	23		2.99	**2.78E-03**				
BCC	WMH	GSMR	23		2.77	**5.55E-03**				
BCC	WMH	RadialMR - with outliers - IVW	22	55.0	2.79	**5.25E-03**	15.2	0.8107		
BCC	WMH	RadialMR - with outliers - Egger	22	55.0	1.76	9.30E-02	14.4	0.8532	-0.59	3.77E-01
BCC	WMH	RadialMR - no outliers - IVW	21	55.4	2.26	**2.41E-02**	10.7	0.9532		
BCC	WMH	RadialMR - no outliers - Egger	21	55.4	1.97	6.32E-02	9.9	0.9707	-0.63	2.67E-01
BCC	WMH	TSMR - WM	22		1.96	5.01E-02				
BCC	WMH	TSMR - IVW	22		2.90	**3.76E-03**	15.8	0.7808		
BCC	WMH	TSMR - Egger	22		1.21	2.42E-01	15.7	0.7326	-0.01	8.10E-01
CgC	WHR	GRS	525		-2.01	**4.45E-02**				
CgC	WHR	GSMR	628		-1.61	1.07E-01				

									Egger intercept	
ROI	Exposure	Methods	N SNPs	Mean F	Z	p	Q	p(Q)	Beta	p
CgC	WHR	RadialMR - with outliers - IVW	511	69.3	-2.04	**4.17E-02**	459.7	0.9462		
CgC	WHR	RadialMR - with outliers - Egger	511	69.3	-2.31	**2.14E-02**	456.7	0.9564	0.20	9.43E-02
CgC	WHR	RadialMR - no outliers - IVW	488	69.4	-1.21	2.25E-01	332.3	1.0000		
CgC	WHR	RadialMR - no outliers - Egger	488	69.4	-1.96	5.07E-02	330.5	1.0000	0.17	1.22E-01
CgC	WHR	TSMR - WM	542		-2.27	**2.35E-02**				
CgC	WHR	TSMR - IVW	542		-2.00	**4.56E-02**	482.8	0.9653		
CgC	WHR	TSMR - Egger	542		-1.90	5.77E-02	481.3	0.9667	0.00	2.23E-01
CST	BMI	GRS	1028		-2.46	**1.37E-02**				
CST	BMI	GSMR	1186		-2.43	**1.50E-02**				
CST	BMI	RadialMR - with outliers - IVW	1018	59.3	-1.77	7.69E-02	1112.2	0.0196		
CST	BMI	RadialMR - with outliers - Egger	1018	59.3	0.57	5.69E-01	1110.7	0.0211	-0.12	2.46E-01
CST	BMI	RadialMR - no outliers - IVW	951	58.8	-1.40	1.61E-01	736.9	1.0000		
CST	BMI	RadialMR - no outliers - Egger	951	58.8	0.49	6.28E-01	736.0	1.0000	-0.09	2.96E-01
CST	BMI	TSMR - WM	1043		-0.95	3.41E-01				
CST	BMI	TSMR - IVW	1043		-1.66	9.73E-02	1125.7	0.0359		
CST	BMI	TSMR - Egger	1043		0.26	7.98E-01	1124.8	0.0356	0.00	3.78E-01
CWM	DBP	GRS	836		-2.10	**3.59E-02**				
CWM	DBP	GSMR	1015		-1.56	1.20E-01				
CWM	DBP	RadialMR - with outliers - IVW	821	65.1	-1.83	6.68E-02	873.0	0.0972		
CWM	DBP	RadialMR - with outliers - Egger	821	65.1	0.32	7.47E-01	872.0	0.1012	-0.10	3.33E-01
CWM	DBP	RadialMR - no outliers - IVW	775	64.4	-1.19	2.32E-01	605.7	1.0000		
CWM	DBP	RadialMR - no outliers - Egger	775	64.4	1.49	1.37E-01	602.3	1.0000	-0.20	4.15E-02
CWM	DBP	TSMR - WM	877		0.23	8.21E-01				
CWM	DBP	TSMR - IVW	877		-1.69	9.17E-02	924.3	0.1251		
CWM	DBP	TSMR - Egger	877		0.27	7.84E-01	923.2	0.1254	0.00	3.13E-01
FX	WHR	GRS	525		2.04	**4.14E-02**				
FX	WHR	GSMR	627		2.51	**1.20E-02**				
FX	WHR	RadialMR - with outliers - IVW	511	69.3	2.78	**5.44E-03**	470.4	0.8949		
FX	WHR	RadialMR - with outliers - Egger	511	69.3	0.41	6.83E-01	470.1	0.8965	0.08	5.37E-01
FX	WHR	RadialMR - no outliers - IVW	485	69.3	2.46	**1.39E-02**	321.0	1.0000		
FX	WHR	RadialMR - no outliers - Egger	485	69.3	-0.06	9.49E-01	320.1	1.0000	0.13	2.41E-01
FX	WHR	TSMR - WM	542		1.35	1.77E-01				
FX	WHR	TSMR - IVW	542		2.66	**7.80E-03**	500.4	0.8937		
FX	WHR	TSMR - Egger	542		0.40	6.90E-01	499.9	0.8908	0.00	4.89E-01
MCP	LDL	GRS	546		2.40	**1.64E-02**				
MCP	LDL	GSMR	763		2.71	**6.69E-03**				
MCP	LDL	RadialMR - with outliers - IVW	521	222.3	2.02	**4.33E-02**	492.0	0.8065		
MCP	LDL	RadialMR - with outliers - Egger	521	222.3	1.35	1.78E-01	491.9	0.8066	-0.01	9.16E-01
MCP	LDL	RadialMR - no outliers - IVW	499	209.3	0.67	5.03E-01	380.6	1.0000		
MCP	LDL	RadialMR - no outliers - Egger	499	209.3	0.44	6.62E-01	380.6	1.0000	0.00	9.77E-01
MCP	LDL	TSMR - WM	598		1.19	2.34E-01				
MCP	LDL	TSMR - IVW	598		2.67	**7.59E-03**	583.7	0.6440		
MCP	LDL	TSMR - Egger	598		2.53	**1.15E-02**	582.7	0.6440	0.00	3.21E-01
MCP	BMI	GRS	1028		-2.86	**4.22E-03**				
MCP	BMI	GSMR	1179		-2.26	**2.37E-02**				
MCP	BMI	RadialMR - with outliers - IVW	1018	59.3	-2.35	**1.85E-02**	1029.1	0.3891		
MCP	BMI	RadialMR - with outliers - Egger	1018	59.3	-0.29	7.70E-01	1028.9	0.3903	-0.05	6.42E-01
MCP	BMI	RadialMR - no outliers - IVW	960	59.1	-1.51	1.32E-01	679.4	1.0000		
MCP	BMI	RadialMR - no outliers - Egger	960	59.1	0.62	5.33E-01	678.3	1.0000	-0.11	2.12E-01
MCP	BMI	TSMR - WM	1043		-1.48	1.38E-01				
MCP	BMI	TSMR - IVW	1043		-2.26	**2.41E-02**	1070.4	0.2637		
MCP	BMI	TSMR - Egger	1043		-0.78	4.35E-01	1070.4	0.2566	0.00	9.96E-01
PCR	WMH	GRS	23		2.09	**3.69E-02**				
PCR	WMH	GSMR	23		1.95	5.17E-02				
PCR	WMH	RadialMR - with outliers - IVW	22	55.0	1.37	1.70E-01	17.0	0.7102		
PCR	WMH	RadialMR - with outliers - Egger	22	55.0	0.48	6.36E-01	17.0	0.7110	-0.05	9.44E-01
PCR	WMH	RadialMR - no outliers - IVW	21	55.2	0.93	3.53E-01	12.7	0.8896		
PCR	WMH	RadialMR - no outliers - Egger	21	55.2	0.62	5.45E-01	12.6	0.8932	-0.19	7.62E-01
PCR	WMH	TSMR - WM	22		0.50	6.17E-01				
PCR	WMH	TSMR - IVW	22		1.01	3.13E-01	21.7	0.4142		
PCR	WMH	TSMR - Egger	22		0.67	5.08E-01	21.6	0.3623	-0.01	7.21E-01
PCR	SBP	GRS	809		-2.24	**2.51E-02**				
PCR	SBP	GSMR	973		-1.79	7.42E-02				
PCR	SBP	RadialMR - with outliers - IVW	785	63.8	-2.41	**1.58E-02**	853.1	0.0433		
PCR	SBP	RadialMR - with outliers - Egger	785	63.8	-0.29	7.69E-01	852.9	0.0437	-0.05	6.29E-01
PCR	SBP	RadialMR - no outliers - IVW	744	64.7	-2.36	**1.81E-02**	607.4	0.9999		
PCR	SBP	RadialMR - no outliers - Egger	744	64.7	-0.88	3.81E-01	607.4	0.9999	0.00	9.87E-01
PCR	SBP	TSMR - WM	817		-1.08	2.78E-01				

ROI	Exposure	Methods	N SNPs	Mean F	Z	p	Q	p(Q)	Egger intercept Beta	Egger intercept p
PCR	SBP	TSMR - IVW	817		-2.07	**3.87E-02**	891.9	0.0329		
PCR	SBP	TSMR - Egger	817		-0.31	7.57E-01	891.6	0.0316	0.00	6.05E-01
PCR	DBP	GRS	836		-2.98	**2.92E-03**				
PCR	DBP	GSMR	1012		-2.63	**8.46E-03**				
PCR	DBP	RadialMR - with outliers - IVW	821	65.1	-3.35	**8.01E-04**	861.8	0.1509		
PCR	DBP	RadialMR - with outliers - Egger	821	65.1	-0.44	6.62E-01	861.5	0.1528	-0.07	4.93E-01
PCR	DBP	RadialMR - no outliers - IVW	778	65.8	-3.53	**4.14E-04**	622.9	1.0000		
PCR	DBP	RadialMR - no outliers - Egger	778	65.8	-0.81	4.15E-01	622.7	1.0000	-0.05	5.87E-01
PCR	DBP	TSMR - WM	877		-1.38	1.66E-01				
PCR	DBP	TSMR - IVW	877		-3.24	**1.18E-03**	915.3	0.1735		
PCR	DBP	TSMR - Egger	877		-1.23	2.21E-01	915.3	0.1675	0.00	9.69E-01
PCR	T2D	GRS	241		-2.59	**9.57E-03**				
PCR	T2D	GSMR	282		-1.71	8.72E-02				
PCR	T2D	RadialMR - with outliers - IVW	243	72.0	-1.95	5.09E-02	258.7	0.2203		
PCR	T2D	RadialMR - with outliers - Egger	243	72.0	-0.97	3.32E-01	258.6	0.2214	0.03	8.29E-01
PCR	T2D	RadialMR - no outliers - IVW	233	73.1	-1.82	6.81E-02	207.4	0.8754		
PCR	T2D	RadialMR - no outliers - Egger	233	73.1	-0.89	3.74E-01	207.4	0.8756	0.01	9.23E-01
PCR	T2D	TSMR - WM	263		-1.02	3.09E-01				
PCR	T2D	TSMR - IVW	263		-2.16	**3.06E-02**	268.7	0.3750		
PCR	T2D	TSMR - Egger	263		-0.31	7.57E-01	268.1	0.3676	0.00	4.61E-01
PLIC	WMH	GRS	23		2.00	**4.54E-02**				
PLIC	WMH	GSMR	23		2.31	**2.09E-02**				
PLIC	WMH	RadialMR - with outliers - IVW	22	55.0	2.41	**1.59E-02**	15.9	0.7770		
PLIC	WMH	RadialMR - with outliers - Egger	22	55.0	2.20	**4.00E-02**	13.8	0.8766	-0.93	1.57E-01
PLIC	WMH	RadialMR - no outliers - IVW	21	55.4	1.87	6.17E-02	11.0	0.9452		
PLIC	WMH	RadialMR - no outliers - Egger	21	55.4	2.54	**1.99E-02**	9.0	0.9824	-0.98	7.78E-02
PLIC	WMH	TSMR - WM	22		1.64	1.02E-01				
PLIC	WMH	TSMR - IVW	22		2.42	**1.56E-02**	17.3	0.6911		
PLIC	WMH	TSMR - Egger	22		2.20	**3.94E-02**	15.1	0.7678	-0.03	1.56E-01
PLIC	WHR	GRS	525		-2.31	**2.07E-02**				
PLIC	WHR	GSMR	628		-2.81	**4.92E-03**				
PLIC	WHR	RadialMR - with outliers - IVW	511	69.3	-3.11	**1.89E-03**	500.9	0.6054		
PLIC	WHR	RadialMR - with outliers - Egger	511	69.3	-2.86	**4.44E-03**	496.5	0.6572	0.24	5.80E-02
PLIC	WHR	RadialMR - no outliers - IVW	493	69.3	-3.12	**1.82E-03**	394.2	0.9996		
PLIC	WHR	RadialMR - no outliers - Egger	493	69.3	-2.64	**8.54E-03**	391.9	0.9997	0.18	1.25E-01
PLIC	WHR	TSMR - WM	542		-2.47	**1.37E-02**				
PLIC	WHR	TSMR - IVW	542		-2.99	**2.76E-03**	534.8	0.5676		
PLIC	WHR	TSMR - Egger	542		-3.78	**1.77E-04**	526.7	0.6505	0.01	4.80E-03
SCC	HDL	GRS	513		-2.51	**1.21E-02**				
SCC	HDL	GSMR	690		-2.32	**2.02E-02**				
SCC	HDL	RadialMR - with outliers - IVW	484	252.7	-2.89	**3.89E-03**	472.8	0.6219		
SCC	HDL	RadialMR - with outliers - Egger	484	252.7	-2.19	**2.87E-02**	472.4	0.6260	0.04	5.69E-01
SCC	HDL	RadialMR - no outliers - IVW	459	250.5	-2.63	**8.61E-03**	345.7	1.0000		
SCC	HDL	RadialMR - no outliers - Egger	459	250.5	-2.48	**1.36E-02**	345.1	1.0000	0.06	4.04E-01
SCC	HDL	TSMR - WM	549		-2.08	**3.73E-02**				
SCC	HDL	TSMR - IVW	549		-2.54	**1.11E-02**	558.7	0.3665		
SCC	HDL	TSMR - Egger	549		-1.93	5.39E-02	558.5	0.3570	0.00	6.91E-01
SCP	PP	GRS	666		2.00	**4.55E-02**				
SCP	PP	GSMR	790		1.10	2.71E-01				
SCP	PP	RadialMR - with outliers - IVW	651	64.0	1.85	6.39E-02	634.0	0.6657		
SCP	PP	RadialMR - with outliers - Egger	651	64.0	-0.41	6.86E-01	633.0	0.6766	0.13	2.90E-01
SCP	PP	RadialMR - no outliers - IVW	616	63.5	1.90	5.71E-02	439.8	1.0000		
SCP	PP	RadialMR - no outliers - Egger	616	63.5	-0.49	6.22E-01	438.6	1.0000	0.14	2.02E-01
SCP	PP	TSMR - WM	681		1.24	2.14E-01				
SCP	PP	TSMR - IVW	681		1.86	6.28E-02	654.9	0.7485		
SCP	PP	TSMR - Egger	681		0.02	9.83E-01	654.4	0.7446	0.00	4.64E-01
SCP	BMI	GRS	1028		-2.57	**1.01E-02**				
SCP	BMI	GSMR	1183		-3.22	**1.28E-03**				
SCP	BMI	RadialMR - with outliers - IVW	1018	59.3	-2.01	**4.41E-02**	1021.6	0.4539		
SCP	BMI	RadialMR - with outliers - Egger	1018	59.3	-0.40	6.89E-01	1021.5	0.4540	-0.02	8.10E-01
SCP	BMI	RadialMR - no outliers - IVW	964	59.5	-1.55	1.22E-01	700.3	1.0000		
SCP	BMI	RadialMR - no outliers - Egger	964	59.5	-0.50	6.15E-01	700.3	1.0000	-0.01	9.37E-01
SCP	BMI	TSMR - WM	1043		-1.73	8.43E-02				
SCP	BMI	TSMR - IVW	1043		-1.93	5.40E-02	1035.7	0.5493		
SCP	BMI	TSMR - Egger	1043		-0.92	3.58E-01	1035.6	0.5413	0.00	7.85E-01
SCR	WMH	GRS	23		2.50	**1.24E-02**				
SCR	WMH	GSMR	23		3.15	**1.62E-03**				
SCR	WMH	RadialMR - with outliers - IVW	22	55.0	2.65	**8.02E-03**	17.4	0.6855		

									Egger intercept	
ROI	Exposure	Methods	N SNPs	Mean F	Z	p	Q	p(Q)	Beta	p
SCR	WMH	RadialMR - with outliers - Egger	22	55.0	1.09	2.89E-01	17.2	0.6966	-0.23	7.53E-01
SCR	WMH	RadialMR - no outliers - IVW	21	55.7	3.02	**2.55E-03**	12.0	0.9176		
SCR	WMH	RadialMR - no outliers - Egger	21	55.7	1.00	3.31E-01	12.0	0.9173	0.04	9.46E-01
SCR	WMH	TSMR - WM	22		1.90	5.70E-02				
SCR	WMH	TSMR - IVW	22		2.71	**6.65E-03**	19.0	0.5834		
SCR	WMH	TSMR - Egger	22		0.83	4.17E-01	19.0	0.5206	0.00	9.30E-01
SFO	PP	GRS	666		2.15	**3.17E-02**				
SFO	PP	GSMR	792		2.56	**1.06E-02**				
SFO	PP	RadialMR - with outliers - IVW	651	64.0	2.21	**2.72E-02**	670.0	0.2856		
SFO	PP	RadialMR - with outliers - Egger	651	64.0	0.25	8.04E-01	669.8	0.2871	0.06	6.39E-01
SFO	PP	RadialMR - no outliers - IVW	613	63.9	1.55	1.21E-01	450.4	1.0000		
SFO	PP	RadialMR - no outliers - Egger	613	63.9	0.82	4.15E-01	450.3	1.0000	-0.03	8.00E-01
SFO	PP	TSMR - WM	681		1.10	2.73E-01				
SFO	PP	TSMR - IVW	681		2.27	**2.32E-02**	695.5	0.3318		
SFO	PP	TSMR - Egger	681		-0.10	9.22E-01	694.4	0.3325	0.00	3.04E-01
SS	WMH	GRS	23		2.60	**9.33E-03**				
SS	WMH	GSMR	23		2.64	**8.41E-03**				
SS	WMH	RadialMR - with outliers - IVW	22	55.0	2.71	**6.78E-03**	24.2	0.2817		
SS	WMH	RadialMR - with outliers - Egger	22	55.0	0.54	5.93E-01	24.3	0.2804	0.12	8.92E-01
SS	WMH	RadialMR - no outliers - IVW	20	56.1	2.48	**1.33E-02**	15.2	0.7103		
SS	WMH	RadialMR - no outliers - Egger	20	56.1	0.35	7.33E-01	15.1	0.7170	0.32	6.60E-01
SS	WMH	TSMR - WM	22		1.51	1.30E-01				
SS	WMH	TSMR - IVW	22		2.73	**6.43E-03**	24.2	0.2829		
SS	WMH	TSMR - Egger	22		0.72	4.81E-01	24.2	0.2352	0.00	8.51E-01
SS	DBP	GRS	836		-2.31	**2.06E-02**				
SS	DBP	GSMR	1016		-2.63	**8.42E-03**				
SS	DBP	RadialMR - with outliers - IVW	821	65.1	-3.42	**6.37E-04**	815.3	0.5397		
SS	DBP	RadialMR - with outliers - Egger	821	65.1	-1.17	2.41E-01	815.3	0.5399	0.00	9.69E-01
SS	DBP	RadialMR - no outliers - IVW	777	65.6	-2.93	**3.34E-03**	568.8	1.0000		
SS	DBP	RadialMR - no outliers - Egger	777	65.6	-1.25	2.13E-01	568.8	1.0000	0.01	9.19E-01
SS	DBP	TSMR - WM	877		-2.59	**9.64E-03**				
SS	DBP	TSMR - IVW	877		-2.83	**4.61E-03**	839.5	0.8075		
SS	DBP	TSMR - Egger	877		-1.85	6.49E-02	838.8	0.8053	0.00	4.18E-01
TAP	DBP	GRS	836		-2.48	**1.31E-02**				
TAP	DBP	GSMR	1014		-1.61	1.08E-01				
TAP	DBP	RadialMR - with outliers - IVW	821	65.1	-2.58	**9.84E-03**	853.9	0.2000		
TAP	DBP	RadialMR - with outliers - Egger	821	65.1	-2.18	**2.96E-02**	851.2	0.2186	0.15	1.55E-01
TAP	DBP	RadialMR - no outliers - IVW	774	63.6	-1.44	1.51E-01	574.1	1.0000		
TAP	DBP	RadialMR - no outliers - Egger	774	63.6	-1.08	2.80E-01	573.8	1.0000	0.06	5.64E-01
TAP	DBP	TSMR - WM	877		-1.54	1.24E-01				
TAP	DBP	TSMR - IVW	877		-2.25	**2.43E-02**	912.3	0.1920		
TAP	DBP	TSMR - Egger	877		-3.26	**1.16E-03**	905.3	0.2317	0.01	9.92E-03
UNC	LDL	GRS	546		-2.72	**6.48E-03**				
UNC	LDL	GSMR	765		-1.80	7.11E-02				
UNC	LDL	RadialMR - with outliers - IVW	521	222.3	-1.80	7.22E-02	534.1	0.3249		
UNC	LDL	RadialMR - with outliers - Egger	521	222.3	0.12	9.05E-01	531.8	0.3511	-0.11	1.29E-01
UNC	LDL	RadialMR - no outliers - IVW	496	226.2	-1.29	1.98E-01	393.2	0.9997		
UNC	LDL	RadialMR - no outliers - Egger	496	226.2	0.08	9.40E-01	392.0	0.9998	-0.08	2.22E-01
UNC	LDL	TSMR - WM	598		-0.81	4.20E-01				
UNC	LDL	TSMR - IVW	598		-1.83	6.67E-02	617.3	0.2746		
UNC	LDL	TSMR - Egger	598		0.75	4.52E-01	609.8	0.3388	-0.01	7.09E-03

Only results with p<0.05 for genetic risk score (GRS) analyses are shown. Significant results ($p<1.27x10^{-3}$) are in blue. Nominally significant (p<0.05) results are in bold.

WMH: White Matter Hyperintensities; SBP: Systolic blood pressure; DBP: Diastolic blood pressure; PP: Pulse pressure; HDL: HDL-cholesterol; LDL: LDL-cholesterol; T2D: Type 2 diabetes; TG: Triglycerides; BMI: Body Mass Index; WHR: Waist-Hip Ratio. GSMR: Generalised Summary-data-based MR. TSMR: TwoSampleMR; IVW: inverse variance weighted; WM: weighted median. Z: Z-score defined as Beta / Standard error.

NDI: Neurite Density Index; ODI: Orientation Dispersion Index; ISOVF: Isotropic Volume Fraction
